# Live-attenuated influenza virus vaccine strain with an engineered temperature-sensitive and genetically stable viral polymerase variant

**DOI:** 10.1128/jvi.01390-25

**Published:** 2025-11-13

**Authors:** Tadasuke Naito, Hiroshi Ushirogawa, Miyuki Kunishio, Haruka Yano, Susumu Saito, Taisei Higeuchi, Kazuki Fujita, Mineki Saito

**Affiliations:** 1 Department of Microbiology, Kawasaki Medical School, Okayama, Japan; St Jude Children's Research Hospital, Memphis, Tennessee, USA

**Keywords:** temperature-sensitive phenotype, viral RNA polymerase, live-attenuated vaccine, influenza virus, reverse genetic analysis

## Abstract

Vaccination against seasonal influenza is considered an effective means of reducing morbidity and mortality. Live-attenuated vaccines offer more protection against influenza than inactivated vaccines as they efficiently induce cellular immunity and provide cross-immunogenicity against various antigenic subtypes. For the production of safer live-attenuated vaccines, it is important to develop a common master donor vaccine strain in which pathogenic revertants are much less likely to appear. In this study, we introduced a single amino acid substitution of Lys471 into the PB1 polymerase subunit of influenza A virus and succeeded in isolating an attenuated mutant virus that exhibits a temperature-sensitive phenotype. The Lys471 residue is located in the polymerase motif D on PB1 and is positioned near the entrance tunnel domain for incoming nucleotide triphosphate. Although 10 viable PB1-Lys471 mutants did not proliferate at 37°C, their variants could replicate at 31°C and 34°C. Moreover, we found that PB1-Lys471Pro variant induces a genetically stable influenza virus phenotype; this mutant virus did not revert to wild-type phenotype from the temperature-sensitive phenotype by serial virus passages. Animal experiments have demonstrated that these PB1 mutant strains work effectively as live-attenuated vaccines. Application of the PB1-Lys471 substitution to a master donor strain is expected to lead to the development of a safer, high-performance, and widely used live-attenuated vaccine with the antigen of circulating influenza viruses.

**IMPORTANCE:**

Influenza virus elicits respiratory tract disease and is a threat to global human health. Vaccination is considered an effective tool for reducing the morbidity and mortality caused by influenza disease. The only licensed live-attenuated influenza vaccine that has been proven safe and effective is FluMist. In this study, we isolated an attenuated influenza mutant virus with a Lys471 single amino acid substitution in PB1, which displayed a temperature-sensitive and a low-pathogenicity phenotype. By applying the PB1-Lys471 substitution to the vaccine mother strain or the circulating influenza virus using reverse genetic technology, a high-performance and safe live-attenuated vaccine carrying the viral antigens of the vaccine-targeted strain can be developed.

## INTRODUCTION

Multiple types of seasonal influenza vaccine are available, that is, inactivated influenza vaccines include an egg-based, a cell culture-based, a recombinant protein-based, and an egg-based live-attenuated influenza vaccines (LAIVs) (1). These vaccines, which protect against four different influenza strains, include two influenza A viruses (H1N1 and H3N2) and two influenza B lineage viruses. Although administration of inactivated influenza vaccines induces a protective serum antibody response, LAIVs have additional benefits of inducing mucosal and cell-mediated immunity. Therefore, LAIV confers greater breadth of protection against antigenic mutants of influenza virus. LAIV strain has to have a temperature-sensitive phenotype because the temperature in the nasal passages tends to be a few degrees cooler than the body temperature of the lower respiratory tract. The temperature-sensitive LAIV administered intranasally proliferates in the nasal mucosa and induces immunity but does not develop influenza symptoms because those vaccine strains showed an attenuated phenotype and restricted ability to replicate in the lungs. Thus, the LAIV strain replicates locally in the upper respiratory tract without causing clinical illness and induces effective immunity for protection against influenza virus infection.

Influenza A viruses belong to *Orthomyxoviridae*, a family of enveloped viruses with negative-sense RNA viruses. The influenza virus genome comprises eight segmented and single-stranded RNAs. It also possesses a heterotrimeric RNA-dependent RNA polymerase (RdRp) composed of PB1 (polymerase basic protein 1), PB2 (polymerase basic protein 2), and PA (polymerase acidic protein) subunits (2, 3). Each of the viral genome segments is formed as a distinct ribonucleoprotein by oligomerization of the viral nucleoprotein (NP) with the RdRp complex. The PB1 subunit is at the center of the polymerase complex and contains polymerase motifs that are common to RdRps. Recently, crystallographic and high-resolution cryo-electron microscopy structures of the complete heterotrimer of influenza virus polymerase have been determined (4, 5), providing insights to aid in the understanding of the multifunctional RNA synthesis machinery involved in viral genome replication and transcription.

RNA viruses encode their own RdRp to synthesize their viral genome and mRNA within infected cells. The overall structure of RdRp resembles a cupped right hand including finger, palm, and thumb domains and catalyzes phosphodiester bond formation through a conserved two-metal ion mechanism (6). Six structural motifs, designated A to F, have been identified in RdRps. The catalytic motif A to E and motif F are distributed within the palm and finger domains, respectively. This architecture is shared with DNA-dependent DNA polymerases, DNA-dependent RNA polymerases, and reverse transcriptases and plays critical roles in the enzymatic function of polymerases (7–9). These motifs exert important actions in the binding of metal ions, nucleoside triphosphate, and RNA, which are critical for the nucleotidyltransferase reaction catalyzed by RdRp.

Amino acid substitution(s) in the polymerase subunits of influenza virus is reported to induce variants with various phenotypes, such as temperature-sensitive or fidelity-changing mutants (10–14). The influenza A/Ann Arbor/6/60 (A/AA/6/60) H2N2 cold-adapted virus was isolated by *in vitro* serial passage of the wild-type A/AA/6/60 virus at successively lower temperatures in the 1960s (11, 15–17). The cold-adapted A/AA/6/60 virus also acquired temperature-sensitive and attenuated phenotypes. To determine the viral genes responsible for the temperature-sensitive phenotypes of the A/AA/6/60 mutant, reassortant viruses between cold-adapted A/AA/6/60 and other viruses were examined, and these studies indicated that the PB1, PB2, and NP segments contributed to the temperature-sensitive phenotypes. Furthermore, site-directed mutagenesis and reverse genetic analysis mapped the temperature-sensitive phenotype of A/AA/6/60 to the following five major loci: PB1-K391E/E581G/A661T, PB2-N265S, and NP-D34G (18). The licensed LAIV is prepared by generating a reassortant containing six internal genes from a cold-adapted A/AA/6/60 and two surface glycoprotein genes, hemagglutinin (HA) and neuraminidase (NA), from the circulating strain. The HA and NA proteins are targets of the protective immune response. This cold-adapted A/AA/6/60-based LAIV is marketed as FluMist and the product name in Europe is Fluenz Tetra. FluMist can elicit IgA mucosal immunity and cellular immunity against HA and NA antigens derived from the circulating virus.

**Fig 1:**
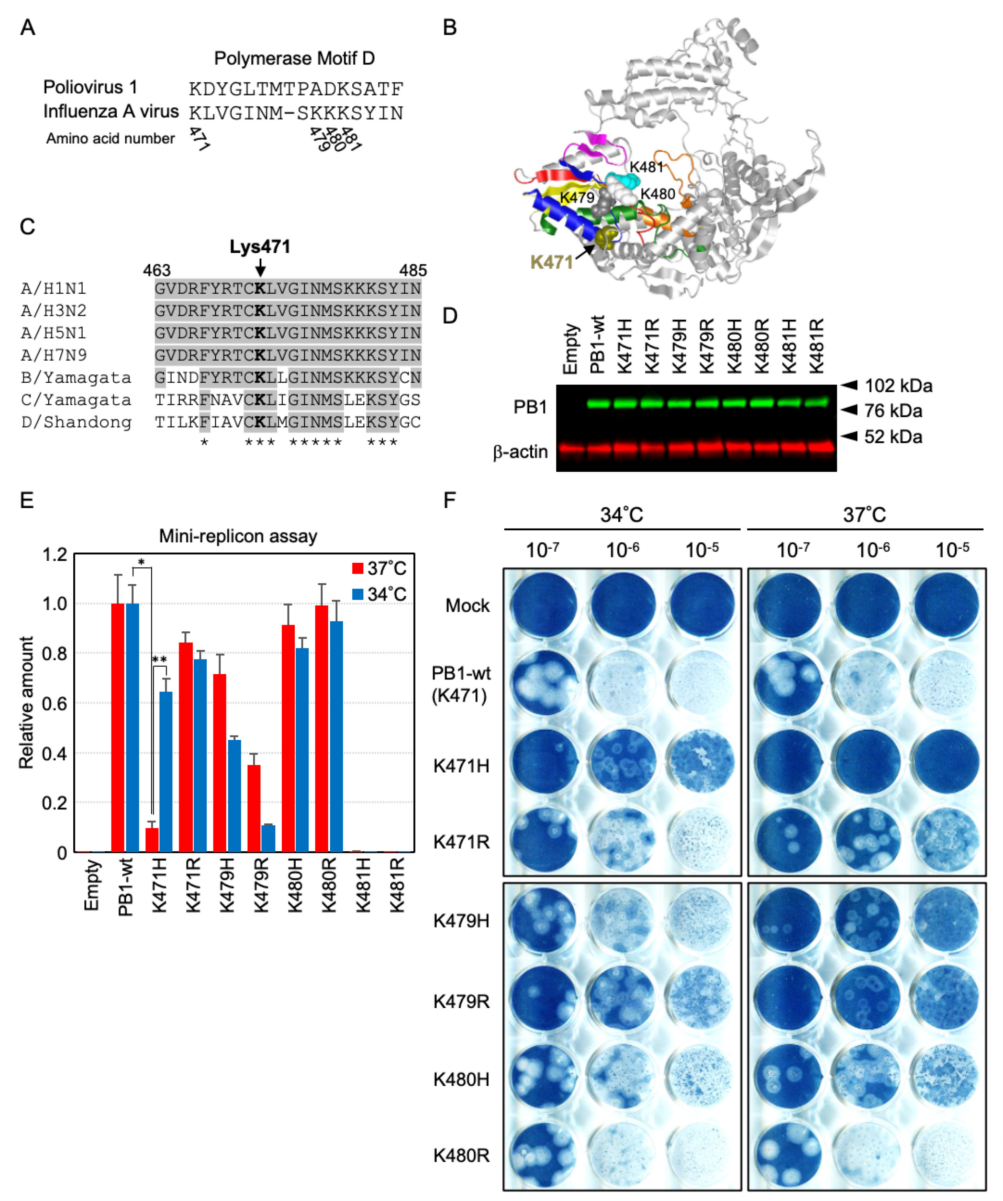
A Lys residue on polymerase motif D of PB1 determines the viral RNA synthesis activity. (**A**) Sequence alignments of motif D between poliovirus and influenza A virus RNA polymerase. (**B**) Cartoon representation of the influenza virus PB1 structure. Polymerase motif A, B, C, D, E, and F are represented in red, green, yellow, blue, magenta, and orange, respectively. PB1-Lys471 (olive), PB1-Lys479 (dark gray), PB1-Lys480 (white), and PB1-Lys481 (cyan) are represented using the space-filling Corey-Pauling-Koltun model. (**C**) The Lys471 residue is conserved in the PB1 viral polymerase subunit. Sequence alignments of the 463 to 485 amino acid regions from the indicated viral PB1 subunits are shown. The conserved residues across the strains are indicated by asterisks and background color. The Lys471 residue is indicated in bold. The referenced strains were as follows: A/H1N1, A/Brisbane/2015/2009; A/H3N2, A/Kansas/9506/2019; A/H5N1, A/chicken/Indonesia/11/2003; A/H7N9, A/Anhui/1-DEWH730/2013; B/Yamagata, B/Yamagata/16/1988; C/Yamagata, C/Yamagata/35/2014; D/Shandong, D/bovine/Shandong/Y127/2014. (**D**) Western blotting analysis of PR8-PB1 mutant proteins in 293T cells. The 293T cells were transfected with pCAGGS-PB1-wild-type (PB1-wt) or pCAGGS-PB1 mutants. Cells were incubated at 37°C for 24 h, and were harvested and subsequently assayed for Western blotting. Transfected PB1 proteins were confirmed with anti-PB1 polyclonal antibodies, and equal sample loading was verified with anti-β-actin monoclonal antibody. (**E**) The effect of Lys residue substitution on viral RNA polymerase activity using a mini-replicon reporter assay system, for influenza virus genome replication. The 293T cells were transfected with RL-SV40, pHH-vNS-Luc, pCAGGS-PB2, pCAGGS-PA, pCAGGS-NP, and either pCAGGS-PB1-wild-type (PB1-wt) or pCAGGS-PB1 mutants. Cells were incubated at 34°C or 37°C for 24 h and were harvested and subsequently assayed for luciferase activity. The luciferase activity was normalized to the Renilla luciferase activity. At each temperature, the Firefly/Renilla relative activity of PB1-wt polymerase was set as 1.0, and relative activity of PB1 mutants was expressed. Quantitative results are presented as the average with the standard deviation (SD) from at least three independent experiments. Significance was determined using Student’s *t*-test (*, *P* = 3.6 × 10^−3^; **, *P* = 8.0 × 10^−4^). (**F**) A K471H mutation in PB1 polymerase induces a temperature-sensitive influenza virus. Plaque morphology of PR8-PB1 mutants. Plaque assay was performed using an amplified version of each virus in chicken eggs. Serial dilutions (10^−7^ to 10^−5^) of the PB1-wild-type or PB1 mutant viruses were used to infect MDCK cells at the indicated temperatures. MDCK cells were infected with the viruses indicated at the left of the figure and incubated at 34°C or 37°C for 4 or 5 days. Virus plaques were visualized by Amido Black 10B staining and photographed.

Recently, we examined the functional importance of a Lys residue involving a nucleotidyltransferase reaction in the polymerase motif D of influenza virus PB1 polymerase, by referencing an investigation of poliovirus polymerase variants (12). As a result, a Lys-to-Arg or Lys-to-His single amino acid substitution at position 481 in the PB1 subunit of the egg-adapted A/Puerto Rico/8/1934/H1N1 (PR8) influenza strain for vaccine production was identified as a lethal mutation. On the other hand, in a preliminary study, we observed that the Lys471 residue on polymerase motif D of PB1 is important for temperature sensitivity during viral growth. We hypothesized that the PB1-K471 residue, a basic amino acid conserved in the polymerase motif D between the poliovirus and the influenza virus, plays a crucial role in regulating polymerase function (Fig. 1A). In the present study, we attempted to characterize the PR8-PB1-Lys471 mutant viruses with a temperature-sensitive phenotype and application to an effective LAIV, whose replication of wild type is inhibited by vaccination using PB1-K471 variants in an animal challenge model of influenza virus infection.

## RESULTS

### A K471H mutation in PB1 polymerase induces a temperature-sensitive influenza virus

The polymerase motif D sequence of the poliovirus 3D^pol^ and influenza virus PB1 subunit were aligned as shown in Fig. 1A. The Lys471 residue on polymerase motif D of the PB1 subunit is located near the active site for nucleotide incorporation in the influenza virus polymerase complex (Fig. 1B) (4, 5). We aligned PB1 amino acids 463 to 485 in various strains, as shown in Fig. 1C. The Lys471 residue of PB1 was found to be extremely conserved. We thus hypothesized that the Lys residue in polymerase motif D might be important for the modulation of polymerase activity involving virus growth. Firstly, we tested whether a Lys-to-Arg or Lys-to-His substitution at Lys471 in the PB1 of the PR8 strain would affect its temperature sensitivity and viral polymerase activity. We also analyzed the effects of substitution in the Lys479, Lys480, and Lys481 amino acids present in polymerase motif D. Polymerase activity was evaluated using a mini-replicon reporter assay for influenza virus genome replication. To examine whether substitution of Lys residues in polymerase motif D of PB1 of the PR8 strain conferred a temperature-sensitive phenotype on the modified viral polymerase complex, a mini-replicon assay was performed at 37°C and at 34°C. Then, 293T cells in 12-well plates were transfected with each plasmid carrying the gene encoding PB2, PA, or NP, and the PB1-wild-type or PB1 mutant plasmids, together with the expression plasmid of the model viral genome encoding a luciferase gene.

We constructed PB1 mutant expression plasmids for mammalian cells and confirmed the synthesis of these mutant proteins by Western blot analysis (Fig. 1D). As shown in Fig. 1E, the level of viral RNA synthesis from the PB1-K471R mutant polymerase was reduced to 84% at 37°C and to 76% at 34°C compared to that from the PB1-wild type. Introduction of K471H into PB1 resulted in viral RNA synthesis reduced to 64% at 34°C compared to that with the parental PR8; in contrast, there was a 90% reduction at 37°C compared to that in the PB1-wild type (Fig. 1E, *P = 3.6 × 10^−3^ by Student’s *t*-test). Overall, the polymerase activity of PB1-K471H was increased 6.7-fold at 34°C compared to that at 37°C (Fig. 1E, **P = 8.0 × 10^−4^ by Student’s *t*-test). The level of viral RNA synthesis from PB1-K479R/H or PB1-K480R/H substitutions was reduced compared to that in the PB1-wild type; however, these mutated polymerases did not induce a temperature-sensitive phenotype. Further, PB1 activity was almost lost following K481R or K481H substitution at both 34°C and 37°C. These results suggest that the PB1-K471H substitution produced a viral polymerase whose genome replication ability was impaired in a temperature-dependent manner.

To compare the temperature-sensitive viral replication activities of PB1 variants, we generated reassortant viruses with K471H, K471R, K479H, K479R, K480H, or K480R substitutions in the PB1 segment using a reverse genetics approach. In this study, temperature-sensitive phenotype is defined as mutant viruses that did not form viral plaques at 37°C and had reduced plaque formation ability at 34°C compared to the PR8-wild type. Propagation of each virus in chicken eggs was examined using the plaque and hemagglutination (HA) assay (Fig. 1F and Table 1), and the HA titers of PR8-PB1-wild type and PB1 mutant strains were 1,024 to 4,096 HA units. The temperature-sensitive phenotypes of these PB1 variants were examined by plaque assays on MDCK cells at 34°C or 37°C. The PB1-wild-type virus did not exhibit the temperature-sensitive phenotype; the difference in the titers at 34°C and 37°C was only 0.24 log_10_ PFU/mL. Although the virus titer reductions were not greater than 1.0 log_10_ PFU/mL at 34°C or 37°C for any of the PB1 mutants compared to the PB1-wild type (e.g., PB1-K471R was reduced to 0.32 log_10_ PFU/mL or 0.57 log_10_ PFU/mL at 34°C or 37°C compared to the PB1-wild type), PB1-K471H, PB1-K471R, PB1-K479H, and PB1-K479R variants formed smaller plaques. In particular, a significant reduction was observed in plaque formation at 37°C for the PB1-K471H virus. This result indicated that a Lys-to-His substitution at position 471 in PB1 could induce a temperature-sensitive influenza virus phenotype.

**TABLE 1:** Growth kinetics of PR8-PB1 mutant viruses in eggs

Test virus	HA titer^*a*^	PFU/mL of E1 viruses^*b*^ at	
		34°C	37°C
PB1-wild type	2,048	1.1 ± 0.2 × 10^9^	6.3 ± 2.0 × 10^8^
PB1-K471H	1,024	2.0 ± 0.5 × 10^8^	ND^*c*^
PB1-K471R	2,048	5.3 ± 0.7 × 10^8^	1.7 ± 0.3 × 10^8^
PB1-K479H	4,096	9.3 ± 0.7 × 10^8^	1.8 ± 0.2 × 10^8^
PB1-K479R	1,024	3.3 ± 0.3 × 10^8^	1.7 ± 0.2 × 10^8^
PB1-K480H	1,024	8.3 ± 1.6 × 10^8^	3.3 ± 0.3 × 10^8^
PB1-K480R	1,024	7.3 ± 2.6 × 10^8^	4.7 ± 0.3 × 10^8^

^*a*^ HA titers of viruses isolated from eggs were determined by HA assay.

^*b*^ PFU/mL of viruses isolated from eggs were determined by plaque assay. The number of plaques was counted following Amido Black 10B staining after 4 to 5 days of inoculation at 34°C or 37°C. Data are means from three independent experiments. Errors are represented as standard error.

^*c*^ ND, not detected. ND means that no plaque formation was observed in a plaque assay using 1 mL of undiluted virus stock.

### A point mutation in the PB1-Lys471 residue influences virus growth and a temperature-sensitive phenotype

**Fig 2:**
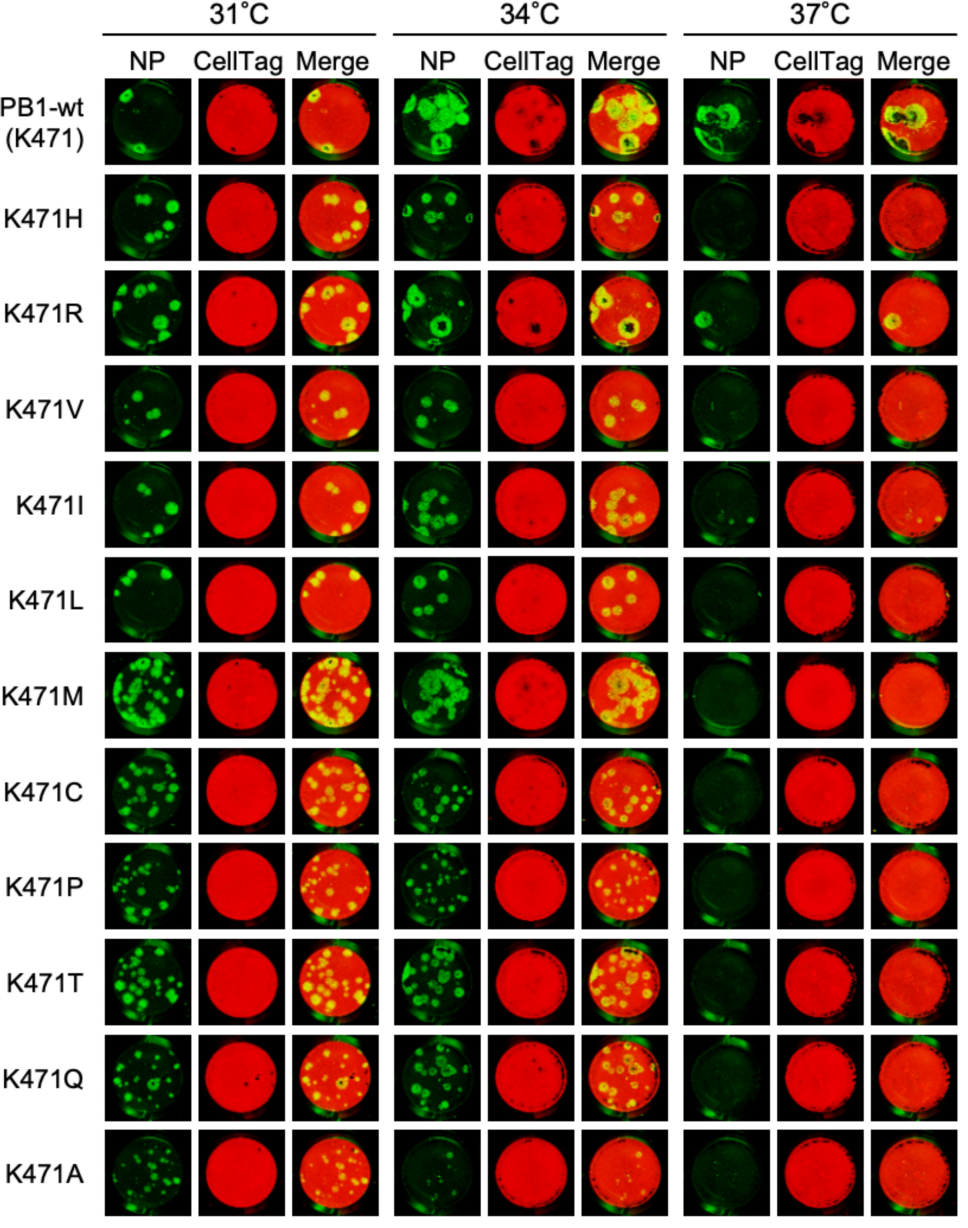
Effect of PB1-K471 mutation involved in temperature-sensitive phenotype. Plaque morphology of PR8-PB1-K471 mutants. The PB1-wild type or PB1-K471 mutant viruses were used to infect MDCK cells at the indicated temperatures. MDCK cells were infected with the viruses indicated at the left of the figure and incubated at 31°C, 34°C, or 37°C for 3 days. Virus plaques and MDCK cells were visualized by immunostaining using anti-NP antibody and CellTag700, respectively. The virus plaque and MDCK cells were detected with the Odyssey CLx Infrared Imaging System.

We hypothesized that PB1-Lys471 plays an important role in modulating temperature sensitivity in viral proliferation. To test this, we generate recombinant viruses and expression plasmids in which K471 was replaced with each of the other amino acids, and viral growth, RNA polymerase activity, and temperature sensitivity of the mutant were assessed in comparison with the PR8-PB1-wild-type virus. First, we tested whether each mutant virus was generated by using a reverse genetic system. Eight recombinant viruses were not found to synthesize in a reverse genetic approach using plasmid-transfected 293T cells (Table 2, “PFU/mL of seed viruses” column, indicated as not detected [ND]). Propagation of the viable mutant virus in chicken eggs was examined by HA assay and plaque assay. HA titers of viable PB1-K471 mutants were 512 to 2,048 HA units (Table 2, “HA titer of E1 viruses” column). These PB1-K471 mutants detected obvious plaque formation at 31°C or 34°C using an immunostaining method (Fig. 2). Ten PB1 recombinant viruses, PB1-K471H(His), K471V(Val), K471I(Ile), K471L(Leu), K471M(Met), K471C(Cys), K471P(Pro), K471T(Thr), K471Q(Gln), and K471A(Ala), did not generate plaques at 37°C; therefore, these substitutions induce a temperature sensitivity in viral replication.

**TABLE 2:** Effect of substitution of Lys471 residue on viral proliferation

Test virus	PFU/mL of seed viruses^*a*^	HA titer of E1 viruses^*c*^	PFU/mL of E1 viruses^*e*^ at		
			31°C	34°C	37°C
PB1-wild type (K471)	6.7 ± 0.3 × 10^4^	2,048	5.3 ± 0.2 × 10^8^	7.3 ± 0.2 × 10^8^	2.5 ± 0.06 × 10^8^
PB1-K471H	2.6 ± 0.2 × 10^3^	1,024	1.1 ± 0.09 × 10^8^	2.3 ± 0.06 × 10^8^	ND
PB1-K471R	4.6 ± 0.3 × 10^4^	2,048	2.1 ± 0.1 × 10^8^	2.1 ± 0.09 × 10^8^	9.3 ± 0.6 × 10^7^
PB1-K471V	1.4 ± 0.3 × 10^4^	1,024	3.2 ± 0.03 × 10^8^	5.3 ± 0.06 × 10^8^	ND
PB1-K471I	9.0 ± 1.0 × 10^3^	1,024	3.5 ± 0.06 × 10^8^	7.3 ± 0.4 × 10^8^	NC^*f*^
PB1-K471L	4.3 ± 0.3 × 10^3^	1,024	8.0 ± 0.1 × 10^7^	1.3 ± 0.2 × 10^8^	ND
PB1-K471M	3.5 ± 0.3 × 10^3^	1,024	2.2 ± 0.03 × 10^8^	3.7 ± 0.3 × 10^8^	ND
PB1-K471C	6.3 ± 0.9 × 10^2^	512	8.3 ± 0.2 × 10^7^	4.0 ± 0.3 × 10^7^	ND
PB1-K471P	6.7 ± 1.3 × 10^2^	1,024	2.3 ± 0.06 × 10^8^	2.3 ± 0.06 × 10^8^	ND
PB1-K471T	1.9 ± 0.1 × 10^3^	1,024	5.3 ± 0.06 × 10^7^	1.1 ± 0.04 × 10^8^	ND
PB1-K471Q	4.7 ± 0.3 × 10^2^	1,024	3.7 ± 0.06 × 10^7^	2.7 ± 0.06 × 10^7^	ND
PB1-K471A	2.3 ± 0.3 × 10^2^	1,024	8.7 ± 0.2 × 10^7^	9.0 ± 0.2 × 10^7^	ND
PB1-K471F	ND^*b*^	NT^*d*^	NT	NT	NT
PB1-K471Y	ND	NT	NT	NT	NT
PB1-K471G	ND	NT	NT	NT	NT
PB1-K471S	ND	NT	NT	NT	NT
PB1-K471N	ND	NT	NT	NT	NT
PB1-K471D	ND	NT	NT	NT	NT
PB1-K471E	ND	NT	NT	NT	NT
PB1-K471W	ND	NT	NT	NT	NT

^*a*^ The PFU/mL was determined using virus particles that generated from reverse genetics plasmid transfected 293T cells. The number of plaques was counted following Amid Black 10B staining after 4 days of inoculation at 34°C. Data are means from three independent experiments. Errors are represented as standard error.

^*b*^ ND, not detected. ND means that no plaque formation was observed in a plaque assay using 1 mL of undiluted virus stock.

^*c*^ HA titers were determined using viruses amplified in egg (abbreviated here as E1) that were infected with 200 PFU of seed viruses generated by RG system.

^*d*^ NT, not tested.

^*e*^ PFU/mL were determined using viruses amplified in egg (abbreviated here as E1) that were infected with 200 PFU of seed viruses generated by RG system. The number of plaques was counted following immunostaining after 3 days of inoculation at 31°C, 34°C, or 37°C. Data are means from three independent experiments. Errors are represented as standard error.

^*f*^ NC, not calculated. PB1-K471I could not form a clear viral plaque at 37°C incubation.

**Fig 3:**
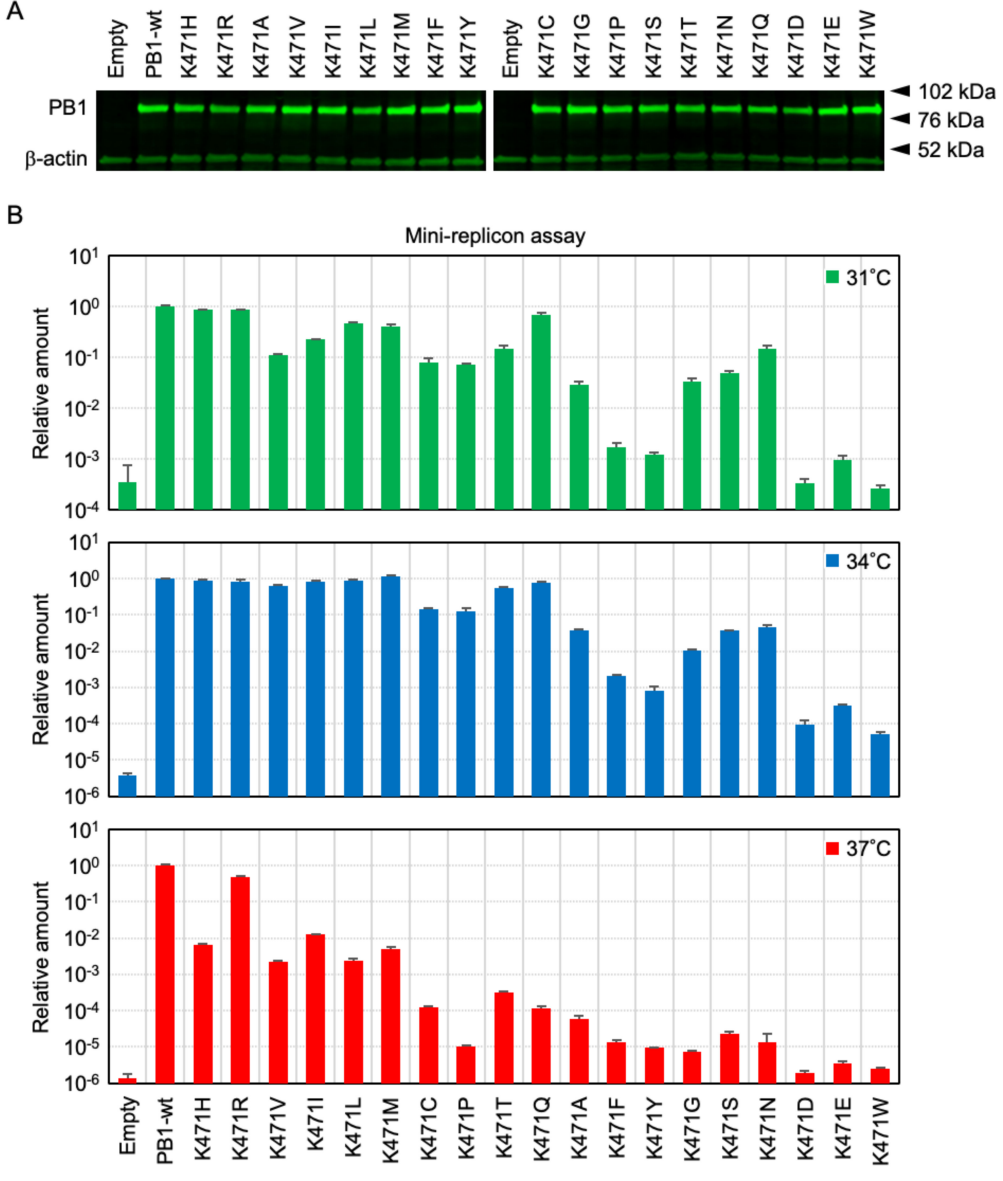
Importance of the PB1-Lys471 residue in viral RNA synthesis. (**A**) Western blotting analysis of PB1-K471 mutant proteins in 293T cells. Transfected PB1 proteins were confirmed with anti-PB1 polyclonal antibodies, and equal sample loading was verified with anti-β-actin polyclonal antibody. (**B**) The effect of K471 amino acid substitution on viral RNA polymerase activity was measured using a reporter assay system for the influenza genome replication. The 293T cells were transfected with pRL-SV40, pHH-vNS-Luc, pCAGGS-PB2, pCAGGS-PA, pCAGGS-NP, and either pCAGGS-PB1-wild type or pCAGGS-PB1-K471 mutants. Cells were incubated at 31°C, 34°C, or 37°C for 24 h and were harvested and subsequently assayed for luciferase activity. Quantitative results are presented as the average with the SD from at least three independent experiments.

Next, we tested the effect of Lys471 residue substitution on viral polymerase activity by using the mini-replicon assay. We constructed PB1 expression plasmids for mammalian cells and confirmed the synthesis of these PB1-K471 mutant proteins by Western blot analysis (Fig. 3A). The RNA synthesis rates of PB1-wild-type and K471 mutant proteins are shown in Table 3 (see “relative fold change”) and Fig. 3B. Plasmid-transfected cells were incubated at 31°C, 34°C, or 37°C for 24 h and were subsequently assayed for luciferase activity. The viral polymerase activities of the mutants with PB1-K471F(Phe), K471Y(Tyr), K471G(Gly), K471S(Ser), K471N(Asn), K471D(Asp), K471E(Glu), and K471W(Trp) substitutions were reduced more than 10-fold in comparison to that of the PB1-wild type at 34°C, whereas activities were reduced by only about 10-fold in the other, viable K471 mutants excluding K471A (Fig. 3B, middle panel). The level of viral RNA synthesis from the PB1-K471A mutant polymerase was reduced to 3.7% compared to that from the PB1-wild type at 34°C; therefore, it is possible that a decrease in polymerase activity induced a marked reduction in the plaque size reduction of a K471A mutant virus (Fig. 2). On the other hand, the viral polymerase activities of 17 PB1-K471 mutants excluding K471R and K471I were reduced more than 100-fold in comparison to that of the PB1-wild type at 37°C (Fig. 3B, bottom panel). These results suggest that the reduction in viral polymerase activity by more than 100-fold compared to PB1-wild type led to the suppression of viral proliferation. Specifically, plaque formation was not observed at 37°C in those 17 PB1-K471 mutant strains (Fig. 2). The polymerase activities of all 19 PB1-K471 mutants at 31°C showed a similar trend to the results at 34°C. Overall, these data suggested that a PB1-K471 mutant can be applied to the LAIV by using a temperature-sensitive phenotype.

**TABLE 3:** Effect of substitution of Lys471 residue on viral polymerase activity

Test virus	Polymerase activity					
	at 31°C		at 34°C		at 37°C	
	Relative fold change (log_10_)^*a*^	*P^b^*	Relative fold change (log_10_)	*P*	Relative fold change (log_10_)	*P*
PB1-wild type (K471)	4.00		6.00		6.00	
PB1-K471H	3.93	3.2 × 10^−2^	5.94	4.7 × 10^−2^	3.82	5.0 × 10^−4^
PB1-K471R	3.93	2.9 × 10^−3^	5.91	7.8 × 10^−2^	5.67	5.0 × 10^−3^
PB1-K471V	3.05	5.8 × 10^−4^	5.80	6.2 × 10^−3^	1.77	5.0 × 10^−4^
PB1-K471I	3.35	7.3 × 10^−4^	5.93	2.3 × 10^−2^	3.35	5.0 × 10^−4^
PB1-K471L	3.66	7.1 × 10^−4^	5.94	1.1 × 10^−1^	3.36	5.1 × 10^−4^
PB1-K471M	3.60	3.0 × 10^−3^	6.06	7.8 × 10^−2^	3.70	5.0 × 10^−4^
PB1-K471C	2.89	4.6 × 10^−4^	5.17	2.4 × 10^−4^	2.09	5.0 × 10^−4^
PB1-K471P	2.85	4.8 × 10^−4^	5.10	5.1 × 10^−4^	1.01	5.1 × 10^−4^
PB1-K471T	3.16	2.6 × 10^−4^	5.73	2.4 × 10^−3^	2.51	5.0 × 10^−4^
PB1-K471Q	3.84	1.4 × 10^−2^	5.89	1.8 × 10^−2^	2.07	5.0 × 10^−4^
PB1-K471A	2.45	4.3 × 10^−4^	4.57	2.0 × 10^−4^	1.77	5.0 × 10^−4^
PB1-K471F	1.23	4.5 × 10^−4^	3.31	1.9 × 10^−4^	1.14	5.0 × 10^−4^
PB1-K471Y	1.08	4.5 × 10^−4^	2.91	1.9 × 10^−4^	0.97	5.0 × 10^−4^
PB1-K471G	2.52	4.1 × 10^−4^	4.03	2.0 × 10^−4^	0.86	5.0 × 10^−4^
PB1-K471S	2.68	4.7 × 10^−4^	4.56	1.9 × 10^−4^	1.36	5.0 × 10^−4^
PB1-K471N	3.17	5.0 × 10^−4^	4.66	2.6 × 10^−4^	1.12	5.0 × 10^−4^
PB1-K471D	0.51	4.5 × 10^−4^	1.97	1.9 × 10^−4^	0.27	5.0 × 10^−4^
PB1-K471E	0.98	4.4 × 10^−4^	2.49	1.9 × 10^−4^	0.53	5.0 × 10^−4^
PB1-K471W	0.40	4.5 × 10^−4^	1.71	1.9 × 10^−4^	0.39	5.0 × 10^−4^

^*a*^ The values were calculated using data from Fig. 3 (polymerase activity data).

^*b*^ *P*-values were calculated using the *t*-test, in which we compared the polymerase activity for a particular mutation with the polymerase activity obtained using the PB1-wild type.

### PB1-K471P strain maintained the temperature-sensitive phenotype after serial passages in cell culture

**Fig 4:**
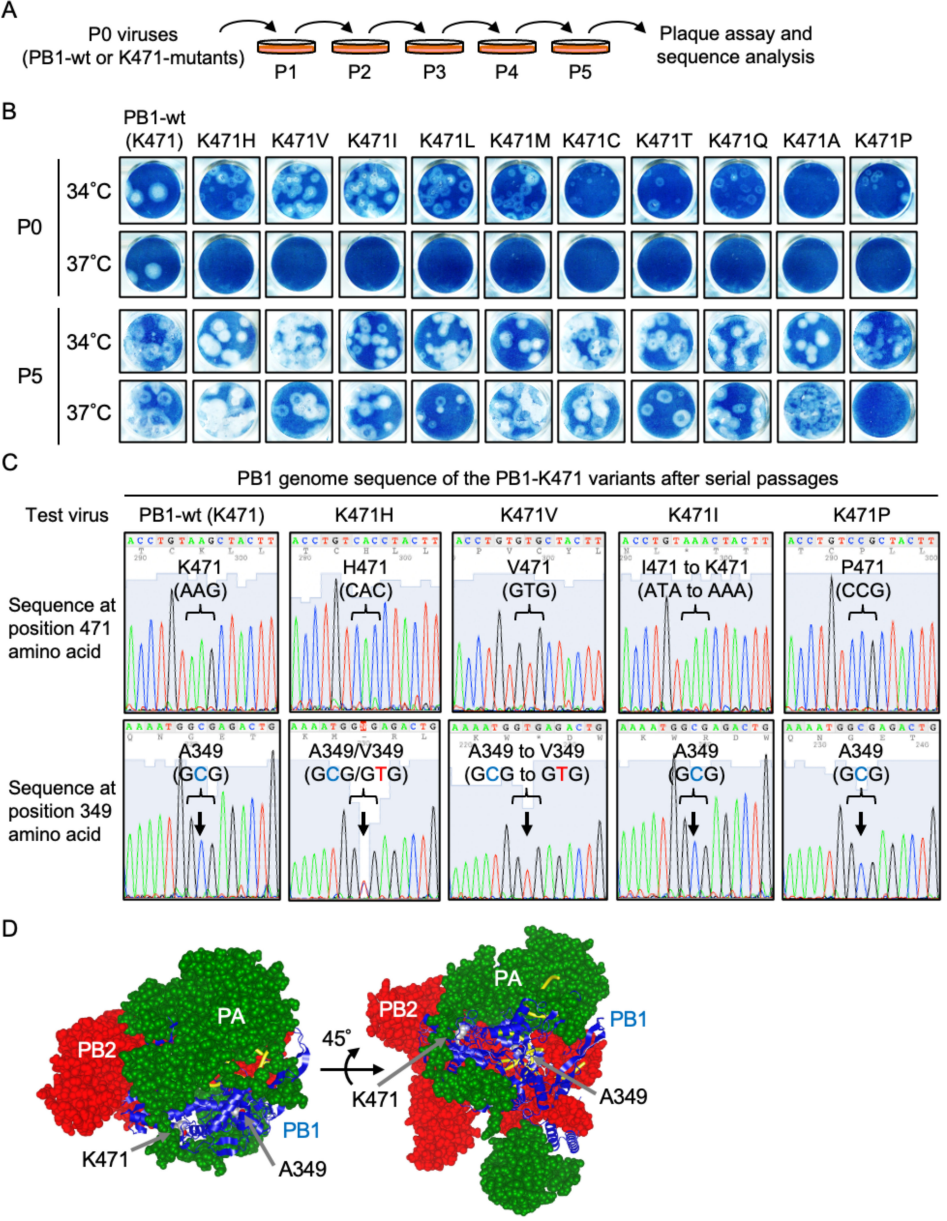
A A349V mutation in PB1 restores the temperature-sensitive phenotype of the PB1-K471 variants, excluding the PB1-K471P virus. (**A**) Diagram of virus serial passage. MDCK cell cultures were infected at an MOI of 1 with PR8-PB1-wild type and PR8-PB1-K471 variants and cultured at 34°C. From 3 dpi, the supernatant was recovered and named P1. The supernatant P1 was used for the second passage of infection, after which the cells were cultured at 34°C, and this procedure was repeated five times. Alteration of the temperature-sensitive phenotype by serial virus passage was checked using a plaque assay at 34°C or 37°C, and nucleotide and amino acid substitution was detected in the PR8-PB1-K471 variants showing loss of the temperature-sensitive phenotype. (**B**) Temperature sensitivity of PR8-PB1-K471 variants P5. MDCK cells were infected with P5 of PR8-PB1-wild type or PB1-K471 variants, and plaque assays were then carried out. Cells were infected 1 to 20 PFU per well. Cells were cultured at 34°C or 37°C for 4 or 5 days. Virus plaques were visualized by Amido Black 10B staining and photographed. (**C**) Introduction of A349V substitution occurred in a PB1-K471-variant after serial virus passages. Sequence of Lys471 and Ala349 regions in the PB1 genome after serial passage. Virion RNA was isolated, amplified by RT-PCR, and sequenced from PB1-wild type, PB1-K471H, PB1-K471V, PB1-K471I, and PB1-K471P viruses after serial passages (P5). The wild-type sequence at position 349 amino acid codon is GCG (Ala residue), and the A349V sequence is GTG (Val residue). A mixture of GCG and GTG, or GTG, was detected at position 349 residue in PB1-K471H or PB1-K471V viruses. In PB1-K471I virus, the Ile471 residue changed the Lys471 residue after serial viral passages. In PB1-K471P virus, the Pro471 and the Ala349 residues were maintained after serial viral passages. (**D**) Position of Ala349 and Lys471 in the PB1 subunit in a heterotrimeric polymerase complex. PB2 (red) and PA (green) or PB1 (blue) subunits are shown in a surface representation or ribbon diagram structure, respectively. The vRNA duplex chain is represented in yellow.

The live-attenuated vaccine should not revert back to the wild-type virus or a revertant virus following vaccination. Therefore, the LAIV strain needs an attenuated phenotype involved in temperature sensitivity during viral growth, including during the vaccine manufacture processes. To apply PB1-K471-variant to LAIV, we tested whether these mutants could maintain a temperature-sensitive phenotype on temperature-sensitive PB1-K471 mutants using a mammalian cell culture (Fig. 4A). MDCK cells were infected with PB1-wild type and PB1-K471 variants and cultured at 34°C. From 3 days post-infection (dpi), the supernatant was recovered and named passage 1 (P1). The virus passages were then continued until P5, and temperature sensitivity of the P0 and P5 viruses was checked at 34°C and 37°C. As shown in Fig. 4B, the P0 virus of 10 PB1-K471 variants did not form plaques at 37°C (shown by Amido Black 10B staining), and in addition, PB1-K471C and PB1-K471A did not display clear plaque formation at 34°C. According to the two independent experiments of virus passages, PB1-K471 variants (except for PB1-K471P) overcame the growth restriction on 37°C incubation at P5. In addition, P5 viruses of PB1-K471 variants formed clearly visible plaques at 34°C, including PB1-K471P. These results suggested that only the PB1-K471P-variant maintained temperature-sensitive phenotypes that prevented the reversal of growth restrictions at 37°C, after serial passages.

Next, we identified the sequence of the viral genomes derived from the P5 viruses of PB1-K471 variants that adapted to the viral growth at 37°C. We analyzed the nucleotide sequences of all three polymerase subunits (PB1, PB2, and PA)-specific and NP-specific reverse-transcription PCR products generated using RNA collected from P5 viruses. The observed nucleotide sequence and amino acid changes are summarized in Fig. 4C and Table 4. We found that the PB1-K471P-variant maintained Pro residue at position 471 in PB1 after serial viral passages. On the other hand, PB1-K471I, PB1-K471M, or PB1-K471Q variants changed to Lys residues (AAG or AAA codon) from the Ile (ATA codon), Met (ATG codon), or Gln (CAG codon) residue. P5 viruses of PB1-K471I, PB1-K471M, or PB1-K471Q variants lost the temperature-sensitive phenotype, due to being reverted to PB1-wild-type viruses, as a result of viral passages. In other PB1-K471 variants (K471H, K471V, K471L, K471C, K471T, and K471A), the position 471 amino acid did not revert to a Lys residue, whereas one additional mutation into the PB1 subunit, which resulted in an Ala-to-Val change at position 349, was identified. This amino acid change was caused by a C-to-T transition at the second base of codon 349 (GCG to GTG). We assume that the mutation of PB1-A349V induced a structural change in PB1, canceling the ability of K471-mutation involving temperature sensitivity. A349V was localized close to the active site, near the amino acid at position 471 (Fig. 4D).

**TABLE 4:** Ala-to-Val change at position 349 in a serial passaged PB1-K471 mutants

Test virus	Codon of 471 amino acid		Codon of 349 amino acid	
	Before passage^*a*^	After passage^*b*^	Before passage^*c*^	After passage^*d*^
PB1-wild type (K471)	AAG (Lys)	**AAG**	GCG (Ala)	GCG
PB1-K471H	CAC (His)	CAC	GCG	GCG/**GTG** (Ala/Val)
PB1-K471V	GTG (Val)	GTG	GCG	**GTG** (Val)
PB1-K471I	ATA (Ile)	**AAA** (Lys)	GCG	GCG
PB1-K471L	CTG (Leu)	CTG	GCG	**GTG** (Val)
PB1-K471M	ATG (Met)	**AAG** (Lys)	GCG	GCG
PB1-K471C	TGT (Cys)	TGT	GCG	GCG/**GTG** (Ala/Val)
PB1-K471T	ACG (Thr)	ACG	GCG	GCG/**GTG** (Ala/Val)
PB1-K471Q	CAG (Gln)	**AAG** (Lys)	GCG	GCG
PB1-K471A	GCG (Ala)	GCG	GCG	**GTG** (Val)
PB1-K471P	CCG (Pro)	CCG	GCG	GCG

^*a*^ Codon at position 471 in each PB1-K471 mutant viruses before serial passage.

^*b*^ PB1-K471I, PB1-K471M, and PB1-K471Q viruses, AAA (Lys residue) or AAG (Lys residue) codon at position 471 was detected predominantly after serial passage. Bold indicates AAG and AAA codon.

^*c*^ PB1-wild type and PB1-K471 mutants sequence at position 349 is GCG (Ala residue) before serial passage.

^*d*^ In PB1-K471V, PB1-K471L and PB1-K471A viruses, a GTG (Val residue) codon at position 349 was detected predominantly after serial passage. In PB1-K471H, PB1-K471C, and PB1-K471T viruses, a mixture of GCG and GTG was detected at this position. Bold indicates the GTG codon.

**Fig 5:**
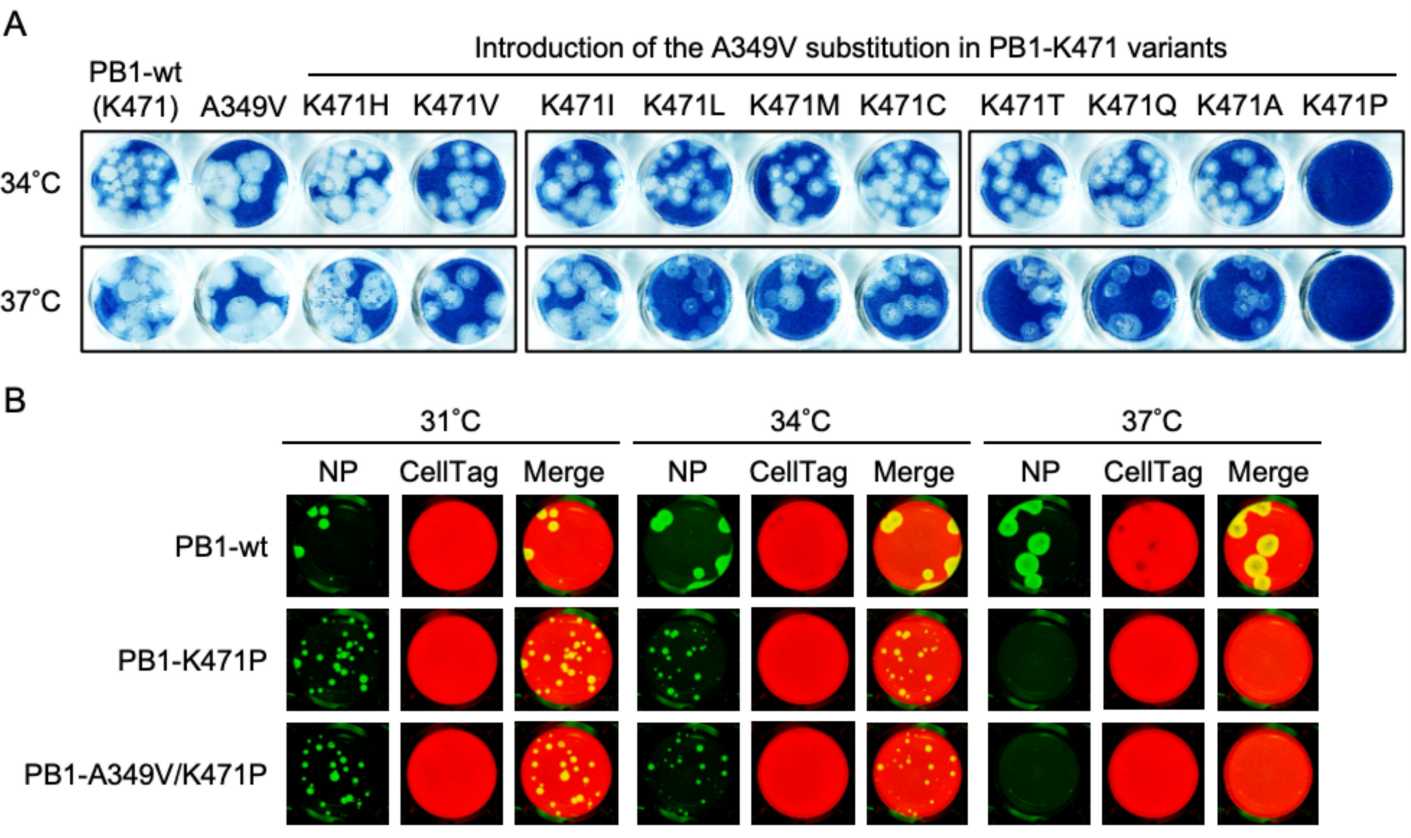
The A349V second substitution in the PB1-K471P variant did not induce reversion to a wild-type phenotype involving cancelation of temperature sensitivity. (**A and B**) Plaque assay was performed by using an amplified version of each virus in chicken eggs. Plaque assay of PB1-wild type, PB1-A349V, or PB1-A349V/K471 mutant viruses in MDCK cells. Cells were infected 1 to 20 PFU per well. Cells were incubated at 34°C or 37°C for 4 days and then stained with Amido Black 10B (**A**). Plaque assay of PB1-wild type, PB1-K471P, or PB1-A349V/K471P viruses in MDCK cells. The PB1-wild type or PB1 mutant viruses were used to infect MDCK cells at the indicated temperatures. MDCK cells were infected with the viruses indicated at the left of the figure and incubated at 31°C, 34°C, or 37°C for 3 days. Virus plaques were visualized by immunostaining and photographed (**B**).

To examine the effect of the PB1-A349V substitution into the PB1-K471 variants on temperature sensitivity, we generated a PB1-A349V and a PB1-A349V/K471-double mutant virus and then propagated the mutant viruses in chicken eggs, examined by HA assay and plaque assay. HA titers of PB1-A349V and PB1-A349V/K471-double mutants were 1,024 to 2,048 HA units (Table 5, “HA titer” column). The PB1-A349V virus formed plaques similar to that of PB1-wild type at 34°C or 37°C (Fig. 5A). The PB1-A349V single mutation had no influence on the induction of temperature sensitivity. PB1-A349V/K471-double mutants (except PB1-A349V/K471P) had the ability of plaque formation at 34°C and 37°C for cultures of 4 days. This result suggests that A349V substitution on PB1 restored the ability of viral proliferation at 37°C for PB1-K471 variants, except for PB1-K471P. To determine the virus titer and temperature sensitivity of PB1-A349V/K471P-variant, plaque assays were carried out, and the infected cells were cultured for 3 days at 31°C, 34°C, or 37°C (Fig. 5B and Table 6). The PB1-A349V/K471P-variant formed clearly visible plaques at 31°C or 34°C by immunostaining. Therefore, the PB1-K471P-variant could maintain a temperature-sensitive phenotype, even if the A349V second substitution was induced in PB1. To strengthen the result that the single K471P amino acid change in the PB1 polymerase of the PR8 strain had induced genetic stability, we conducted an additional serial virus passage experiment, and its data are described in the supplemental material (Fig. S1 and Table S1). According to a previous study (19), we performed serial virus passages in MDCK cells at gradually elevated temperatures (Fig. S1A). The results of these experiments suggested that the PR8-PB1-K471P strain could not produce progeny viruses during the 11 passages under increasing temperatures.

**TABLE 5:** Growth kinetics of PR8-PB1-A349V and PR8-PB1-A349V/K471 variants in eggs

Test virus	HA titer^*a*^	PFU/mL of E1 viruses^*b*^ at	
		34°C	37°C
PB1-A349V	1,024	2.3 ± 0.3 × 10^8^	1.3 ± 0.2 × 10^8^
PB1-A349V/K471H	2,048	2.6 ± 0.2 × 10^8^	8.0 ± 3.6 × 10^7^
PB1-A349V/K471V	2,048	1.9 ± 0.2 × 10^8^	7.3 ± 3.3 × 10^7^
PB1-A349V/K471I	2,048	1.8 ± 0.2 × 10^8^	1.5 ± 0.1 × 10^8^
PB1-A349V/K471L	2,048	3.2 ± 0.4 × 10^8^	1.1 ± 0.3 × 10^8^
PB1-A349V/K471M	2,048	2.2 ± 0.2 × 10^8^	8.3 ± 1.2 × 10^7^
PB1-A349V/K471C	2,048	3.0 ± 0.6 × 10^8^	9.3 ± 1.2 × 10^7^
PB1-A349V/K471T	2,048	1.7 ± 0.09 × 10^8^	6.3 ± 0.9 × 10^7^
PB1-A349V/K471Q	2,048	2.5 ± 0.3 × 10^8^	6.3 ± 0.3 × 10^7^
PB1-A349V/K471A	2,048	2.5 ± 0.5 × 10^8^	7.0 ± 1.1 × 10^7^
PB1-A349V/K471P	1,024	NC^*c*^	ND^*d*^

^*a*^ HA titers of viruses isolated from eggs were determined by HA assay.

^*b*^ PFU/mL of viruses isolated from eggs were determined by plaque assay. The number of plaques was counted following Amido Black 10B staining after 4 days of inoculation at 34°C or 37°C. Data are means from three independent experiments. Errors are represented as standard error.

^*c*^ NC, not calculated. PB1-A349V/K471P could not form a clear viral plaque using by Amido Black staining at 34°C for 4 days incubation.

^*d*^ ND, not detected. ND means that no plaque formation was observed in a plaque assay using 1 mL of undiluted virus stock.

**TABLE 6:** Growth kinetics of PR8-PB1-A349V/K471P in eggs

Test virus	HA titer^*a*^	PFU/mL of E1 viruses at^*b*^		
		31°C	34°C	37°C
PB1-wild type	2,048	6.0 ± 1.3 × 10^8^	5.5 ± 1.0 × 10^8^	5.2 ± 2.0 × 10^8^
PB1-K471P	1,024	2.6 ± 0.1 × 10^8^	9.0 ± 1.1 × 10^7^	ND^*c*^
PB1-A349V/K471P	1,024	1.4 ± 0.2 × 10^7^	2.2 ± 0.1 × 10^6^	ND

^*a*^ HA titers of viruses isolated from eggs were determined by HA assay.

^*b*^ PFU/mL of viruses isolated from eggs were determined by plaque assay. The number of plaques was counted following immunostaining after 3 days of inoculation at 31°C, 34°C, or 37°C. Data are means from four independent experiments. Errors are represented as standard error.

^*c*^ ND, not detected. ND means that no plaque formation was observed in a plaque assay using 1 mL of undiluted virus stock.

### A single dose of PB1-K471-variant can elicit protection against influenza virus challenges in animals

**Fig 6:**
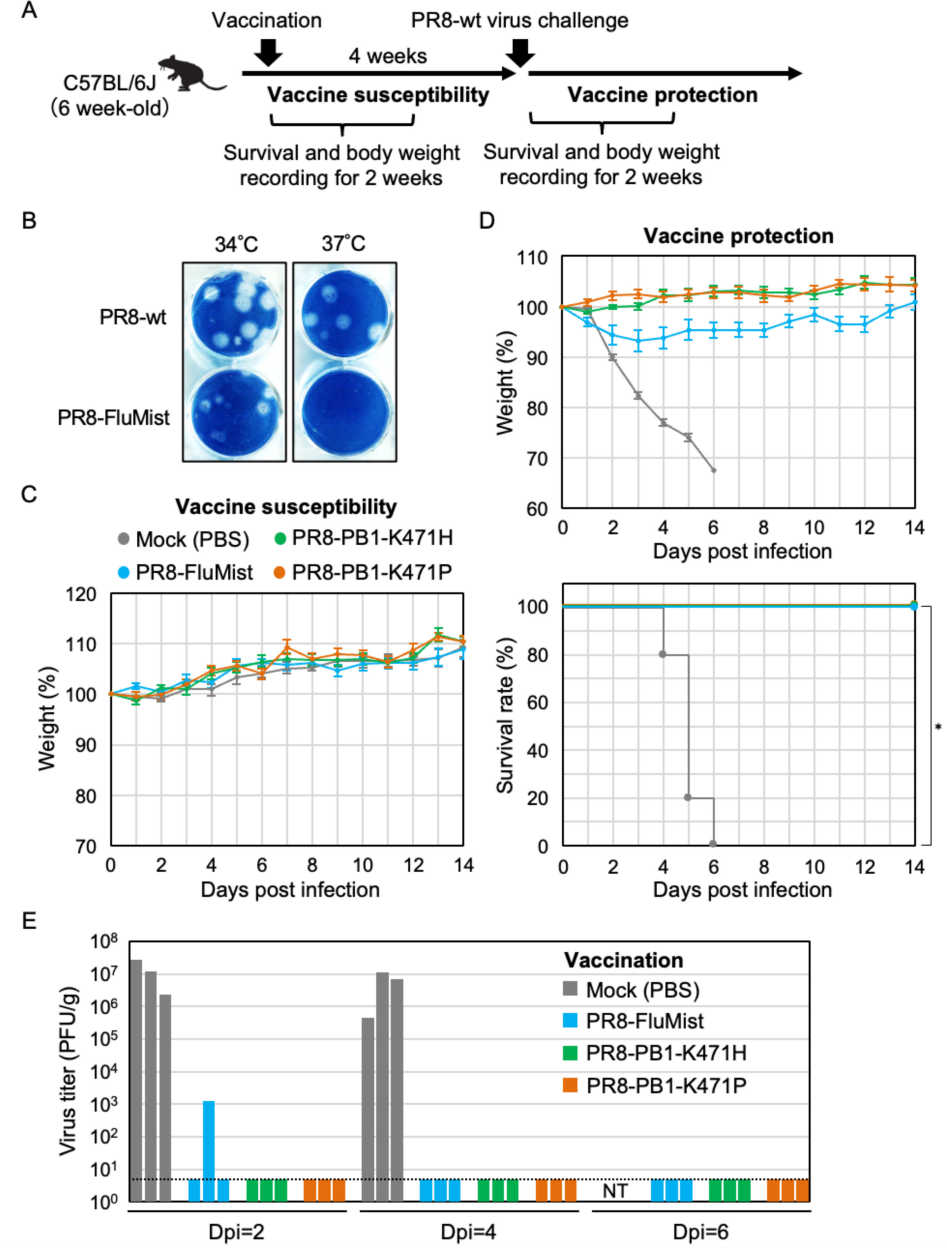
Protection against influenza wild-type virus after vaccination using PB1-K471H or PB1-K471P as a live-attenuated vaccine strain. (**A**) Experimental scheme of PR8-PB1-K471-variant immunization and wild-type virus challenge. Mouse weight and survival were measured at the indicated days after vaccination and then following the wild-type virus challenge. (**B**) Temperature-sensitive phenotype of the PR8-Flumist strain. Plaque morphology of PR8-wild type and PR8-FluMist strains. A plaque assay was performed using an amplified version of each virus in chicken eggs. MDCK cells were infected with the viruses indicated at the left of the figure and incubated at 34°C or 37°C for 4 or 5 days. Virus plaques were visualized by Amido Black 10B staining and photographed. (**C and D**) A single immunization with PR8-PB1-K471 variants vaccine protects mice from a lethal viral challenge. Six-week-old female C57BL/6J mice were intranasally inoculated with 10^6^ PFU of PR8-PB1-K471H (*n* = 9), PR8-PB1-K471P (*n* = 9), or PR8-FluMist (*n* = 9) virus, and PBS (Mock, *n* = 10). Body weight was monitored for 14 days after vaccination (**C**). Four weeks after vaccination, the mice were intranasally challenged with 10^7^ PFU of PR8-wild-type virus. Body weight (top) and survival (bottom) were monitored for 14 days after the challenge (**D**). The body weights of animals inoculated with viruses are depicted as a percentage of the body weight compared with that on day 0. Data are mean body weight ± SEM. Statistical significance was analyzed by log-rank test (*, *P* = 1.5 × 10^−5^). (**E**) Virus titer in the lung homogenate of PR8-PB1-K471H-, PR8-PB1-K471P- or PR8-FluMist-vaccinated mice at 2, 4, and 6 dpi following an intranasal challenge with PR8-wild-type virus (*n* = 3 mice/group). The dotted lines indicate the limit of detection. NT: Not tested.

To assess the potential of the PR8-PB1-K471-variant for the LAIV strain, we examined whether vaccination with a single dose of PR8-PB1-K471H or PR8-PB1-K471P viruses could protect mice against a lethal influenza virus. Firstly, we tested vaccine susceptibility in animals: C57BL/6J mice were infected intranasally with 10^6^ PFU of PR8-PB1-K471H or PR8-PB1-K471P viruses and monitored for body weight and survival rate (Fig. 6A). In addition, to evaluate the vaccination of PR8-PB1-K471 variants, a PR8-based temperature-sensitive strain was created, designated PR8-FluMist (see Materials and Methods, PR8-FluMist strain encoding PB1-K391E/E581G/A661T & PB2-N265S) (Fig. 6B); the licensed LAIV FluMist backbone. The PR8-FluMist strain did not show plaque formation by Amido Black 10B staining at 37°C (Fig. 6B) and that was used as a control vaccine strain for comparison with the PR8-PB1-K471H- or the PR8-PB1-K471P-vaccinated mice. Previously, it has been reported that introducing five amino acid mutations (PB1-K391E/E581G/A661T, PB2/N265S, and NP/D34G) into PR8 virus induced a temperature-sensitive phenotype (20). In this study, we generated recombinant influenza strains using egg-adapted high-growth PR8 virus provided by Dr. Yoshihiro Kawaoka (University of Wisconsin-Madison). Since the amino acid position 34 of the NP protein of the egg-adapted high-growth PR8 virus is glycine residue, therefore, we introduced four amino acid mutations in PB1 (K391E, E581G, and A661T) and PB2 (N265S) to create a temperature-sensitive strain based on PR8. Likewise, a group has reported that the NP-D34G mutation in FluMist is already present in a PR8 strain (21, 22). The results of viral growth kinetics and plaque assays of PR8-FluMist and PR8-PB1-K471-variant at various temperatures were described in the supplemental material (Fig. S2 and S3).

The PR8-PB1-K471 variants- or PR8-FluMist-vaccinated mice maintained a normal weight and survived as well as the mock-vaccinated group (Fig. 6C). To confirm the pathogenicity of the viral dose of 10^6^ PFU, which is the same dose used for PR8-FluMist or PR8-PB1-K471 variants vaccination, PR8-wild-type virus was intranasally inoculated into mice at a dose of 10^6^ PFU (Fig. S4). Mice infected with 10^6^ PFU of PR8-wild-type virus showed severe symptoms accompanied by rapid weight loss, and subsequently, three out of eight mice died after infection. This indicates that the PR8-PB1-K471 variants are attenuated and have potential for use as a safe attenuated live vaccine strain. Four weeks after intranasal inoculation with PR8-PB1-K471 variants or PR8-FluMist, mice were given a lethal dose of 10^7^ PFU of PR8-wild-type virus (Fig. 6D). Mock-vaccinated mice infected with PR8-wild-type virus developed severe symptoms with rapid weight loss and did not survive for more than 6 days after infection. On the other hand, PR8-PB1-K471H- or PR8-PB1-K471P-vaccinated mice maintained body weight and survived for 14 days. Similarly, PR8-FluMist-vaccinated mice also survived following infection, however, slight weight loss was observed until 3 dpi.

Next, we assessed the control of virus titers in infected mice mediated by these vaccine candidates (Fig. 6E). We infected vaccinated mice with PR8-wild-type virus and harvested lungs at 2, 4, or 6 dpi and measured viral titers in the lung homogenates. In PR8-PB1-K471H- or PR8-PB1-K471P-vaccinated mice, the challenge virus was below the level of detection, whereas titers of 10^5^ to 10^7^ PFU/g were found in lungs of mock-vaccinated mice at 2 or 4 dpi. Mice vaccinated with PR8-FluMist had reduced virus titers following challenge with PR8-wild type at 2 dpi, and no challenge virus was detected at 4 or 6 dpi. These results suggested that PR8-PB1-K471 variants have the strong immunizing potential for the influenza virus.

### Vaccination with PR8-PB1-K471P elicits antibody responses and increases influenza-specific IFN-γ-secreting T cells

**Fig 7:**
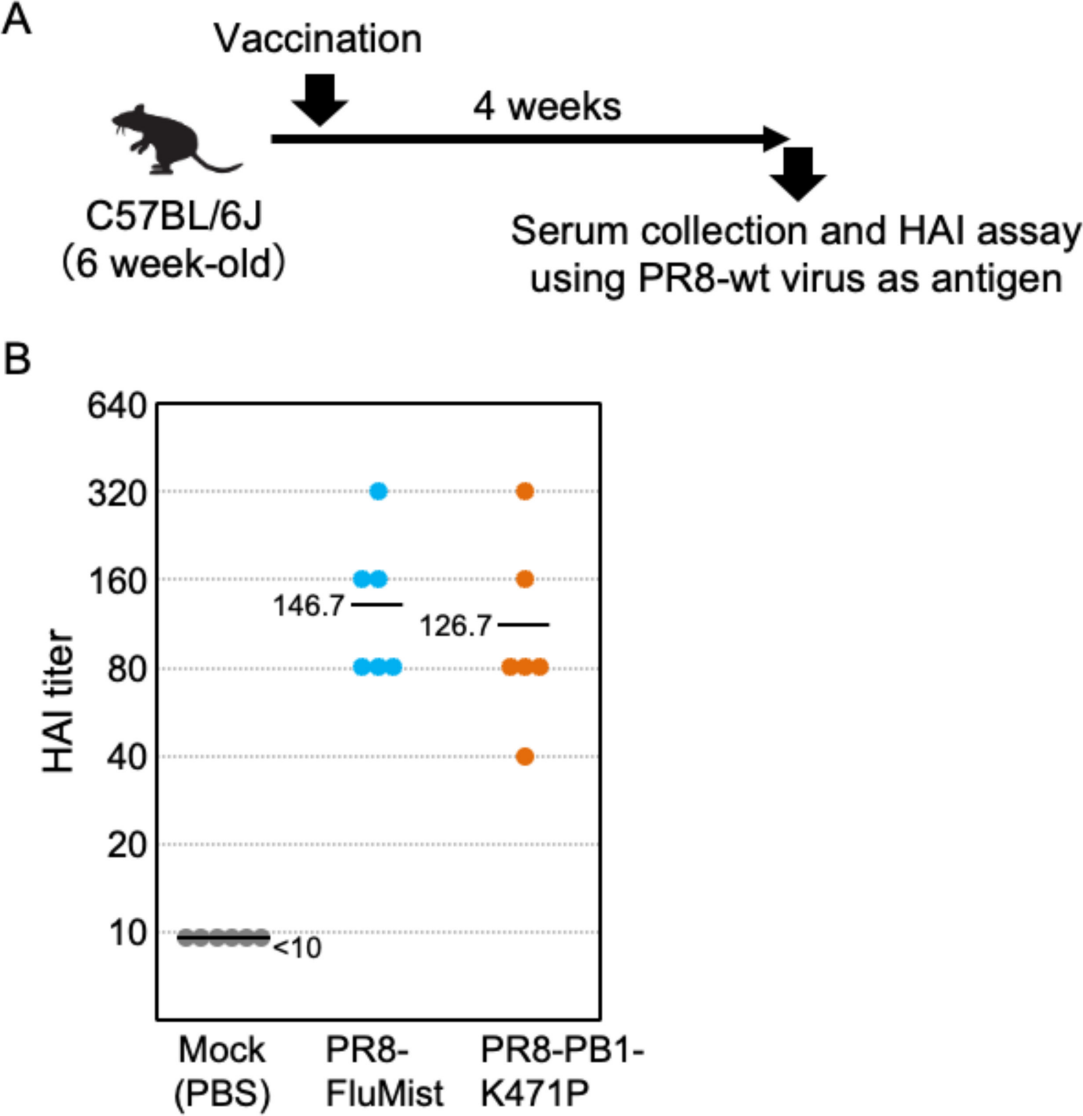
HA inhibition antibody titers of mice post-vaccination with PR8-PB1-K471P. (**A**) Experimental scheme of PR8-PB1-K471P immunization and HAI assay. (**B**) HAI assay of vaccinated and unvaccinated mouse serum against PR8-wild type (PB1-wt) virus. Six-week-old female C57BL/6J mice were intranasally inoculated with 10^6^ PFU of PR8-PB1-K471P (*n* = 6), or PR8-FluMist (*n* = 6) virus, and PBS (Mock, *n* = 6). Four weeks after vaccination, sera were collected, and HAI titers against PR8-wild-type-virus were measured by HAI assay.

**Fig 8:**
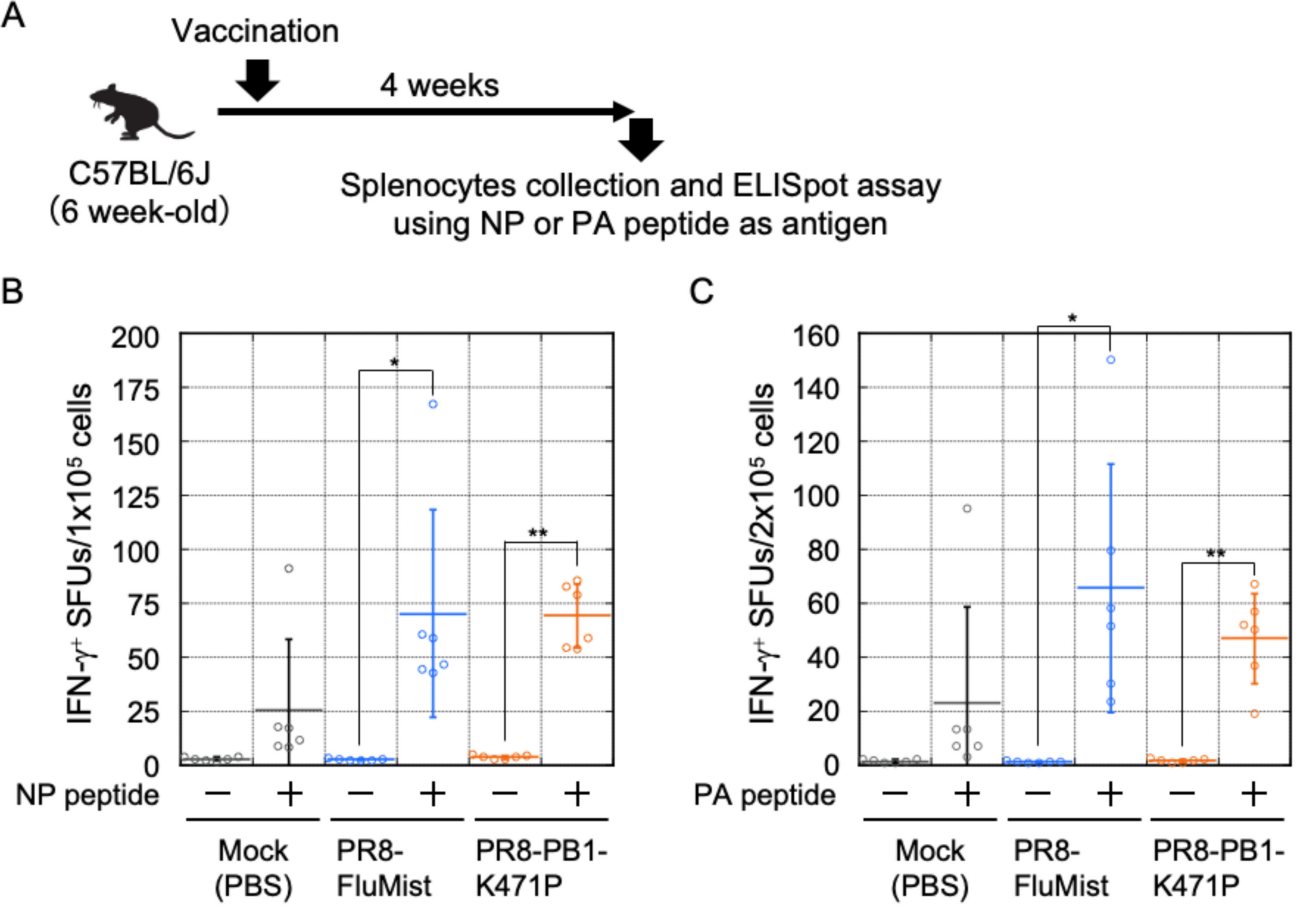
PR8-PB1-K471P-vaccinated mice showed increases in cell-mediated immune response. (**A**) Experimental scheme of PR8-PB1-K471P immunization and ELISpot assay. (**B and C**) Six-week-old female C57BL/6J mice were intranasally inoculated with 10^6^ PFU of PR8-PB1-K471P (*n* = 6), or PR8-FluMist (*n* = 6) virus, and PBS (Mock, *n* = 6). Four weeks after vaccination, the splenocytes were obtained from those mice. IFN-γ^+^ SFUs were enumerated via ELISpot assays after stimulating splenocytes with NP_366-374_ (**B**) or PA_224-233_ (**C**). Significance was determined using Student’s t test: (**B**) *, *P* = 1.8 × 10^-2^; **, *P* = 1.3 × 10^-4^; (**C**) *, *P* = 1.9 × 10^-2^; **, *P* = 1.1 × 10^-4^.

The host response to vaccination with PR8-PB1-K471P suggested strong protective efficacy against viral infection, including adaptive immunity. We tested antibody responses by vaccinating groups of six mice with 10^6^ PFU of PR8-FluMist or PR8-PB1-K471P and collected serum at 4 weeks post-inoculation. The antibody response was determined by a hemagglutination inhibition (HAI) assay. The PR8-PB1-K471P virus was capable of inducing an equally high HAI titer as the PR8-FluMist (Fig. 7). Furthermore, to confirm whether PR8-PB1-K471P vaccination induces T cell responses, we measured the production of interferon-gamma (IFN-γ) in immunized mice. Splenocytes were collected from vaccinated mice (*n* = 6) at 4 weeks post-inoculation and stimulated with the MHC class I pentamers, NP_366–374_ or PA_224–233_ peptides, and subjected to the ELISpot assay to evaluate the CD8^+^ cytotoxic T-cell activity. As shown in Fig. 8, significantly higher IFN-γ spot-forming units (SFUs) were observed in the antigen peptides-stimulated PR8-PB1-K471P-vaccinated group than in the unstimulated mice group (Fig. 8B and C). PR8-FluMist vaccination also was significantly induced IFN-γ production by stimulation of antigen peptides, and the degree of IFN-γ^+^ SFUs was similar between the PR8-PB1-K471P-vaccinated group and the PR8-FluMist-vaccinated group.

**Fig 9:**
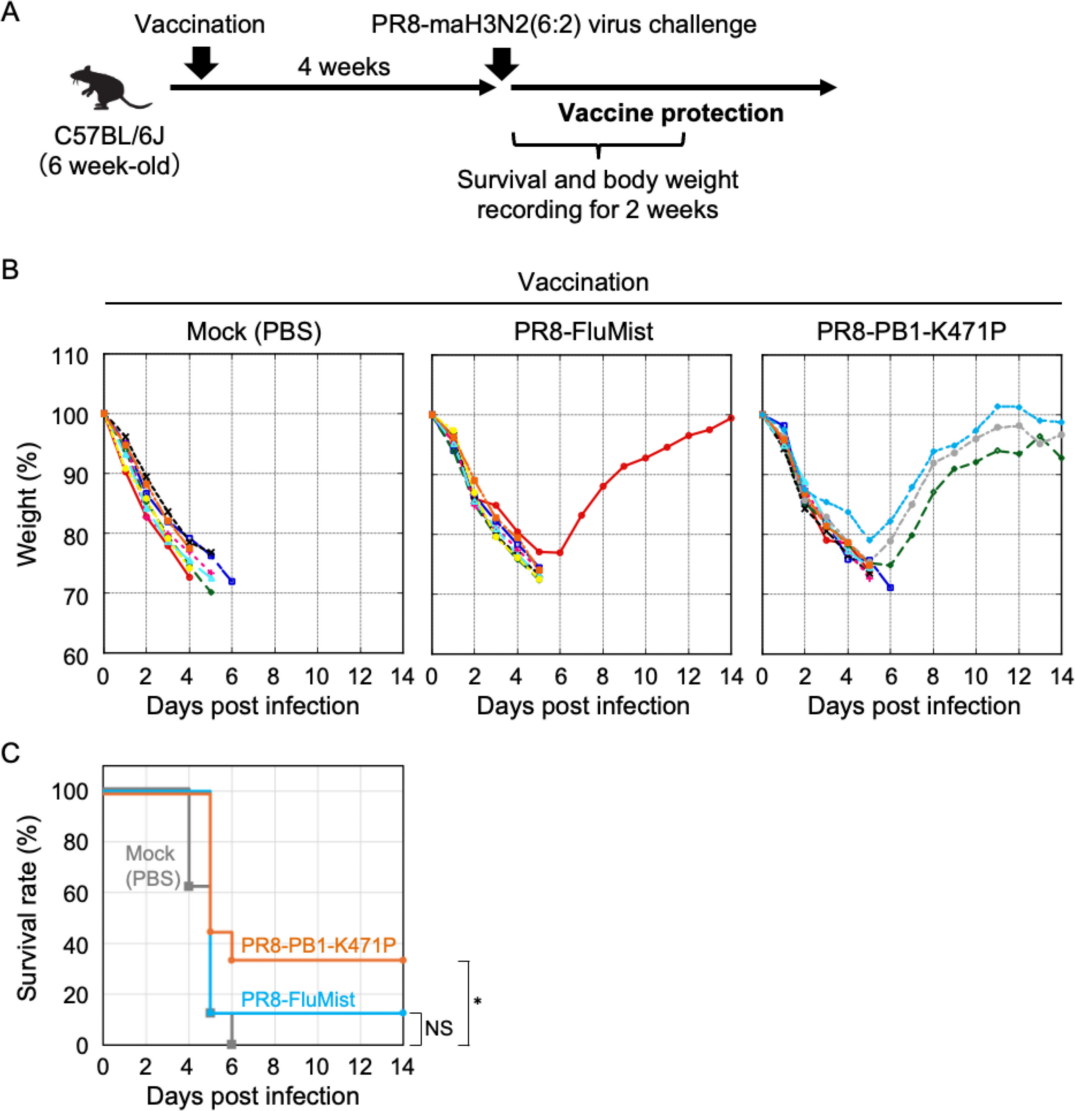
Protection against a recombinant variant coding mouse-adapted HA and NA derived from H3N2 virus after vaccination using PR8-PB1-K471P as a live-attenuated vaccine strain. (**A**) Experimental scheme of PR8-PB1-K471P immunization and PR8-maH3N2(6:2) virus challenge. (**B and C**) A single immunization with PR8-PB1-K471P vaccine protects mice from a lethal virus challenge. Six-week-old female C57BL/6J mice were intranasally inoculated with 10^6^ PFU of PR8-PB1-K471P (*n* = 9), or PR8-FluMist (*n* = 8) virus, and PBS (Mock, *n* = 8). Four weeks after vaccination, the mice were intranasally challenged with 10^6^ PFU of PR8-maH3N2(6:2) virus. Body weight (**B**) and survival (**C**) were monitored for 14 days after the challenge. The body weights of animals inoculated with viruses are depicted as a percentage of the body weight compared with that on day 0. Statistical significance was analyzed by log-rank test (*, *P* = 1.9 × 10^−2^; NS, not-significant).

Live-attenuated vaccines are thought to give broad cross-protection against heterotypic viruses through activation of both humoral and cell-mediated immune response. To confirm the protective effect of PR8-PB1-K471P against challenge infection with heterologous strain, we generated PR8-maH3N2(6:2) virus expressing the HA and NA of A/Hong Kong/MA(mouse-adapted)/1968/H3N2 strain on the PR8 backbone. To evaluate the protection from disease or death, we challenged PR8-PB1-K471P or PR8-FluMist-vaccinated mice with 10^6^ PFU of PR8-maH3N2(6:2) viruses (Fig. 9A). All of the mock-vaccinated mice died (*n* = 8), but three out of nine mice vaccinated with the PR8-PB1-K471P survived (Fig. 9B). The PR8-PB1-K471P-vaccinated group showed 30% survival despite body weight loss (*P* = 1.9 × 10^−2^ by the log-rank test), whereas the group at PR8-FluMist inoculation survived one out of eight mice (Fig. 9C). Collectively, these results indicated that the administration of PR8-PB1-K471P as a live-attenuated vaccine induces protective effects against influenza virus in mice and promotes virus clearance.

## DISCUSSION

In this study, we analyzed the properties of recombinant viruses in which mutations were introduced into the Lys471 residue of PB1 in the influenza virus RNA polymerase. Of the 19 variants, 10 were viable and those PB1-L471 mutant viruses acquired temperature sensitivity. Nine PB1-K471 mutants, except for the PB1-K471P virus, mutated, causing a reversion of the temperature-sensitive phenotype to wild-type, which occurred by viral serial passaging experiments using MDCK cells. The PB1-K471P variant did not revert to the wild-type PB1 phenotype even after repeated viral passages and maintained low-temperature proliferation; therefore, this strain has a high genetic stability and was suggested to be useful as the mother virus for LAIV. Results of the virus challenge experiments using animals showed that mice vaccinated with the PB1-K471P mutant strain were protected from lethal doses of wild-type virus infection. These results suggest the possibility that the single amino acid mutation of PB1 in the PR8 H1N1 strain of influenza A virus can be utilized as a backbone strain for LAIV.

Multiple temperature-sensitive mutations have been identified in the polymerase subunit of the influenza virus (18, 23–25), and several studies have reported on the determination of corresponding phenotypes (26, 27). Temperature-sensitive mutations can be broadly classified into two categories: mutations that produce thermally unstable proteins and cause defects in protein synthesis, folding, or assembly (28). The conserved Lys471 residue in PB1 is positioned in polymerase motif D of the palm domain and located near the NTP entrance tunnel. The motif D contains highly conserved lysine residues 480 and 481, which are involved in NTP binding, and is stabilized by contact with the PA helix α20 and the amino acid residues 671 to 684 of PA (29, 30). There is a possibility that mutation of PB1-Lys471 in motif D modulates correct assembly or stabilization of the heterotrimer viral polymerase comprising subunits PB1, PB2, and PA at restrictive temperature. Our results suggest that although position 349 residue in PB1 may seem distant from the RdRp active-site residues, the fingers motif and/or other polymerase motifs might undergo substantial rearrangement during polymerization by PB1-A349V substitution to cancellation of the temperature-sensitive phenotype induced in PB1-K471 mutants (except PB1-K471P variant). An amino acid residue in proteins is replaced by a proline residue that led to disturb potential structuration of those domains. Therefore, we assumed that the PR8-PB1-K471P variant did not revert to a non-temperature-sensitive phenotype even with the introduction of additional PB1-A349V substitution.

The ability of influenza viruses to aid in immune escape requires that vaccine strains need to be annually updated to reflect changes in antigenicity in the HA and the NA genes within the epidemic seasonal strains. Multiple types of influenza vaccines are currently used, containing an inactivated vaccine and LAIV of a cold-adapted virus, FluMist, delivered as an intranasal administration. LAIV is more immunogenic than inactivated vaccines, due to its ability to stimulate both humoral and cell-mediated immune responses. On the other hand, it has been reported that FluMist might not be sufficiently effective in preventing influenza virus infection in recent seasons (31). Numerous groups have reported the development of LAIV (11, 32–34), and clinical studies are underway to commercialize those vaccine candidates; however, it has not been sufficiently investigated whether those LAIV strains have genetic stability. In contrast, the importance of a live-attenuated vaccine with genetic and phenotypic stability has been demonstrated in the development and the clinical trial of oral poliovirus vaccine candidates (35–40). The PR8-PB1-K471P mutant strain isolated by our work was found to possess temperature sensitivity and genetic stability, resulting in an attenuated phenotype *in vivo*, which is an essential function of LAIV. The ability to eliminate the possibility of the emergence of pathogenic revertant mutants makes the LAIV constructed using the PR8-PB1-K471P strain useful. The PR8 strain has a very high growth efficiency in MDCK cells and chicken eggs; therefore, PR8-based inactivated vaccines have been developed encoding the HA and the NA of the seasonal influenza virus, or H5N1 and H7N9 avian influenza viruses (41, 42).

Highly pathogenic avian influenza A viruses cause severe infections in humans, and various forms of vaccines, including inactivated vaccines, are being developed (43, 44). The viral antigen of H5N1 in the inactivated vaccine can activate an effective immune response, and live-attenuated vaccines against a highly pathogenic influenza virus are being developed using A/AA/6/60 as the mother strain. These studies have advanced to the stage of clinical trial using healthy donors (45). In order to develop subtype-specific and multiple pre-pandemic vaccines, it is essential to isolate cold-adapted backbone strains for LAIV other than A/AA/6/60, and PR8-PB1-K471P could expect to be applied as one of the candidates. PR8-PB1-K471P has the potential to sufficiently meet the virus titer required for the vaccine production process. To investigate this, we produced recombinant PR8-PB1-K471P vaccines encoding the HA and the NA of seasonal influenza virus and intend to examine its efficacy in a later study (see supplemental text, Fig. S5, and Table S2). Likewise, we will attempt to develop a live-attenuated pre-pandemic vaccine encoding the antigen of a highly pathogenic influenza virus, using PR8-PB1-K471P mother strain.

Vaccine safety is determined by the characteristics of the vaccine form and the age or health condition of the host. For instance, current LAIV is restricted to healthy nonpregnant persons over 2 and under 50 years of age. Therefore, it is important to develop a novel LAIV with better safety properties to target people that do not fit the requirements for the administration of the current LAIV. In our study, PR8-PB1-K471P-vaccinated mice were fully protected against a lethal challenge with PR8-wild-type virus and did not show body weight losses. Both PR8-PB1-K471P and PR8-FluMist are temperature-sensitive viruses, but their phenotype involving the viral replication activity, etc., is not identical. This is important, as LAIV might have affected the difference in body weight between PR8-PB1-K471 variant- and PR8-FluMist-vaccinated mice after a wild-type virus challenge (Fig. 6D). We are planning additional investigations to assess the ability of PR8-PB1-K471 variants as new vaccine backbone strains to elicit protection against the infection of several subtypes of influenza virus, so the immune response can be compared with administration of licensed LAIV. Moreover, introducing the PB1-K471P additional mutation into the PB1 genome of FluMist strain may improve the genetic stability of the FluMist, which could enhance its effectiveness and safety. These studies would facilitate the clinical development of a highly safe and effective LAIV.

## MATERIALS AND METHODS

### Molecular modeling

Ribbon diagrams and space-filling representations of influenza virus polymerase were generated using structural data (PDB ID: 6T0V) and MolFeat software version 5.2.4.29 (FiatLux).

### Cells and animals

293T and MDCK cells were maintained in DMEM (Thermo Fisher Scientific) containing 10% fetal calf serum (Nichirei Biosciences) and penicillin-streptomycin in an incubator at 37°C with 5% CO_2_. C57BL/6J mice were purchased from Japan SLC. The mice were maintained at macroenvironmental temperature and humidity ranges of 20°C to 25°C and 40% to 60%, respectively, with a 12 light/12 dark light cycle.

### Generation of protein expression plasmids for PB1 mutants

Mutations corresponding to an amino acid substitution at the Lys471 (codon, AAG) residue of the PB1 subunit were introduced into a plasmid containing a sequence encoding the wild-type PB1 by site-directed mutagenesis. The Lys residue in the PB1 gene of the egg-adapted high-growth PR8 strain was changed from AAG to CAC (His codon) or AGA (Arg codon) by PCR-induced mutagenesis. To construct a plasmid containing the PB1-K471H coding sequence, two DNA fragments corresponding to the PB1 coding sequence were amplified by PCR using primers PB1-for and K471H-rev, or K471H-for PB1-rev and (Table S3), with pPolI-PB1-wild-type (46) as the PCR template. The full-length PB1-K471H gene was amplified by PCR using primers PB1-for and PB1-rev. PCR products were digested using *Kpn*I and *Not*I and were cloned into *Kpn*I- and *Not*I-digested pCAGGS-P7 plasmids. The resultant plasmid was designated pCAGGS-PB1-K471H. Likewise, the pCAGGS-PB1-K471 mutant plasmids were constructed using the primers listed in Table S3. The preparation of pCAGGS-PB1-K479H, -K479R, -K480H, -K480R, -K481H, and -K481R has been described previously (12).

### Mini-replicon reporter assay system

293T cells were transfected with expression plasmids encoding PB1 (pCAGGS-PB1-wild-type or PB1-K471 mutants), PB2, PA, and NP, and a plasmid (pHH-vNS-Luc) for expressing the artificial influenza virus genome containing the Firefly luciferase gene in the negative-sense, which was synthesized in cells by the human DNA-dependent RNA polymerase I (PolI) (47). Its negative-sense RNA containing the Firefly luciferase gene was sandwiched by 5′- and 3′-terminal untranslated regions of segment eight genome encoding non-structural protein of the influenza A virus. The mRNA encoding Firefly luciferase was transcribed in an influenza viral RNA polymerase-dependent manner. Luciferase activity was determined using the Dual-Luciferase Reporter Assay System (Promega) according to the manufacturer’s protocol and was normalized to Renilla luciferase activity encoded by the co-transfected pRL-40 vector (Promega).

### Plasmid construction for generating recombinant viruses

To generate plasmids encoding an amino acid point mutation at the Lys471 residue of the PB1, the pPolI-PB1-wild-type plasmid was used as the backbone vector (46). To construct the plasmid from which PolI transcribes the PB1-K471H viral RNA, we amplified two DNA fragments corresponding to the PB1-coding sequence by PCR using primers Pol1-for and K471H-rev, or K471H-for and Pol1-rev (Table S3), with pPolI-PB1-wild-type as the PCR template. The full-length PB1-K471H gene was amplified by PCR using primers Pol1-for and Pol1-rev. The PCR product was digested using *Apa*I and *Xho*I and cloned into the *Apa*I- and *Xho*I-digested pPolI plasmid. The resultant plasmid was designated pPolI-PB1-K471H. Likewise, pPolI-PB1-K471 mutant plasmids were constructed using the primers listed in Table S3. Preparation of pPolI-PB1-K479H, -K479R, -K480H, and -K480R has been described (12).

To construct pPolI-PB1-A349V or pPolI-PB1-A349V/K471H double mutant plasmids, we amplified two DNA fragments corresponding to the PB1-coding sequence by PCR, using primers Pol1-for and A349V-rev or A349V-for and Pol1-rev (Table S3) with pPolI-PB1-wild-type or pPolI-PB1-K471H plasmids as the PCR template. The full-length PB1-A349V or PB1-A349V/K471H gene was amplified by PCR using primers Pol1-for and Pol1-rev. The PCR product was digested using *Apa*I and *Xho*I and cloned into the *Apa*I- and *Xho*I-digested pPolI plasmid. The resultant plasmid was designated pPolI-PB1-A349V or pPolI-PB1-A349V/K471H. Likewise, pPolI-PB1-double mutant plasmids containing A349V and K471 mutations were constructed using each pPolI-PB1-K471 mutant plasmids as the PCR template.

To generate a PR8-based temperature-sensitive strain, designated PR8-FluMist, we constructed pPolI-PB1-K391E/E581G/A661T and pPolI-PB2-N265S plasmids (18, 20, 24). To construct pPolI-PB1-K391E/E581G/A661T plasmids, we amplified two DNA fragments corresponding to PB1-K391E/E581G or PB1-A661T mutant sequences by PCR using primer set PB1-3mut-for-1 and PB1-3mut-rev-1 or PB1-3mut-for-2 and Pol1-rev (Table S3) with synthetic DNA coding PR8-PB1-K391E/E581G or PR8-PB1-A661T fragments (gBlocks Gene Fragments, Integrated DNA Technologies) (Table S4) as the PCR template. Then, the PB1-K391E/E581G/A661T fragment (the 3′-fragment of PB1 sequence, 1,078 to 2,274) was amplified by PCR using primer PB1-3mut-for-1 and Pol1-rev. The 5′-fragment of PB1 sequence was amplified by PCR using primer PolI-for and PB1-3mut-rev-2 with pPolI-PB1-wild-type as the PCR template. The full-length PB1-K391E/E581G/A661T gene was amplified by PCR using primers Pol1-for and Pol1-rev. The PCR product was digested using *Apa*I and *Xho*I and cloned into the *Apa*I- and *Xho*I-digested pPolI plasmid. The resultant plasmid was designated pPolI-PB1-K391E/E581G/A661T. To construct the pPolI-PB2-N265S plasmid, we amplified two DNA fragments corresponding to the PB2-coding sequence by PCR using primers Pol1-BsaI-for and PB2-N256S-rev, or PB2-N256S-for and Pol1-BsaI-rev (Table S3), with pPolI-PB2-wild-type as the PCR template. The full-length PB2-N265S gene was amplified by PCR using primers Pol1-BsaI-for and Pol1-BsaI-rev. The PCR product was digested using *Bsa*I and cloned into the *BsmB*I-digested pPolI plasmid. The resultant plasmid was designated pPolI-PB2-N265S.

### Generation of recombinant influenza viruses

Recombinant influenza viruses were generated using the reverse-genetics system. 293T cells were transfected with eight segmented viral RNAs and viral protein expression plasmids using Trans-IT transfection reagent (Mirus). Twenty-four hours post-transfection, the culture medium was changed to Opti-MEM I (Thermo Fisher Scientific) containing 3.5 µg/mL N-p-tosyl-L-phenylalanine chloromethyl ketone-treated trypsin (Sigma-Aldrich). After incubation for 48 h at 34°C, the cell culture supernatant was collected. The seed virus titer in the supernatant was determined using a plaque assay; then, 200 PFU of virus was inoculated into 10- to 11-day-old embryonated chicken eggs to amplify the recovered viruses.

### Plaque assay

The virus titer was determined using a plaque assay as described previously (48, 49). Briefly, 1 mL aliquots of serial 10-fold dilutions of viruses were inoculated into MDCK cells seeded in 6-well or 12-well plates. After 1 h incubation, each well was overlaid with 3 mL or 1.5 mL of a MEM (Sigma-Aldrich) and 0.8% agarose (Sigma-Aldrich) mixture containing 0.2% bovine albumin (Sigma-Aldrich), 1× vitamin solution (Gibco), 1× MEM amino acid solution (Gibco), 4 µg/mL N-p-tosyl-L-phenylalanine chloromethyl ketone-treated trypsin, and 10 U/mL penicillin-streptomycin (Gibco). The number of plaques was counted following Amido Black 10B (Fujifilm Wako Pure Chemical Corporation) staining or immunostaining method after 3 to 5 days of inoculation. The virus titer was calculated as PFU/mL.

### HA assay and HAI assay

A HA assay and HAI assay were conducted with 0.5% chicken or 1% guinea pig red blood cells (Nippon Bio-Test laboratories) using the standard method (50).

### Immunostaining of virus plaque in 12-well or 24-well plate

MDCK cells in collagen-coated 12-well or 24-well tissue culture plates (AGC TECHNO GLASS) were washed with serum-free DMEM and then infected with 1 to 20 PFUs of the influenza virus. After virus adsorption at 34°C for 1 h, the cells were washed with serum-free DMEM and then overlaid with agarose medium as described above for methods of plaque assay. After 3 days of incubation, the cells were fixed with 4% formaldehyde solution for 1 h at 25°C and then washed with PBS containing 0.1% Triton X-100 (Sigma-Aldrich). The cells were blocked with Blocking One (Nacalai Tesque) with PBS containing 0.1% Triton X-100 for 15 min, and then probed with anti-NP mouse monoclonal antibody (ab128193, Abcam) for 30 min. The cells were washed with PBS containing 0.1% Triton X-100 and incubated with IRDye 800CW Goat anti-Mouse IgG (LI-COR Biosciences) and 0.1 mM CellTag700 (LI-COR Biosciences) for 30 min. After washing with PBS, the virus plaque was detected with the Odyssey CLx Infrared Imaging System (LI-COR Biosciences).

### Western blotting

293T cells were lysed in 20 mM Tris-HCl (pH 7.9), 100 mM NaCl, and 0.1% Triton X-100. After sonication, homogenates were centrifuged 14,000 × *g* at 4°C for 5 min, and the supernatant fractions were separated by sodium dodecyl sulfate-polyacrylamide gel electrophoresis and transferred to polyvinylidene difluoride membranes (pore size 0.45 µm, Merck Millipore). The membranes were blocked with Blocking One (Nacalai Tesque) in Tris-buffered saline containing 0.1% Tween-20 (TBS-T) and probed with anti-PB1 rabbit polyclonal antibody (51), anti-β-actin rabbit polyclonal antibody (PM053, Medical & Biological Laboratories), anti-β-actin mouse monoclonal antibody (M177-3, Medical & Biological Laboratories). The membranes were washed with TBS-T and incubated with IRDye 800CW Goat anti-Rabbit IgG (LI-COR Biosciences) or IRDye 680RD Goat anti-mouse IgG (LI-COR Biosciences). After washing with TBS-T, the proteins were detected with the Odyssey CLx Infrared Imaging System.

### Analyses of nucleic acid substitutions

Viral RNA was purified from the virions using QIAamp Viral RNA Mini Kit (Qiagen), and cDNA derived from PB1, PB2, PA, and NP, which constitute the viral ribonucleoprotein complex, was generated using specific primers and SuperScript III One-Step RT-PCR System with Platinum Taq DNA Polymerase (Thermo Fisher Scientific). After amplicon generation, Sanger sequencing was performed using SeqStudio Genetic Analyzer (Thermo Fisher Scientific).

### Mouse immunization and challenge assay

Six-week-old C57BL/6J female mice were immunized intranasally with 20 µL of PR8-PB1-K471H, PR8-PB1-K471P, or PR8-FluMist viruses (10^6^ PFU/mouse). As a control, mice in the non-immunized group were injected with the same volume of PBS. On day 28, the animals were challenged with 10^7^ PFU of PR8-wild-type virus. Weight loss and mortality were monitored for 14 days after challenge. Mice with body weight loss of more than 25% of their baseline body weight were euthanized. Lungs were collected on days 2, 4, and 6 post-infection and frozen at −80°C in the absence of buffer. Mice were humanely euthanized at the end of the observation period, or at designated time points for tissue collection.

### Virus titration in lung tissues

Lungs were thawed, weighed, and then homogenized in 1 mL of MEM (Sigma-Aldrich) containing 0.2% bovine albumin (Sigma-Aldrich), 1× vitamin solution (Gibco), 1× MEM amino acid solution (Gibco), and 10 U/mL penicillin-streptomycin (Gibco) by using a beads crusher µT-12 (TAITEC) at 2,600 rpm for 1 min. Homogenates were centrifuged (8,000 × g at 4°C for 10 min) to remove debris, and virus titers in cleared homogenate supernatants were determined by plaque assay. Virus titers were normalized to PFU per gram (g) of lung tissue.

### Generation of a mouse-adapted 6:2 reassortant virus

The HA and NA genes of the A/Hong Kong/MA(mouse adapted)/1968/H3N2 virus (GenBank accession number of HA, CY112249.1: GenBank accession number of NA, HM641200.1) were amplified by PCR to construct pPolI-maH3N2-HA and pPolI-maH3N2-NA plasmids, and we subsequently generated a PR8-based 6:2 reassortant virus by using the reverse-genetics system, designated PR8-maH3N2(6:2). To the constructed pPolI-maH3N2-HA plasmid, we amplified full-length maH3N2-HA sequence by PCR using primers Pol1-BsaI-for-2 and Pol1-BsaI-rev (Table S3) with synthetic DNA coding HA 5′-fragment (maH3N2-HA-1, 1 to 856) and HA 3′-fragment (maH3N2-HA-2, 830 to 1,765) (gBlocks Gene Fragments) (Table S4) as the PCR template. The PCR product was digested using *Bsa*I and cloned into the *BsmB*I-digested pPolI plasmid. The resultant plasmid was designated pPolI-maH3N2-HA. To the constructed pPolI-maH3N2-NA plasmid, we amplified full-length maH3N2-NA sequence by PCR using primers Pol1-BsmB-for and Pol1-BsmB-rev (Table S3) with synthetic DNA coding maH3N2-NA (gBlocks Gene Fragments) (Table S4) as the PCR template. The PCR product was digested using *BsmB*I and cloned into the *BsmB*I-digested pPolI plasmid. The resultant plasmid was designated pPolI-maH3N2-NA. To create the 6:2 reassortant virus, eight pPolI plasmids (to express viral RNAs encoding HA and NA of maH3N2 and to express six internal proteins of PR8) were cotransfected with viral protein expression plasmids into 293T cells. The supernatant was collected at 48 h post-transfection and was infected into 10- or 11-day-old embryonated chicken eggs to amplify the recovered viruses. The resultant recombinant strain was designated PR8-maH3N2(6:2) virus.

### ELISpot assay

To detect antigen-specific cytotoxic T lymphocytes, we used the mouse IFN-γ ELISpot Kit (Cellular Technology Limited) to measure the number of IFN-γ-producing spleen cells in response to stimulation with influenza viral peptides. The splenocytes were plated at 1 × 10^5^ cell/well or 2 × 10^5^ cell/well onto IFN-γ antibody-coated ELISpot plates and stimulated with 0.25 µg/mL of NP_366–374_ peptide (MHC Pentamer, Pro5, H-2Db, ASNENMETM, ProImmune) or 0.25 µg/mL of PA_224-233_ peptide (MHC Pentamer, Pro5, H-2Db, SSLENFRAYV, ProImmune) for 24 h at 37°C, respectively. Spots derived from production of IFN-γ were visualized according to the manufacturer’s protocol, and plates were read using ImmunoSpot S6 Universal M2 analyzer (Cellular Technology Limited) and ImmunoCapture 7.0 software (Cellular Technology Limited). SFUs were enumerated using ImmunoSpot 7.0 software (Cellular Technology Limited).

### Statistical analysis

Statistical analyses were performed with Microsoft Excel and KaleidaGraph 5 (HULINKS) software. Quantified luciferase activity was plotted as mean ± SD. Details of statistical tests, sample sizes as well as *P*-values are described for each experiment in respective figure legends. For mice body weight data, both mean and SEM were plotted. Differences in survival curves of infected mice were analyzed using the log-rank test.

## Data Availability

All data supporting the findings in this study are available in this paper.

## SUPPLEMENTAL MATERIAL

Supplemental material jvi.01390-25-s0001.pdf Supplemental text and methods, Figures S1 to S5, and Tables S1 to S4.

ASM does not own the copyrights to Supplemental Material that may be linked to, or accessed through, an article. The authors have granted ASM a non-exclusive, world-wide license to publish the Supplemental Material files. Please contact the corresponding author directly for reuse.
