# Supplementary material for: Live-attenuated influenza virus vaccine strain with an engineered temperature-sensitive and genetically stable viral polymerase variant
Source: J Virol. 2025 Nov 13;99(12):e01390-25. doi: 10.1128/jvi.01390-25 (PMC12724131; doi:10.1128/jvi.01390-25)
Supplement: Supplemental material — Supplemental text and methods, Figures S1 to S5, and Tables S1 to S4. [file jvi.01390-25-s0001.pdf]

Supplementary Materials for

Live-attenuated influenza virus vaccine strain with an engineered temperature-sensitive and genetically stable viral polymerase variant.

Tadasuke Naito, *et al.*,

Address correspondence to Tadasuke Naito, tadanaito@med.kawasaki-m.ac.jp

This PDF file includes:

Text: Supplementary Text, and Supplementary Materials and Methods
Figures: Fig. S1 to Fig. S5
Tables: Table S1 to Table S4
References: Supplementary Reference #1 to #4

Supplementary Text

A revertant virus canceled temperature-sensitive phenotype did not appear from PR8-PB1-K471P strain after serial passages in cell culture at gradually elevated temperatures.

According to previous study, propagation of the virus under controlled laboratory conditions has shown that a FluMist vaccine strain could regain influenza pathogenicity and cause severe disease in mice (1). The result of viral genome sequencing analysis of isolated its revertant virus has identified seven nonsynonymous mutations on PB1, PA, NP, and NS proteins. In this study, to evaluate the genetic stability of PR8-PB1-K471P strain, we performed the serial virus passages in cell culture at gradually elevated temperatures according to the previous investigation. The PR8-PB1-K471P and the PR8-FluMist virus were passaged at gradually elevated temperatures from at 30°C to 39°C for 11 passages in MDCK cells (**Fig. S1A**). Although PR8-wild type virus proliferated in the temperature range from at 31°C to 39°C, we did not observe viral plaque of the parental stock of PR8-PB1-K471P or PR8-FluMist by Amido Black 10B staining in non-permissive temperatures, at 37°C and 39°C (**Fig. S1B**).

According to the four independent experiments of virus serial passages, the PR8-FluMist virus that underwent 11 passages at higher temperatures formed obvious plaques at the restrictive temperature, at 37°C and 39°C (**Fig. S1C**). Then, we confirmed the sequence of the viral genomes derived from the PR8-FluMist-P11 that adapted to the nonpermissive temperatures. To identify an amino acid mutation(s) in viral protein which compose the vRNP complex derived from PR8-FluMist-P11, we analyzed three polymerase subunits and NP using Sanger sequencing. The amino acid changes detected among the PR8-FluMist-P11 viruses that adapted to the nonpermissive temperatures are summarized in **Table S1**. Four substitutions, E391K and G581E, or R70K and D701N, were detected in PB1 or PB2, respectively. Three substitutions, D3N, E377K, and Q556R, were identified in PA, while there was no substitution in NP. On the other hand, adaptation to nonpermissive temperature of the PR8-PB1-K471P virus was not observed after 11 passages: infectious virus could not be detected from the supernatant collected as PR8-PB1-K471P-P11 in plaque assays performed at either permissive or nonpermissive temperatures (data not shown). These results suggested that the PR8-PB1-K471P strain which resulted in the genetically stable phenotype prevented reversal of the temperature-sensitive phenotype.

Viral growth kinetics of PR8-wild type, PR8-FluMist, and PR8-PB1-K471P viruses at various temperatures.

To investigate the replication kinetics of vaccine strains in cultured cells, we infected MDCK cells with PR8-wild type, PR8-FluMist, or PR8-PB1-K471P, and the amount of progeny virus was measured by plaque assays (**Fig. S2**). PR8-wild type, PR8-FluMist, and PR8-PB1-K471P had similar virus titers in the supernatant at 31°C culture for 2 days post infection (dpi), namely, 5.0×10^7 PFU/ml, 5.3×10^7 PFU/ml, and 2.9×10^7 PFU/ml, respectively (**Fig. S2, left panel**). In contrast, the viral titer of PR8-FluMist was lower than that of PR8-wild type at 34°C and 37°C. (*e.g.*, from 2 dpi, the virus titers of PR8-wild type at 34°C or 37°C were 9.7×10^7 PFU/ml or 4.0×10^7 PFU/ml, while those of PR8-FluMist were 1.1×10^6 PFU/ml or 1.6×10^5 PFU/ml, respectively) (**Fig. S2, middle and right panels**). The viral titer level of PR8-PB1-K471P was 88% lower than that of PR8-wild type after 2 dpi at 34°C, and then maximum titers from PR8-PB1-K471P was 1.9×10^6 PFU/ml at 3 dpi (*e.g.*, from 2 dpi, the virus titers of PR8-PB1-K471P at 34°C was 1.1×10^6 PFU/ml). In addition, although the progeny virus production did not detect from PR8-PB1-K471P at 37°C, the virus titers from PR8-FluMist gradually increased after at 3 dpi. These results indicated that PR8-PB1-K471P strain exhibited the temperature-sensitive replication kinetics, that although the virus titers reached to 10^7 PFU/ml at 31°C, the progeny virus could not appear from infected MDCK cells at 37°C. Furthermore, PR8-FluMist is highly likely to have generated revertant mutant viruses that lose their temperature-sensitive phenotype under viral replication conditions at 37°C. This result was consistent with the trends observed in the virus passage results shown in **Fig. S1**.

The small viral plaque was detected in PR8-FluMist infected cells at 37°C using by immunostaining.

To evaluate the temperature sensitivity of PR8-FluMist strain, we performed plaque assay using immunostaining at different temperatures. As shown in **Fig. S3**, although the viral plaque formation caused by expression of NP could not detect in PR8-PB1-K471P-infected cells at 37°C, small viral plaques were observed at 37°C compared to 31°C or 34°C due to expression of NP in PR8-FluMist-infected cells. PR8-wild type could form clear plaques at 31°C to 39°C, but we did not observe viral plaque in PR8-FluMist-infected cells at 39°C.

These results indicated that the temperature-sensitive phenotype of PR8-PB1-K471P variant is pronounced than that of PR8-FluMist.

The production of recombinant 6:2 or 7:1 vaccine virus based on PR8-PB1-K471P as the parent strain that encoding genes for HA and/or NA derived from seasonal influenza viruses was possible.

To investigate the suitability of PR8-PB1-K471P as a live-attenuated vaccine strain, we were create PR8-PB1-K471P-based 6:2 or 7:1 vaccine virus encoding the HA and/or the NA of seasonal influenza viruses. As shown in **Fig. S5** and **Table S2**, propagation of each master virus in chicken eggs was examined via HA test. The PR8-PB1-K471P-based 6:1 or 7:1 vaccine virus encoding the HA and/or the NA of A/Victoria/1/2020/H1H1pdm strain were designated as PR8-PB1-K471P-6:2(H1N1) and PR8-PB1-K471P-7:1(H1N1), respectively. HA titers of primary isolates of the PR8-PB1-K471P-6:2(H1N1) and the PR8-PB1-K471P-7:1(H1N1) were 64 and 256 HA units, respectively. Likewise, we attempted to generate the PR8-PB1-K471P-6:2(H3N2) and the PR8-PB1-K471P-7:1(H3N2) encoding the HA and/or the NA of a seasonal influenza virus, A/Tasmania/503/2020/H3N2 strain. As a result, although the PR8-PB1-K471P-7:1(H3N2) could create as PR8-PB1-K471P-based vaccine virus (32 HA unit), the PR8-PB1-K471P-6:2(H3N2) virus replicated very poorly in eggs, with titers of lower than 2 HA units (data not shown). In order to improve the HA yield in eggs of the vaccine candidate virus is required to undergo egg adaptation via serial passages in eggs (2-4). We test whether serial passages in eggs would improve the proliferation of the PR8-PB1-K471P-6:2(H3N2) virus, and intend to report in future study. To compare the vaccine effect of the PR8-PB1-K471P-based vaccines, the PR8-FluMist-6:2(H1N1), the PR8-FluMist-7:1(H1N1), or the PR8-FluMist-7:1(H3N2) virus also was collected as E1 stock virus: HA titers of each PR8-FluMist-based vaccine virus were 64, 256, and 128 HA units, respectively (**Table S2**).

Supplementary Materials and Methods

Viral serial passages in MDCK cells at gradually elevated temperature.

Confluent MDCK cells were infected with PR8-FluMist or PR8-PB1-K471P viruses at the MOI of 0.1 at 30°C, and incubated for 3 to 4 days containing 1 ml of the virus growth medium in 12-well culture plate. When a cytopathic effect was observed, supernatant was collected and named P1 (passage 1). Ten μl of the P1 virus stock was transferred to a fresh MDCK cells in 12-well culture plate; 3 to 4 days later, the supernatant (P2) was collected. The P2 virus stock was further passaged and repeated 11 times in MDCK cells at gradually elevated temperature, as illustrated in **Fig. S1A** by reference to a previous article for investigation of reversion mutation of cold-adapted live attenuated influenza vaccine (1).

Viral replication kinetics in MDCK cells.

Confluent MDCK cells in 12-well tissue culture plates were infected with PR8-wild type, PR8-FluMist or PR8-PB1-K471P viruses at the MOI of 0.01 at 31°C, 34°C and 37°C. After two hours post inoculation, the cells were washed twice and cell culture wells were refilled with 3 ml of a virus growth medium: MEM containing 0.2% bovine albumin, 1 × vitamin solution, 1 × MEM amino acid solution, 1 μ/ml N-p-tosyl-L-phenylalanine chloromethyl ketone-treated trypsin and 10 U/ml penicillin-streptomycin. Supernatants were collected at from 1 day to 6 days post infection. All virus titers were determined by plaque assay using MDCK cells.

Plasmid construction for generating recombinant A/Victoria/1/2020/H1N1pdm and A/Tasmania/503/2020/H3N2 viruses.

To generate a recombinant A/Victoria/1/2020/H1N1pdm (GDAID isolate ID, EPI_ISL_594220) strain, we constructed pPolI-Victoria/H1N1-PB1, pPolI-Victoria/H1N1-PB2, pPolI-Victoria/H1N1-PA, pPolI-Victoria/H1N1-HA, pPolI-Victoria/H1N1-NP, pPolI-Victoria/H1N1-NA, pPolI-Victoria/H1N1-M, and pPolI-Victoria/H1N1-NS plasmids. To construct pPolI-Victoria/H1N1-PB1 plasmid, we amplified three DNA fragments corresponding to $PB1_{(1\text{-}716\ nt)}$, $PB1_{(692\text{-}1,431\ nt)}$, and $PB1_{(1,412\text{-}2,341\ nt)}$ sequences by PCR using primer set Pol1-BsmB-for-2 and Vic/H1N1-PB1-750-rev, Vic/H1N1-PB1-750-for and Vic/H1N1-PB1-1468-rev, or Vic/H1N1-PB1-1468-for and Pol1-BsmB-rev (**Table S3**), with synthetic DNA coding Victoria/H1N1-PB1-5’, Victoria/H1N1-PB1-middle, or

Victoria/H1N1-PB1-3' fragments (gBlocks Gene Fragments, Integrated DNA Technologies) (**Table S4**) as the PCR templates. Then, the Victoria/H1N1-PB1-5'-middle fragment (1-1,431 nt) was amplified by PCR using primer Pol1-BsmB-for-2 and Vic/H1N1-PB1-1468-rev with PB1$_{(1\text{-}716\ nt)}$ and PB1$_{(692\text{-}1,431\ nt)}$ DNA products as the PCR template. The full-length Victoria/H1N1-PB1 gene was amplified by PCR using primers Pol1-BsmB-for-2 and Pol1-BsmB-rev with PB1$_{(1\text{-}1,431\ nt)}$ and PB1$_{(1,412\text{-}2,341\ nt)}$ DNA products as the PCR template. PCR product was digested with *Bsm*BI and cloned into the *Bsm*BI-digested pPolI plasmid. The resultant plasmid was designated pPolI-Victoria/H1N1-PB1. To construct pPolI-Victoria/H1N1-PB2 plasmid, we amplified three DNA fragments corresponding to PB2$_{(1\text{-}716\ nt)}$, PB2$_{(692\text{-}1,431\ nt)}$, and PB2$_{(1,412\text{-}2,341\ nt)}$ sequences by PCR using primer set Pol1-BsaI-for and Vic/H1N1-PB2-732-rev, Vic/H1N1-PB2-732-for and Vic/H1N1-PB2-1447-rev, or Vic/H1N1-PB2-1447-for and Pol1-BsaI-rev (**Table S3**), with synthetic DNA coding Victoria/H1N1-PB2-5', Victoria/H1N1-PB2-middle, or Victoria/H1N1-PB2-3' fragments (gBlocks Gene Fragments, Integrated DNA Technologies) (**Table S4**) as the PCR templates. Then, the Victoria/H1N1-PB2-5'-middle fragment (1-1,447 nt) was amplified by PCR using primer Pol1-BsaI-for and Vic/H1N1-PB2-1447-rev with PB2$_{(1\text{-}716\ nt)}$ and PB2$_{(692\text{-}1,431\ nt)}$ DNA products as the PCR template. The full-length Victoria/H1N1-PB2 gene was amplified by PCR using primers Pol1-BsaI-for and Pol1-BsaI-rev with PB2$_{(1\text{-}1,447\ nt)}$ and PB2$_{(1,412\text{-}2,341\ nt)}$ DNA products as the PCR template. PCR product was digested with *Bsa*I and cloned into the *Bsm*BI-digested pPolI plasmid. The resultant plasmid was designated pPolI-Victoria/H1N1-PB2. To construct pPolI-Victoria/H1N1-PA plasmid, we amplified three DNA fragments corresponding to PA$_{(1\text{-}708\ nt)}$, PA$_{(684\text{-}1,417\ nt)}$, and PA$_{(1,391\text{-}2,233\ nt)}$ sequences by PCR using primer set Pol1-BsmB-for-2 and Vic/H1N1-PA-724-rev, Vic/H1N1-PA-724-for and Vic/H1N1-PA-1433-rev, or Vic/H1N1-PA-1433-for and Pol1-BsmB-rev (**Table S3**), with synthetic DNA coding Victoria/H1N1-PA-5', Victoria/H1N1-PA-middle, or Victoria/H1N1-PA-3' fragments (gBlocks Gene Fragments, Integrated DNA Technologies) (**Table S4**) as the PCR templates. Then, the Victoria/H1N1-PA-5'-middle fragment (1-1,417 nt) was amplified by PCR using primer Pol1-BsmB-for-2 and Vic/H1N1-PA-1433-rev with PA$_{(1\text{-}708\ nt)}$ and PA$_{(684\text{-}1,417\ nt)}$ DNA products as the PCR template. The full-length Victoria/H1N1-PA gene was amplified by PCR using primers Pol1-BsmB-for-2 and Pol1-BsmB-rev with PA$_{(1\text{-}1,417\ nt)}$ and PA$_{(1,391\text{-}2,233\ nt)}$ DNA products as the PCR template. PCR product was digested with *Bsm*BI and cloned into the *Bsm*BI-digested pPolI plasmid. The resultant plasmid was designated pPolI-Victoria/H1N1-PA. To construct pPolI-Victoria/H1N1-HA plasmid, we amplified two

DNA fragments corresponding to $HA_{(1\text{-}873\ nt)}$ and $HA_{(850\text{-}1,778\ nt)}$ sequences by PCR using primer set Pol1-BsmB-for-2 and Vic/H1N1-HA-889-rev, or Vic/H1N1-HA-889-for and Pol1-BsmB-rev (**Table S3**), with synthetic DNA coding Victoria/H1N1-HA-5’ or Victoria/H1N1-HA-3’ fragments (gBlocks Gene Fragments, Integrated DNA Technologies) (**Table S4**) as the PCR templates. Then, the full-length Victoria/H1N1-HA gene was amplified by PCR using primers Pol1-BsmB-for-2 and Pol1-BsmB-rev with $HA_{(1\text{-}873\ nt)}$ and $HA_{(850\text{-}1,778\ nt)}$ DNA products as the PCR template. PCR product was digested with *Bsm*BI and cloned into the *Bsm*BI-digested pPolI plasmid. The resultant plasmid was designated pPolI-Victoria/H1N1-HA. To construct pPolI-Victoria/H1N1-NP plasmid, we amplified two DNA fragments corresponding to $NP_{(1\text{-}696\ nt)}$ and $NP_{(673\text{-}1,565\ nt)}$ sequences by PCR using primer set Pol1-BsmB-for-2 and Vic/H1N1-NP-712-rev, or Vic/H1N1-NP-712-for and Pol1-BsmB-rev (**Table S3**), with synthetic DNA coding Victoria/H1N1-NP-5’ or Victoria/H1N1-NP-3’ fragments (gBlocks Gene Fragments, Integrated DNA Technologies) (**Table S4**) as the PCR templates. Then, the full-length Victoria/H1N1-NP gene was amplified by PCR using primers Pol1-BsmB-for-2 and Pol1-BsmB-rev with $NP_{(1\text{-}696\ nt)}$ and $NP_{(673\text{-}1,565\ nt)}$ DNA products as the PCR template. PCR product was digested with *Bsm*BI and cloned into the *Bsm*BI-digested pPolI plasmid. The resultant plasmid was designated pPolI-Victoria/H1N1-NP. To construct pPolI-Victoria/H1N1-NA plasmid, we amplified two DNA fragments corresponding to $NA_{(1\text{-}620\ nt)}$ and $NA_{(596\text{-}1,458\ nt)}$ sequences by PCR using primer set Pol1-BsmB-for-2 and Vic/H1N1-NA-636-rev, or Vic/H1N1-NA-636-for and Pol1-BsmB-rev (**Table S3**), with synthetic DNA coding Victoria/H1N1-NA-5’ or Victoria/H1N1-NA-3’ fragments (gBlocks Gene Fragments, Integrated DNA Technologies) (**Table S4**) as the PCR templates. Then, the full-length Victoria/H1N1-NA gene was amplified by PCR using primers Pol1-BsmB-for-2 and Pol1-BsmB-rev with $NA_{(1\text{-}620\ nt)}$ and $NA_{(596\text{-}1,458\ nt)}$ DNA products as the PCR template. PCR product was digested with *Bsm*BI and cloned into the *Bsm*BI-digested pPolI plasmid. The resultant plasmid was designated pPolI-Victoria/H1N1-NA. To construct pPolI-Victoria/H1N1-M plasmid, we amplified two DNA fragments corresponding to $M_{(1\text{-}430\ nt)}$ and $M_{(407\text{-}1,027\ nt)}$ sequences by PCR using primer set Pol1-BsmB-for-2 and Vic/H1N1-MP-446-rev, or Vic/H1N1-MP-446-for and Pol1-BsmB-rev (**Table S3**), with synthetic DNA coding Victoria/H1N1-M-5’ or Victoria/H1N1-M-3’ fragments (gBlocks Gene Fragments, Integrated DNA Technologies) (**Table S4**) as the PCR templates. Then, the full-length Victoria/H1N1-M gene was amplified by PCR using primers Pol1-BsmB-for-2 and Pol1-BsmB-rev with $M_{(1\text{-}430\ nt)}$ and $M_{(407\text{-}1,027\ nt)}$ DNA products as the PCR

template. PCR product was digested with *Bsm*BI and cloned into the *Bsm*BI-digested pPolI plasmid. The resultant plasmid was designated pPolI-Victoria/H1N1-M. To construct pPolI-Victoria/H1N1-NS plasmid, we amplified two DNA fragments corresponding to $NS_{(1\text{-}439\ nt)}$ and $NS_{(412\text{-}890\ nt)}$ sequences by PCR using primer set Pol1-BsmB-for-2 and Vic/H1N1-NS-455-rev, or Vic/H1N1-NS-455-for and Pol1-BsmB-rev (**Table S3**), with synthetic DNA coding Victoria/H1N1-NS-5' or Victoria/H1N1-NS-3' fragments (gBlocks Gene Fragments, Integrated DNA Technologies) (**Table S4**) as the PCR templates. Then, the full-length Victoria/H1N1-NS gene was amplified by PCR using primers Pol1-BsmB-for-2 and Pol1-BsmB-rev with $NS_{(1\text{-}439\ nt)}$ and $NS_{(412\text{-}890\ nt)}$ DNA products as the PCR template. PCR product was digested with *Bsm*BI and cloned into the *Bsm*BI-digested pPolI plasmid. The resultant plasmid was designated pPolI-Victoria/H1N1-NS.

Likewise, to generate a recombinant A/Tasmania/503/2020/H3N2 (GDAID isolate ID, EPI_ISL_584118) strain, we constructed pPolI-Tasmania/H1N1-PB1, pPolI-Tasmania/H1N1-PB2, pPolI-Tasmania/H1N1-PA, pPolI-Tasmania/H1N1-HA, pPolI-Tasmania/H1N1-NP, pPolI-Tasmania/H1N1-NA, pPolI-Tasmania/H1N1-M, and pPolI-Tasmania/H1N1-NS plasmids. To construct pPolI-Tasmania/H1N1-PB1 plasmid, we amplified three DNA fragments corresponding to $PB1_{(1\text{-}686\ nt)}$, $PB1_{(661\text{-}1,389\ nt)}$, and $PB1_{(1,365\text{-}2,341\ nt)}$ sequences by PCR using primer set Pol1-BsmB-for-2 and Tasm/H3N2-PB1-702-rev, Tasm/H3N2-PB1-702-for and Tasm/H3N2-PB1-1405-rev, or Tasm/H3N2-PB1-1405-for and Pol1-BsmB-rev (**Table S3**), with synthetic DNA coding Tasmania/H1N1-PB1-5', Tasmania/H1N1-PB1-middle, or Tasmania/H1N1-PB1-3' fragments (gBlocks Gene Fragments, Integrated DNA Technologies) (**Table S4**) as the PCR templates. Then, the Tasmania/H1N1-PB1-5'-middle fragment (1-1,389 nt) was amplified by PCR using primer Pol1-BsmB-for-2 and Tasm/H3N2-PB1-1405-rev with $PB1_{(1\text{-}686\ nt)}$ and $PB1_{(661\text{-}1,389\ nt)}$ DNA products as the PCR template. The full-length Tasmania/H1N1-PB1 gene was amplified by PCR using primers Pol1-BsmB-for-2 and Pol1-BsmB-rev with $PB1_{(1\text{-}1,389\ nt)}$ and $PB1_{(1,365\text{-}2,341\ nt)}$ DNA products as the PCR template. PCR product was digested with *Bsm*BI and cloned into the *Bsm*BI-digested pPolI plasmid. The resultant plasmid was designated pPolI-Tasmania/H1N1-PB1. To construct pPolI-Tasmania/H1N1-PB2 plasmid, we amplified three DNA fragments corresponding to $PB2_{(1\text{-}697\ nt)}$, $PB2_{(676\text{-}1,404\ nt)}$, and $PB2_{(1,382\text{-}2,341\ nt)}$ sequences by PCR using primer set Pol1-BsaI-for and Tasm/H3N2-PB2-713-rev, Tasm/H3N2-PB2-713-for and Tasm/H3N2-PB2-1420-rev, or Tasm/H3N2-PB2-1420-for and Pol1-BsaI-rev (**Table S3**), with synthetic DNA coding Tasmania/H1N1-PB2-5', Tasmania/H1N1-PB2-

middle, or Tasmania/H1N1-PB2-3’ fragments (gBlocks Gene Fragments, Integrated DNA Technologies) (**Table S4**) as the PCR templates. Then, the Tasmania/H1N1-PB2-5’-middle fragment (1-1,404 nt) was amplified by PCR using primer Pol1-BsaI-for and Tasm/H3N2-PB2-1420-rev with PB2$_{\text{(1-697 nt)}}$ and PB2$_{\text{(676-1,404 nt)}}$ DNA products as the PCR template. The full-length Tasmania/H1N1-PB2 gene was amplified by PCR using primers Pol1-BsaI-for and Pol1-BsaI-rev with PB2$_{\text{(1-1,404 nt)}}$ and PB2$_{\text{(1,382-2,341 nt)}}$ DNA products as the PCR template. PCR product was digested with *Bsa*I and cloned into the *Bsm*BI-digested pPolI plasmid. The resultant plasmid was designated pPolI-Tasmania/H1N1-PB2. To construct pPolI-Tasmania/H1N1-PA plasmid, we amplified three DNA fragments corresponding to PA$_{\text{(1-694 nt)}}$, PA$_{\text{(673-1,408 nt)}}$, and PA$_{\text{(1,379-2,233 nt)}}$ sequences by PCR using primer set Pol1-BsaI-for and Tasm/H3N2-PA-710-rev, Tasm/H3N2-PA-710-for and Tasm/H3N2-PA-1424-rev, or Tasm/H3N2-PA-1424-for and Pol1-BsaI-rev (**Table S3**), with synthetic DNA coding Tasmania/H1N1-PA-5’, Tasmania/H1N1-PA-middle, or Tasmania/H1N1-PA-3’ fragments (gBlocks Gene Fragments, Integrated DNA Technologies) (**Table S4**) as the PCR templates. Then, the Tasmania/H1N1-PA-5’-middle fragment (1-1,408 nt) was amplified by PCR using primer Pol1-BsaI-for and Tasm/H3N2-PA-1424-rev with PA$_{\text{(1-694 nt)}}$ and PA$_{\text{(673-1,408 nt)}}$ DNA products as the PCR template. The full-length Tasmania/H1N1-PA gene was amplified by PCR using primers Pol1-BsaI-for and Pol1-BsaI-rev with PA$_{\text{(1-1,408 nt)}}$ and PA$_{\text{(1,379-2,233 nt)}}$ DNA products as the PCR template. PCR product was digested with *Bsa*I and cloned into the *Bsm*BI-digested pPolI plasmid. The resultant plasmid was designated pPolI-Tasmania/H1N1-PA. To construct pPolI-Tasmania/H1N1-HA plasmid, we amplified two DNA fragments corresponding to HA$_{\text{(1-941 nt)}}$ and HA$_{\text{(916-1,765 nt)}}$ sequences by PCR using primer set Pol1-BsmB-for and Tasm/H3N2-HA-957-rev, or Tasm/H3N2-HA-957-for and Pol1-BsmB-rev (**Table S3**), with synthetic DNA coding Tasmania/H1N1-HA-5’ or Tasmania/H1N1-HA-3’ fragments (gBlocks Gene Fragments, Integrated DNA Technologies) (**Table S4**) as the PCR templates. Then, the full-length Tasmania/H1N1-HA gene was amplified by PCR using primers Pol1-BsmB-for and Pol1-BsmB-rev with HA$_{\text{(1-941 nt)}}$ and HA$_{\text{(916-1,765 nt)}}$ DNA products as the PCR template. PCR product was digested with *Bsm*BI and cloned into the *Bsm*BI-digested pPolI plasmid. The resultant plasmid was designated pPolI-Tasmania/H1N1-HA. To construct pPolI-Tasmania/H1N1-NP plasmid, we amplified two DNA fragments corresponding to NP$_{\text{(1-697 nt)}}$ and NP$_{\text{(674-1,565 nt)}}$ sequences by PCR using primer set Pol1-BsmB-for and Tasm/H3N2-NP-713-rev, or Tasm/H3N2-NP-713-for and Pol1-BsmB-rev (**Table S3**), with synthetic DNA coding Tasmania/H1N1-NP-5’ or

Tasmania/H1N1-NP-3' fragments (gBlocks Gene Fragments, Integrated DNA Technologies) (**Table S4**) as the PCR templates. Then, the full-length Tasmania/H1N1-NP gene was amplified by PCR using primers Pol1-BsmB-for and Pol1-BsmB-rev with $NP_{(1\text{-}697\ nt)}$ and $NP_{(674\text{-}1,565\ nt)}$ DNA products as the PCR template. PCR product was digested with *Bsm*BI and cloned into the *Bsm*BI-digested pPolI plasmid. The resultant plasmid was designated pPolI-Tasmania/H1N1-NP. To construct pPolI-Tasmania/H1N1-NA plasmid, we amplified two DNA fragments corresponding to $NA_{(1\text{-}625\ nt)}$ and $NA_{(601\text{-}1,466\ nt)}$ sequences by PCR using primer set Pol1-BsaI-for-2 and Vic/H1N1-NA-636-rev, or Vic/H1N1-NA-636-for and Pol1-BsaI-rev (**Table S3**), with synthetic DNA coding Tasmania/H1N1-NA-5' or Tasmania/H1N1-NA-3' fragments (gBlocks Gene Fragments, Integrated DNA Technologies) (**Table S4**) as the PCR templates. Then, the full-length Tasmania/H1N1-NA gene was amplified by PCR using primers Pol1-BsaI-for-2 and Pol1-BsaI-rev with $NA_{(1\text{-}625\ nt)}$ and $NA_{(601\text{-}1,466\ nt)}$ DNA products as the PCR template. PCR product was digested with *Bsa*I and cloned into the *Bsm*BI-digested pPolI plasmid. The resultant plasmid was designated pPolI-Tasmania/H1N1-NA. To construct pPolI-Tasmania/H1N1-M plasmid, we amplified two DNA fragments corresponding to $M_{(1\text{-}460\ nt)}$ and $M_{(439\text{-}1,027\ nt)}$ sequences by PCR using primer set Pol1-BsmB-for-2 and Tasm/H3N2-MP-476-rev, or Tasm/H3N2-MP-476-for and Pol1-BsmB-rev (**Table S3**), with synthetic DNA coding Tasmania/H1N1-M-5' or Tasmania/H1N1-M-3' fragments (gBlocks Gene Fragments, Integrated DNA Technologies) (**Table S4**) as the PCR templates.

Then, the full-length Tasmania/H1N1-M gene was amplified by PCR using primers Pol1-BsmB-for-2 and Pol1-BsmB-rev with $M_{(1\text{-}460\ nt)}$ and $M_{(439\text{-}1,027\ nt)}$ DNA products as the PCR template. PCR product was digested with *Bsm*BI and cloned into the *Bsm*BI-digested pPolI plasmid. The resultant plasmid was designated pPolI-Tasmania/H1N1-M. To construct pPolI-Tasmania/H1N1-NS plasmid, we amplified two DNA fragments corresponding to $NS_{(1\text{-}458\ nt)}$ and $NS_{(438\text{-}890\ nt)}$ sequences by PCR using primer set Pol1-BsmB-for-2 and Tasm/H3N2-NS-474-rev, or Tasm/H3N2-NS-474-for and Pol1-BsmB-rev (**Table S3**), with synthetic DNA coding Tasmania/H1N1-NS-5' or Tasmania/H1N1-NS-3' fragments (gBlocks Gene Fragments, Integrated DNA Technologies) (**Table S4**) as the PCR templates. Then, the full-length Tasmania/H1N1-NS gene was amplified by PCR using primers Pol1-BsmB-for-2 and Pol1-BsmB-rev with $NS_{(1\text{-}458\ nt)}$ and $NS_{(438\text{-}890\ nt)}$ DNA products as the PCR template. PCR product was digested with *Bsm*BI and cloned into the *Bsm*BI-digested pPolI plasmid. The resultant plasmid was designated pPolI-Tasmania/H1N1-NS.

Generation of 6:2 or 7:1 reassortant vaccine viruses.

By substituting the untranslated region (UTR) of the original Victoria/H1N1-HA and NA genes, or the original Tasmania/H3N2-HA and NA genes coding to pPolI plasmid with that of PR8, the PR8-based 6:2 or 7:1 reassortant viruses were generated by RG system using by these plasmids. To replace with PR8-UTR sequences from original A/Victoria/1/2020/H1N1pdm or A/Tasmania/503/2020/H3N2 UTRs, we amplified Victoria/H1N1-HA(PR8-UTR), Victoria/H1N1-NA(PR8-UTR), Tasmania/H3N2-HA(PR8-UTR), and Tasmania/H3N2-NA(PR8-UTR) DNA fragments by PCR using primer set Vic/H1N1-HA-PR8utr-for and Vic/H1N1-HA-PR8utr-rev, Vic/H1N1-NA-PR8utr-for and Vic/H1N1-NA-PR8utr-rev, Tasm/H3N2-HA-PR8utr-for and Tasm/H3N2-HA-PR8utr-rev, or Tasm/H3N2-NA-PR8utr-for and Tasm/H3N2-NA-PR8utr-rev (**Table S3**) with pPolI-Victoria/H1N1-HA, pPolI-Victoria/H1N1-NA, pPolI-Tasmania/H3N2-HA, or pPolI-Tasmania/H3N2-NA plasmids as the PCR template. Subsequently, in order to add the restriction enzyme digestion site of *Bsm*BI or *Bsa*I with terminal region of these PCR products, we amplified Victoria/H1N1-HA(PR8-UTR)-BsmBI and Tasmania/H3N2-HA(PR8-UTR)-BsmBI DNA fragments by PCR using primers Pol1-BsmB-for and Pol1-BsmB-rev (**Table S3**) with Victoria/H1N1-HA(PR8-UTR) or Tasmania/H3N2-HA(PR8-UTR) DNA fragments as the PCR template. Likewise, we amplified Victoria/H1N1-NA(PR8-UTR)-BsmBI and Tasmania/H3N2-NA(PR8-UTR)-BsaI DNA fragments by PCR using primer set Pol1-BsmB-for-2 and Pol1-BsmB-rev, or Pol1-BsaI-for and Pol1-BsaI-rev (**Table S3**) with Victoria/H1N1-NA(PR8-UTR) or Tasmania/H3N2-NA(PR8-UTR) DNA fragments as the PCR template. The PCR products were digested using *Bsm*BI or *Bsa*I and cloned into the *BsmB*I-digested pPolI plasmid.

To create the 6:2 reassortant viruses, eight pPolI plasmids [to express viral RNAs encoding HA and NA of A/Victoria/1/2020/H1N1pdm or A/Tasmania/503/2020/H3N2, and to express PB1-wild type or PB1-K471P, and five internal proteins of PR8] were cotransfected with viral protein expression plasmids into 293T cells. Likewise, to create the 7:1 reassortant viruses, eight pPolI plasmids [to express viral RNAs encoding HA of A/Victoria/1/2020/H1N1pdm or A/Tasmania/503/2020/H3N2, and to express PB1-wild type or PB1-K471P, and six internal proteins including NA of PR8] were cotransfected with viral protein expression plasmids into 293T cells. The supernatant was collected at 48 h

posttransfection and was injected into 10- to 11-day old embryonated chicken eggs to amplify the recovered viruses.

Supplementary Figures

Supplementary Figure 1

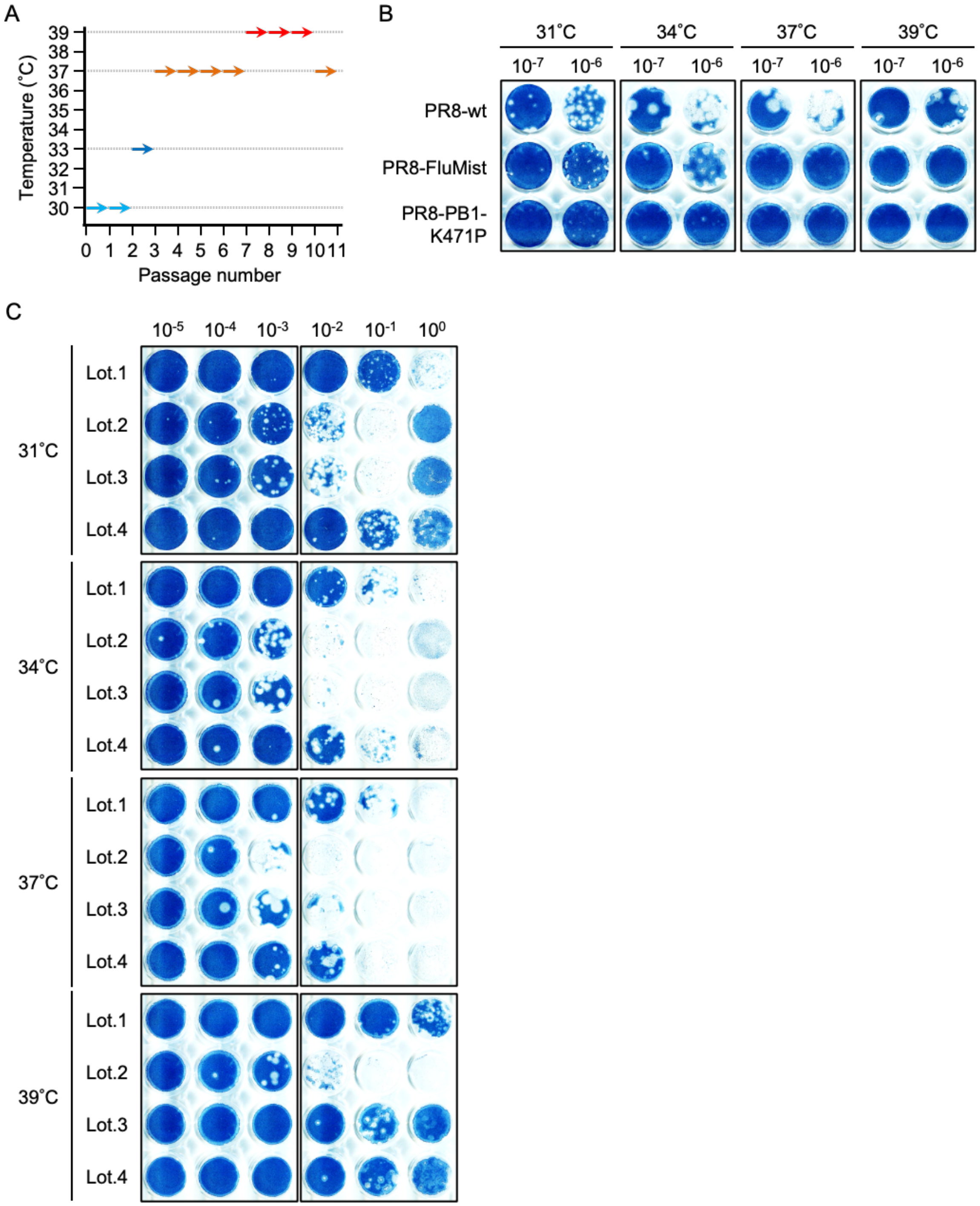

Supplementary Figure 1. Although PR8-PB1-K471P variant maintained the temperature-sensitive phenotype during serial virus passage, the non-temperature-sensitive revertant viruses were generated from PR8-FluMist.

(A) PR8-PB1-K471P and PR8-FluMist viruses were passaged eleven times using by MDCK cells at gradually elevated temperatures.

(B, C) Plaque formation of PR8-wt-P0, PR8-FluMist-P0 and PR8-PB1-K471P-P0 (B), or PR8-FluMist-P11 (C) at 31°C, 34°C, 37°C, or 39°C. Plaque assay was performed by using PB1-wt-P0, PR8-FluMist-P0 or PR8-PB1-K471P-P0 viruses amplified in chicken eggs (B). The serial virus passage experiment was repeated four times independently, and the results of PR8-FluMist-P11 are shown in Panel C as Lot #1 to #4. Serial dilutions of viruses were used to infect MDCK cells at the indicated temperatures. MDCK cells were infected with the viruses indicated at the left of the figure and incubated at 31°C, 34°C, 37°C, or 39°C for 4 days. Virus plaques were visualized by Amido Black 10B staining and photographed.

Supplementary Figure 2

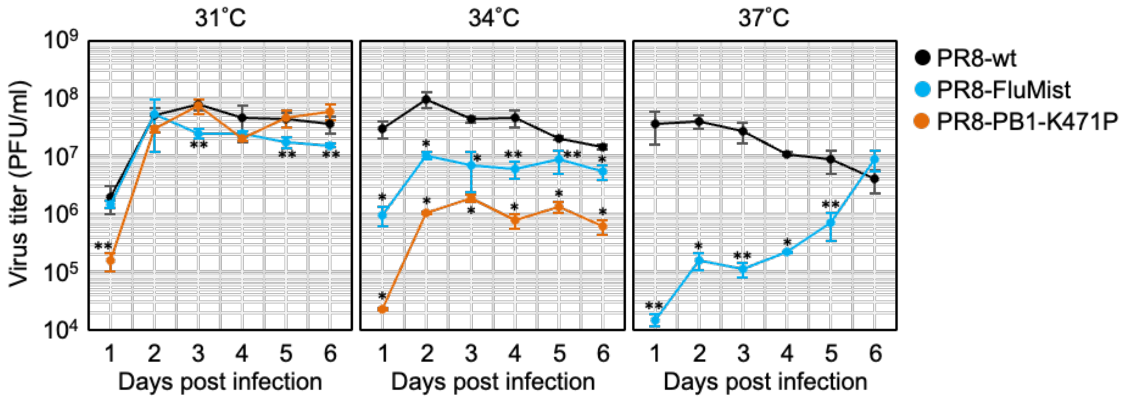

Supplementary Figure 2. Replication of PR8-wt, PR8-FluMist, and PR8-PB1-K471P viruses at various temperatures.

MDCK cells were infected with MOI of 0.01 and incubated with 31°C, 34°C, or 37°C, and culture supernatants were collected at 1, 2, 3, 4, 5, and 6 dpi. Viral titers were determined by plaque assay using MDCK cells. Quantitative results are presented as the average with the standard deviation from at least three independent experiments. Significance was determined using Student's t test. Asterisk indicates viral titer of PR8-FluMist or PR8-PB1-K471P were lower than that of PR8-wt (*, $p < 0.005$; **, $p < 0.05$). Progeny viruses derived PR8-PB1-K471P did not detected from infected cells cultured at 37°C (Right panel).

Supplementary Figure 3

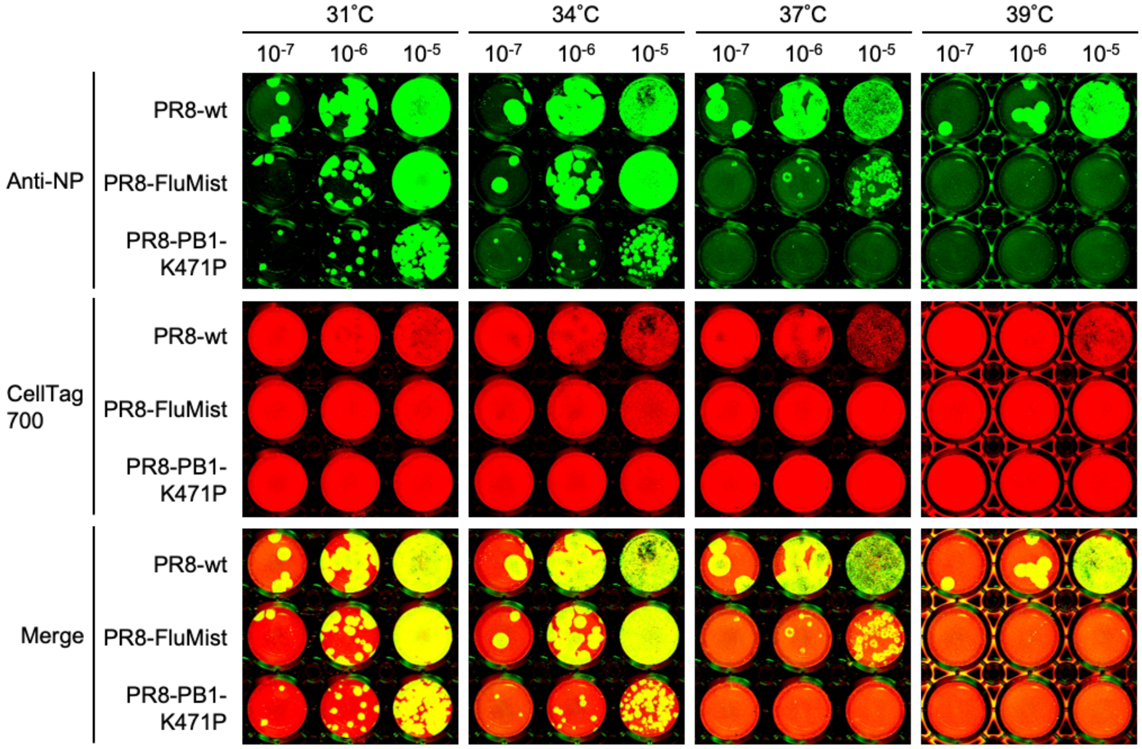

Supplementary Figure 3. The PR8-FluMist virus could synthesize NP protein in infected cells at 37°C but not at 39°C.

Plaque assay was performed by using PR8-PB1-wild type (PR8-wt), PR8-FluMist or PR8-PB1-K471P viruses amplified in chicken eggs. Serial dilutions (10^{-7} to 10^{-5}) of viruses were used to infect MDCK cells at the indicated temperatures. MDCK cells were infected with the viruses indicated at the left of the figure and incubated at 31°C, 34°C, 37°C, or 39°C for 3 days. Virus plaques and MDCK cells were visualized by immunostaining using anti-NP antibody and CellTag700, respectively, and were detected with the Odyssey CLx Infrared Imaging System.

Supplementary Figure 4

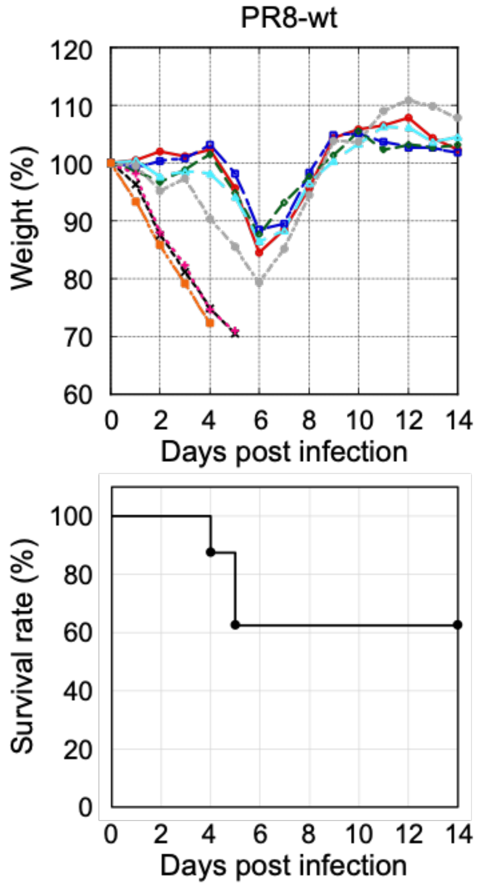

Supplementary Figure 4. Body weight change and survival rate of mice infected with 10^6 PFU/mouse of wild-type PR8 virus.

Six-week-old female C57BL/6J mice were intranasally inoculated with 10^6 PFU of PR8-wt (n = 8). Body weight (top panels) and survival (bottom panels) were monitored for 14 days after inoculation. The body weights of animals inoculated with virus is depicted as a percentage of the body weight compared with that on day 0.

Supplementary Figure 5

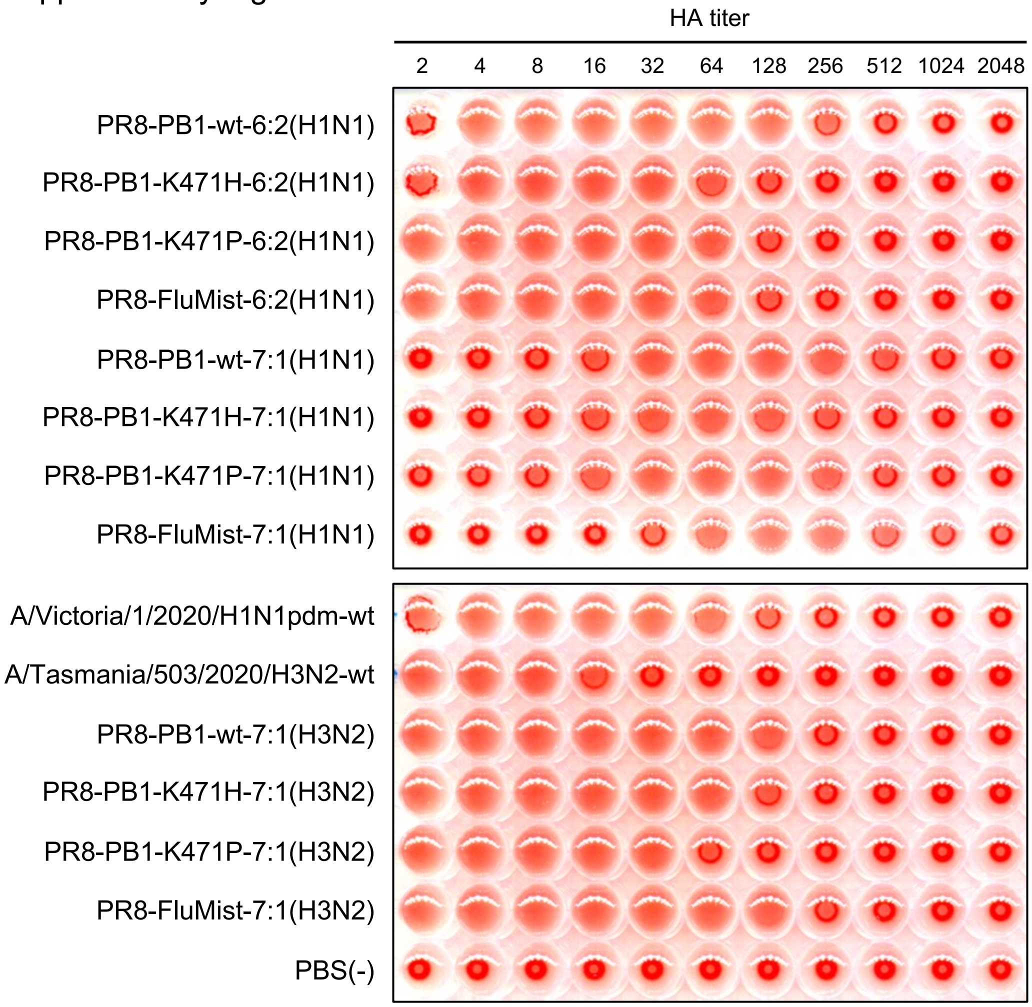

Supplementary Figure 5. Generation of 6:2 or 7:1 reassortant PR8-PB1-K471P-based vaccine viruses.

HA titers were determined using 6:2 or 7:1 reassortant viruses amplified in egg that were infected with 200 PFU of seed viruses generated by RG system. The HA assays were carried out with 1% guinea pig red blood cells.

Supplementary Tables

Supplementary Table 1: Nucleotide and amino acid substitutions detected in the PR8-FluMist virus showing loss of temperature-sensitive phenotype

Test virus	Expt no.[a]	Substitution(s) in:		
		PB1	PB2	PA
PR8-FluMist	1	G1171A(E391K)[b], G1742A(G581D)[b]	None	None
	2	G1171A(E391K)[b]	None	A1667A/G(Q556Q/R)
	3	G1171A(E391K)[b]	None	G7G/A(D3D/N)
	4	G1171A(E391K)[b]	G209G/A(R70R/K), G2101G/A(D701D/N)	G1129G/A(E377E/K)

[a]Data represent results from there four independent virus serial passages.

[b]Amino acid substitutions responsible for the temperature-sensitive phenotype.

Supplementary Table 2: HA assay of PR8-based 6:2 and 7:1 vaccine virus strain

Test virus	HA titer of E1 viruses[a]
A/Victoria/1/2020/H1N1-wt	64
PR8-PB1-wt-6:2(H1N1)	128
PR8-PB1-K471H-6:2(H1N1)	32
PR8-PB1-K471P-6:2(H1N1)	64
PR8-FluMist-6:2(H1N1)	64
PR8-PB1-wt-7:1(H1N1)	256
PR8-PB1-K471H-7:1(H1N1)	128
PR8-PB1-K471P-7:1(H1N1)	256
PR8-FluMist-7:1(H1N1)	256
A/Tasmania/503/2020/H3N2-wt	8
PR8-PB1-wt-7:1(H3N2)	128
PR8-PB1-K471H-7:1(H3N2)	64
PR8-PB1-K471P-7:1(H3N2)	32
PR8-FluMist-7:1(H3N2)	128

[a]HA titers of viruses isolated from E1 eggs were determined by HA assay with 1% guinea pig red blood cells. The values of HA titer were obtained from data of **Fig. S5**.

Supplementary Table 3: Primers used in this study

Primer name	Sequence (5' to 3')[a]
PB1-for	ACGACCggtaccGCCACCACCATGGATGTCAATCCGACCTTACTTTTC
PB1-rev	ATGCATGCgcggccgcCTATTTTTGCCGTCTGAGCTCTTCAAT
K471H-for	TTATCGAACCTGTCACCTACTTGGAATCAA
K471H-rev	TTGATTCCAAGTAGGTGACAGGTTCGATAA
K471R-for	TTATCGAACCTGTAGACTACTTGGAATCAA
K471R-rev	TTGATTCCAAGTAGTCTACAGGTTCGATAA
K471A-for	TTATCGAACCTGTGCGCTACTTGGAATCAA
K471A-rev	TTGATTCCAAGTAGCGCACAGGTTCGATAA
K471V-for	TTATCGAACCTGTGTGCTACTTGGAATCAA
K471V-rev	TTGATTCCAAGTAGCACACAGGTTCGATAA
K471I-for	TTATCGAACCTGTATACTACTTGGAATCAA
K471I-rev	TTGATTCCAAGTAGTATACAGGTTCGATAA
K471L-for	TTATCGAACCTGTCTGCTACTTGGAATCAA
K471L-rev	TTGATTCCAAGTAGCAGACAGGTTCGATAA
K471M-for	TTATCGAACCTGTATGCTACTTGGAATCAA
K471M-rev	TTGATTCCAAGTAGCATACAGGTTCGATAA
K471F-for	TTATCGAACCTGTTTCCTACTTGGAATCAA
K471F-rev	TTGATTCCAAGTAGGAAACAGGTTCGATAA
K471Y-for	TTATCGAACCTGTTATCTACTTGGAATCAA
K471Y-rev	TTGATTCCAAGTAGATAACAGGTTCGATAA
K471C-for	TTATCGAACCTGTTGTCTACTTGGAATCAA
K471C-rev	TTGATTCCAAGTAGACAACAGGTTCGATAA
K471G-for	TTATCGAACCTGTGGGCTACTTGGAATCAA
K471G-rev	TTGATTCCAAGTAGCCCACAGGTTCGATAA
K471P-for	TTATCGAACCTGTCCGCTACTTGGAATCAA
K471P-rev	TTGATTCCAAGTAGCGGACAGGTTCGATAA
K471S-for	TTATCGAACCTGTAGTCTACTTGGAATCAA
K471S-rev	TTGATTCCAAGTAGACTACAGGTTCGATAA
K471T-for	TTATCGAACCTGTACGCTACTTGGAATCAA

K471T-rev	TTGATTCCAAGTAGCGTACAGGTTCGATAA
K471N-for	TTATCGAACCTGTAACCTACTTGGAATCAA
K471N-rev	TTGATTCCAAGTAGGTTACAGGTTCGATAA
K471Q-for	TTATCGAACCTGTCAGCTACTTGGAATCAA
K471Q-rev	TTGATTCCAAGTAGCTGACAGGTTCGATAA
K471D-for	TTATCGAACCTGTGATCTACTTGGAATCAA
K471D-rev	TTGATTCCAAGTAGATCACAGGTTCGATAA
K471E-for	TTATCGAACCTGTGAGCTACTTGGAATCAA
K471E-rev	TTGATTCCAAGTAGCTCACAGGTTCGATAA
K471W-for	TTATCGAACCTGTTGGCTACTTGGAATCAA
K471W-rev	TTGATTCCAAGTAGCCAACAGGTTCGATAA
Pol1-for	GTGTGTCCTGGGGTTGACCAGA
Pol1-rev	CATCGGTGATGTCGGCGATATAG
A349V-for	TCAAACAAAATGGTGAGACTGGGAAAA
A349V-rev	TTTTCCCAGTCTCACCATTTTGTTTGA
PB1-3mut-for-1	AAGAGTATGAAACTTAGAACTCAAATACC
PB1-3mut-for-2	TAGAAATCTCCACATTCCTGAAGTC
PB1-3mut-rev-1	GACTTCAGGAATGTGGAGATTTCTA
PB1-3mut-rev-2	GGTATTTGAGTTCTAAGTTTCATACTCTT
PB2-N256S-for	TTGCTGCTAGGAGCATAGTGAGAAG
PB2-N256S-rev	CTTCTCACTATGCTCCTAGCAGCAA
Pol1-BsaI-for	CGTATTGGTCTCAGGGAGCGAAAGCAGG
Pol1-BsaI-for-2	CGTATTGGTCTCAGGGAGCAAAAGCAGG
Pol1-BsaI-rev	CGATATGGTCTCGTATTAGTAGAAACAAGG
Pol1-BsmB-for	CGTATTCGTCTCAGGGAGCAAAAGCAGG
Pol1-BsmB-for-2	CGTATTCGTCTCAGGGAGCGAAAGCAGG
Pol1-BsmB-rev	CGATATCGTCTCGTATTAGTAGAAACAAGG
Vic/H1N1-PB1-750-rev	TTTAACTTGCCTCTCTCTGCATCT
Vic/H1N1-PB1-750-for	AGATGCAGAGAGAGGCAAGTTAAA
Vic/H1N1-PB1-1468-rev	GTTGATTCCCACTAATTTGCAGGTC
Vic/H1N1-PB1-1468-for	GACCTGCAAATTAGTGGGAATCAAC
Vic/H1N1-PB2-732-rev	TCAATATAAACACTGCTTGTCCCAC

Vic/H1N1-PB2-732-for	GTGGGACAAGCAGTGTTTATATTGA
Vic/H1N1-PB2-1447-rev	GGTCATGTCGGGCAGTATTC
Vic/H1N1-PB2-1447-for	GAATACTGCCCGACATGACC
Vic/H1N1-PA-724-rev	GTTTTCAAGGCAGGAGAAGTTTGGT
Vic/H1N1-PA-724-for	ACCAAACTTCTCCTGCCTTGAAAAC
Vic/H1N1-PA-1433-rev	TGTACACTCCCTTCATTATGTATTCAG
Vic/H1N1-PA-1433-for	CTGAATACATAATGAAGGGAGTGTACA
Vic/H1N1-HA-889-rev	CAGATCCAGCATCTCTTTCCATTG
Vic/H1N1-HA-889-for	CAATGGAAAGAGATGCTGGATCTG
Vic/H1N1-NP-712-rev	AACTCTTGTCCTTCGTCCATTTTC
Vic/H1N1-NP-712-for	GAAAATGGACGAAGGACAAGAGTT
Vic/H1N1-NA-636-rev	ACTGTCTGGGCCAGAAATTCCAATT
Vic/H1N1-NA-636-for	AATTGGAATTTCTGGCCCAGACAGT
Vic/H1N1-MP-446-rev	CATCCTGTTGTATATGAGGCCCAT
Vic/H1N1-MP-446-for	ATGGGCCTCATATACAACAGGATG
Vic/H1N1-NS-455-rev	AAGATTACACTGAAGTTTGCTTCCAGTA
Vic/H1N1-NS-455-for	TACTGGAAGCAAACTTCAGTGTAATCTT
Tasm/H3N2-PB1-702-rev	GCTCTTATTAGGTAGCCTCTCTTATT
Tasm/H3N2-PB1-702-for	AATAAGAGAGGCTACCTAATAAGAGC
Tasm/H3N2-PB1-1405-rev	ATTTGGTGCATTCACTATGAGGGCA
Tasm/H3N2-PB1-1405-for	TGCCCTCATAGTGAATGCACCAAAT
Tasm/H3N2-PB2-713-rev	TTCCGCCAGCAACTGGGAGAAA
Tasm/H3N2-PB2-713-for	TTTCTCCCAGTTGCTGGCGGAA
Tasm/H3N2-PB2-1420-rev	TCCCATCACACTGTCGATGTGTT
Tasm/H3N2-PB2-1420-for	AACACATCGACAGTGTGATGGGA
Tasm/H3N2-PA-710-rev	AGAAGTTCGGTGGGAGACTTTG
Tasm/H3N2-PA-710-for	CAAAGTCTCCCACCGAACTTCT
Tasm/H3N2-PA-1424-rev	CTTTCATTATGTATTCAGTAGCTCTACAAT
Tasm/H3N2-PA-1424-for	ATTGTAGAGCTACTGAATACATAATGAAAG
Tasm/H3N2-HA-957-rev	AATGCTTCCATTTGGAGTGATGCATT
Tasm/H3N2-HA-957-for	AATGCATCACTCCAAATGGAAGCATT
Tasm/H3N2-NP-713-rev	CACTTCTTGTTTTCCGCCCATTCT

Tasm/H3N2-NP-713-for	AGAATGGGCGGAAAACAAGAAGTG
Tasm/H3N2-NA-641-rev	AGTTGCATTTTTATCATCCCCCGTT
Tasm/H3N2-NA-641-for	AACGGGGGATGATAAAAATGCAACT
Tasm/H3N2-MP-476-rev	GCCAAATGCCACTTCAGTGGTT
Tasm/H3N2-MP-476-for	AACCACTGAAGTGGCATTTGGC
Tasm/H3N2-NS-474-rev	TATGGTCTCTAGCCGGCCAAA
Tasm/H3N2-NS-474-for	TTTGGCCGGCTAGAGACCATA
Vic/H1N1-HA-PR8utr-for	AGCAAAAGCAGGGGAAAATAAAAACAACCAAAATGAAGGCAA TACTAGTAGTTATGCT
Vic/H1N1-HA-PR8utr-rev	AGTAGAAACAAGGGTGTTTTTCCTCATATTTCTGAAATTCTAA TCTCAAATACATATTCTACACTGTAGAGAC
Vic/H1N1-NA-PR8utr-for	AGCGAAAGCAGGGGTTTAAAATGAATCCAAACCAAAAGATAA TAAC
Vic/H1N1-NA-PR8utr-rev	AGTAGAAACAAGGAGTTTTTTGAACAGACTACTTGTCAATGGT AAATGGCAACT
Tasm/H3N2-HA-PR8utr-for	AGCAAAAGCAGGGGAAAATAAAAACAACCAAAATGAAGACTA TCATTGCTTTGAGCT
Tasm/H3N2-HA-PR8utr-rev	AGTAGAAACAAGGGTGTTTTTCCTCATATTTCTGAAATTCTAA TCTCAAATGCAAATGTTGCATCTAATGTTG
Tasm/H3N2-NA-PR8utr-for	AGCGAAAGCAGGGGTTTAAAATGAATCCAAATCAAAAGATAA TAACGA
Tasm/H3N2-NA-PR8utr-rev	AGTAGAAACAAGGAGTTTTTTGAACAGACTATATATGCATGAG ACTGAGGTTC

[a]Underlining indicates the codon sequence corresponding to amino acid 471 of the PB1 protein.

Supplementary Table 4: Synthetic DNA fragments used in this study

DNA fragment name	Length	Sequence (5' to 3')
PR8-PB1-K391E/E581G	750 bp	AAGAGTATGAAACTTAGAACTCAAATACCTGCAGAAATGCTAGCAAGCATCGATT<br>TGAAATATTTCAATGATTCAACAAGAAAGAAGATTGAAGAGATCCGACCGCTCTT<br>AATAGAGGGGACTGCATCATTGAGCCCTGGAATGATGATGGGCATGTTCAATAT<br>GTTAAGCACTGTATTAGGCGTCTCCATCCTGAATCTTGGACAAAAGAGATACACC<br>AAGACTACTTACTGGTGGGATGGTCTTCAATCCTCTGACGATTTTGCTCTGATTG<br>TGAATGCACCCAATCATGAAGGGATTCAAGCCGGAGTCGACAGGTTTTATCGAA<br>CCTGTAAGCTACTTGGAATCAATATGAGCAAGAAAAAGTCTTACATAAACAGAAC<br>AGGTACATTTGAATTCACAAGTTTTTTCTATCGTTATGGGTTTGTTGCCAATTTCA<br>GCATGGAGCTTCCCAGTTTTGGGGTGTCTGGGATCAACGAGTCAGCGGACATGA<br>GTATTGGAGTTACTGTCATCAAAAACAATATGATAAACAATGATCTTGGTCCAGC<br>AACAGCTCAAATGGCCCTTCAGTTGTTCATCAAAGATTACAGGTACACGTACCGA<br>TGCCATATAGGTGACACACAAATACAAACCCGAAGATCATTTGAAATAAAGAAAC<br>TGTGGGGTCAAACCCGTTCCAAAGCTGGACTGCTGGTCTCCGACGGAGGCCCA<br>AATTTATACAACATTAGAAATCTCCACATTCCTGAAGTC
PR8-PB1-A661T	698 bp	TAGAAATCTCCACATTCCTGAAGTCTGCCTAAAATGGGAATTGATGGATGAGGAT<br>TACCAGGGGCGTTTATGCAACCCACTGAACCCATTTGTCAGCCATAAAGAAATTG<br>AATCAATGAACAATGCAGTGATGATGCCAGCACATGGTCCAGCCAAAAACATGG<br>AGTATGATGCTGTTACTACAACACACTCCTGGATCCCCAAAAGAAATCGATCCAT<br>CTTGAATACAAGTCAAAGAGGAGTACTTGAGGATGAACAAATGTACCAAAGGTG<br>CTGCAATTTATTTGAAAAATTCTTCCCCAGCAGTTCATACAGAAGACCAGTCGGG<br>ATATCCAGTATGGTGGAGGCTATGGTTTCCAGAGCCCGAATTGATGCACGGATT<br>GATTTCGAATCTGGAAGGATAAAGAAAGAAGAGTTCACTGAGATCATGAAGATCT<br>GTTCCACCATTGAAGAGCTCAGACGGCAAAAATAGTGAATTTAGCTTGTCCTTCA<br>TGAAAAAATGCCTTGTTTCTACTAATAACCCGGCGGCCCAAAATGCCGACTCGG<br>AGCGAAAGATATACCTCCCCCGGGGCCGGGAGGTCGCGTCACCGACCACGCC<br>GCCGGCCCAGGCGACGCGCGACACGGACACCTGTCCCCAAAAACGCCACCATC<br>GCAGCCACACACGGAGCGCCCGGGGCCCTCTGGTCAACCCCAGGACA

Victoria/H1N1-PB1-5’	750 bp	CGTATTCGTCTCAGGGAGCGAAAGCAGGCAAACCATTTGAATGGATGTCAATCC<br>GACTCTACTTTTCCTAAAAATTCCAGCACAAAATGCCATAAGCACCACATTCCCTT<br>ATACTGGAGATCCTCCATACAGCCATGGAACAGGAACAGGATACACCATGGACA<br>CAGTAAACAGAACACACCAATACTCAGAAAAGGGGAAGTGGACAACAAACACAG<br>AAACTGGTGCACCCCAGCTCAACCCGATTGACGGACCACTACCCGAAGATAATG<br>AACCAAGTGGGTATGCACAAACAGACTGTGTTCTAGAGGCTATGGCTTTCCTTGA<br>AGAATCCCATCCAGGAATATTTGAAAATTCATGCCTTGAAACAATGGAAGTTGTT<br>CAACAAACAAGGGTAGATAAACTGACTCAAGGTCGCCAGACTTATGATTGGACAT<br>TAAACAGAAATCAACCGGCAGCAACTGCATTGGCCAACACCATAGAAGTTTTCAG<br>ATCGAATGACCTAACAGCTAACGAGTCAGGAAGGCTAATAGATTTCTTAAAGGAT<br>GTGATGGAATCAGTGAACAAAGAGGAAATAGAGATAACAACCCACTTTCAAAGAA<br>AAAGGAGAGTAAGAGACAACATGACCAAGAAGATGATCACGCAAAGAACAATAG<br>GGAAGAAAAAACAGAGACTGAATAAGAGAAGCTATCTAATAAGAGCACTGACATT<br>AAATACGATGACCAAAGATGCAGAGAGAGGCAAGTTAAA
Victoria/H1N1-PB1-middle	742 bp	AGATGCAGAGAGAGGCAAGTTAAAAAGAAGGGCTATCGCAACACCTGGGATGCA<br>GATTAGAGGTTTCGTATACTTTGTTGAAACTTTAGCTAGGAGCATTTGCGAAAAG<br>CTTGAACAGTCTGGGCTCCCAGTAGGGGGCAATGAAAAGAAGGCCAAATTGGCA<br>AATGTTGTGAGAAAGATGATGACTAATTCACAAGATACAGAGATTTCTTTCACAAT<br>CACTGGGGACAACACTAAATGGAATGAGAATCAAAATCCTCGAATGTTCCTGGC<br>GATGATTACATATATCACCAGAAATCAACCCGAGTGGTTTAGAAACATCCTGAGC<br>ATGGCACCCATAATGTTCTCAAATAAAATGGCAAGGCTAGGAAAAGGGTACATGT<br>TCGAGAGTAAAAGAATGAAGATTCGAACACAAATACCAGCAGAAATGCTAGCAA<br>GCATTGATCTAAAGTATTTCAATGAATCAACAAGGAAGAAAATTGAGAAGATAAG<br>GCCTCTTTTAATGGATGGCACAGCATCACTGAGTCCTGGGATGATGATGGGCAT<br>GTTCAACATGCTAAGTACGGTCTTGGGAGTCTCGATACTGAATCTTGGACAAAAG<br>AAATACACCAAGACAACATACTGGTGGGATGGGCTCCAATCATCCGACGATTTT<br>GCTCTCATAGTGAATGCACCAAACCATGAAGGAATACAAGCAGGAGTGGACAGA<br>TTTTACAGGACCTGCAAATTAGTGGGAATCAAC

Victoria/H1N1-PB1-3’	931 bp	GACCTGCAAATTAGTGGGAATCAACATGAGCAAAAAGAAGTCCTATATAAATAAG<br>ACAGGGACATTTGAATTCACAAGCTTTTTTTATCGCTATGGATTTGTGGCTAATTT<br>TAGCATGGAGCTACCCAGCTTTGGAGTGTCTGGAGTAAATGAATCAGCTGACAT<br>GAGTATTGGAGTAACAGTGATAAAGAACAACATGATAAACAATGACCTTGGACCT<br>GCAACGGCTCAGATGGCTCTTCAATTGTTCATAAAAGACTACAGATACACATATA<br>GGTGTCATAGGGGAGACACACAAATTCAGACAAGAAGATCATTTGAGTTGAAGA<br>AGCTATGGGATCAAACCCAATCAAAGGTAGGGCTATTAGTATCAGATGGAGGAC<br>CAAACTTATACAACATACGGAATCTTCACATTCCTGAAGTCTGCTTAAAATGGGA<br>GCTAATGGATGATGATTATCGGGGAAGACTTTGTAATCCCCTGAATCCCTTTGTG<br>AGTCATAAGGAGATTGATTCTGTAAACAATGCTGTGGTAATGCCAGCCCATGGC<br>CCAGCCAAAAGCATGGAATATGATGCCGTCGCTACTACACATTCCTGGATTCCC<br>AAGAGGAATCGTTCTATTCTCAACACAAGCCAAAGGGGAATTCTTGAGGATGAAC<br>AGATGTACCAGAAGTGTTGCAATTTATTCGAGAAATTTTTCCCTAGCAGTTCATAT<br>AGGAGACCGGTTGGAATTTCTAGCATGGTGGAGGCCATGGTGTCTAGGGCCCG<br>GATTGATGCCAGAGTCGACTTTGAGTCTGGACGGATTAAGAAAGAAGAGTTCTC<br>TGAGATCATGAAGATCTGTTCCACCATTGAAGAACTCAGACGGCAAAAATAATGA<br>ATTTAGCTTGTCCTTCATGAAAAAATGCCTTGTTTCTACTAATACGAGACGATATC<br>G
Victoria/H1N1-PB2-5’	732 bp	CGTATTGGTCTCAGGGAGCGAAAGCAGGTCAATTATATTCAATATGGAGAGAATA<br>AAAGAGCTGAGAGATCTAATGTCGCAGTCCCGCACTCGCGAGATACTCACTAAG<br>ACCACTGTGGACCATATGGCCATCATCAAAAAGTACACATCGGGAAGGCAAGAG<br>AAGAACCCCGCGCTCAGAATGAAGTGGATGATGGCAATGAAATACCCAATTACG<br>GCAGACAAGAGAATAATGGACATAATCCCAGAGAGGAATGAACAAGGACAAACC<br>CTCTGGAGCAAAATAAACGATGCTGGATCAGACCGAGTGATGGTATCACCTCTG<br>GCAGTAACATGGTGGAATAGGAATGGTCCAACAACAAGTACAGTTCATTACCCTA<br>AGGTATATAAAACTTATTTCGAAAAGGTCGAAAGGTTGAAACATGGTACCTTCGG<br>CCCTGTCCACTTCAGAAATCAAGTTAAAATAAGGAGGAGAGTTGATACAAACCCT<br>GGCCATGCAGATCTCAGTGCCAAGGAGGCACAGGATGTGATTATGGAAGTTGTT<br>TTCCCAAATGAAGTGGGGGCAAGAATACTGACATCAGAGTCACAGCTGGCAATA<br>ACAAAAGAGAAGAAAGAAGAGCTCCAGAATTGTAAAATTGCTCCCTTGATGGTG<br>GCGTACATGCTAGAAAGAGAATTGGTCCGTAAAACAAGGTTTCTCCCAATAGCC<br>GGTGGGACAAGCAGTGTTTATATTGA

Victoria/H1N1-PB2-middle	740 bp	GTGGGACAAGCAGTGTTTATATTGAGGTGTTGCACTTGACCCAAGGGACGTGCT GGGAGCAGATGTACACTCCAGGAGGAGAAGTGAGAAATGATGATGTTGACCAAA GCTTGATTATCGCTGCTAGAAACATAGTAAGAAGAGCAGCAGTGTCAGCAGACC CATTAGCATCTCTCTTGGAAATGTGCCACAGCACACAGATTGGAGGTGTGAAGA TGGTGGACATCCTTAAACAGAATCCAACTGAGGAGCAAGCCGTAGACATATGCA AGGCAGCAATAGGGTTGAGGATCAGCTCATCTTTCAGTTTTGGTGGGTTCACTTT CAAAAGGACAAGCGGATCATCAGTCAAGAAAGAAGAAGAAATGCTAACGGGCAA CCTCCAAACACTGAAATTAAGAGTACATGAAGGGTATGAAGAATTCACAATGGTT GGGAGAAGAGCAACAGCTATTCTCAGAAAGGCAACCAGGAGATTGATCCAATTA ATAGTAAGCGGGAGAGACGAGCAGTCAATAGCTGAAGCAATAATTGTGGCCATG GTTTTCTCACAAGAGGATTGCATGATCAAAGCAGTTAGGGGCGATCTGAACTTTG TCAATAGGGCAAACCAGAGACTGAATCCCATGCACCAACTCTTGAGGCATTTCC AAAAAGATGCAAAGGTGCTTTTCCAGAACTGGGGAATTGAAACCATCGACAATGT GATGGGAATGATCGGAATACTGCCCGACATGACC
Victoria/H1N1-PB2-3’	947 bp	GAATACTGCCCGACATGACCCCAAGCACGGAGATGTCACTGAGAGGAATAAGAG TCAGCAAGATGGGAGTAGATGAATACTCCAGCACGGAGAGAGTGGTAGTGAGTA TTGACCGATTTTTGAGGGTTAGAGATCAAAGAGGAAACGTACTATTGTCTCCCGA AGAAGTCAGTGAAACGCAAGGAACTGAGAAGTTGACAATAACTTATTCGTCATCA ATGATGTGGGAAATCAACGGCCCTGAGTCAGTGCTAGTCAACACTTATCAATGG ATAATCAGAAACTGGGAAATTGTGAAAATTCAATGGTCACAAGACCCCACAATGT TATACAACAAAATGGAATTTGAACCATTTCAGTCTCTTGTTCCTAAGGCAACCAGA AGCCGGTACAGTGGATTCGTAAGGACACTGTTCCAGCAAATGAGGGATGTGCTT GGGACATTTGACACTGTCCAAATAATAAAACTTCTCCCCTTTGCTGCTGCTCCAC CAGAACAGAGCAGGATGCAATTTTCTTCATTGACTGTGAATGTGAGAGGATCAG GGTTAAGGATACTGGTAAGAGGCAATTCTCCAGTATTCAATTACAACAAGGCAAC CAAACGACTTACGATTCTTGGAAAGGATGCAGGTGCATTGACTGAAGATCCAGA TGAAGGCACATCTGGGGTGGAGTCTGCTGTCCTGAGGGGATTCCTCATTTTAGG CAAAGAAGACAAGAGATATGGCCCAGCATTAAGCATCAATGAACTGAGCAATCTT GCAAAAGGAGAGAAAGCTAATGTGCTAATTGGGCAGGGGGACATAGTGTTGGTA ATGAAACGAAAACGGGACTCTAGCATACTTACTGACAGCCAGACAGCGACCAAA AGAATTCGAATGGCCATCAATTAGTGTCGAATAGTTTAAAAACGACCTTGTTTCTA CTAATACGAGACCATATCG

Victoria/H1N1-PA-5’	724 bp	CGTATTCGTCTCAGGGAGCGAAAGCAGGTACTGATCCAAAATGGAAGACTTTGT<br>GCGACAATGCTTCAATCCAATGATCGTCGAGCTTGCGGAAAAGGCAATGAAAGA<br>ATATGGGGAAGACCCCAAAATTGAAACTAATAAGTTTGCTGCAATTTGCACACAT<br>TTGGAAGTTTGTTTCATGTATTCGGATTTCCATTTCATTGATGAACGGGGTGAATC<br>AATAATTTTAGAATCTGGTGACCCAAATGCACTATTGAAGCACCGATTTGAGATA<br>ATTGAAGGAAGAGACCGAATCATGGCCTGGACAGTGGTGAACAGTATATGTAAC<br>ACAACAGGGATAGAGAAGCCTAAATTTCTTCCTGATTTGTATGATTACAAAGAGA<br>ACCGGTTCATTGAAATTGGAGTAACACGGAGAGAAGTCCACATATATTACCTAGA<br>GAAAGCCAACAAAATAAAATCTGAGAAGACACACATTCACATCTTTTCATTCACTG<br>GAGAGGAGATGGCCACCAAAGCGGACTACACCCTTGACGAAGAAAGCAGAGCA<br>AGAATCAAAACTAGGCTTTTCACTATAAGACAAGAAATGGCCAGTAGGAGTCTAT<br>GGGATTCCTTTCGTCAGTCCGAAAGAGGCGAAGAAACAATTGAAGAAAAGTTTG<br>AAATTACAGGAACTATGCGCAAGCTTGCCGACCAAAGTCTCCCACCAAACTTCTC<br>CTGCCTTGAAAAC
Victoria/H1N1-PA-middle	734 bp	ACCAAACTTCTCCTGCCTTGAAAACTTCAGAGCCTATGTAGATGGATTCGAGCCG<br>AACGGCTGCATTGAGGGCAAGCTTTCCCAAATGTCAAAAGAAGTGAACGCCAAA<br>ATTGAACCATTCTTGAGGACGACACCACGCCCCCTCAGATTACCTGATGGACCT<br>TTTTGCCATCAGCGGTCAAAGTTCCTGCTGATGGATGCTCTGAAATTAAGCATTG<br>AAGACCCGAGTCACGAGGGGGAAGGAATACCACTATATGATGCAATCAAATGCA<br>TGAAGACATTCTTTGGCTGGAAAGAGCCTAAAATAGTCAAACCACATGAGAAAGG<br>CGTAAATCCCAATTACCTCATGGCTTGGAAGCAGGTGCTAGCAGAGCTACAGGA<br>CATTGAGAATGAAGAGAAAATCCCAAGGACAAAGAACATGAAGAAAACAAGCCA<br>ATTGAAGTGGGCACTCGGTGAAAATATGGCACCTGAAAAAGTGGACTTTGATGA<br>CTGTAAAGATGTTGGAGACCTTAAACAGTATGACAGTGATGAGCCAGAGCCCAG<br>ATCTCTAGCAAGCTGGGTCCAAAATGAATTCAATAAGGCATGTGAATTGACTGAT<br>TCAAGCTGGATAGAACTTGATGAAATAGGAGAAGATGTTGCCCCGATTGAACATA<br>TAGCAAGCATGAGGAGGAACTATTTCACAGCAGAAGTGTCCCACTGCAGGGCTA<br>CTGAATACATAATGAAGGGAGTGTACA

Victoria/H1N1-PA-3’	860 bp	CTGAATACATAATGAAGGGAGTGTACATAAATACGGCCTTGCTTAATGCATCCTG<br>TGCAGCCATGGATGACTTTCAGTTGATTCCAATGATAAGCAAATGTAGGACCAAA<br>GAAGGAAGACGGAAAACAAACCTGTATGGGTTCATTGTAAAAGGAAGGTCTCAT<br>TTGAGAAATGATACTGATGTAGTGAACTTTGTAAGTATGGAGTTCTCACTCACTG<br>ATCCAAGACTGGAACCACACAAATGGGAAAAATACTGTGTTCTTGAAATAGGAGA<br>CATGCTATTGAGGACTGCGATAGGTCAAGTGTCGAGGCCCATGTTCCTATATGT<br>GAGAACCAATGGAACCTCCAAGATCAAGATGAAATGGGGCATGGAAATGAGGCG<br>CTGCCTTCTTCAATCTCTTCAACAGATTGAGAGCATGATTGAAGCCGAGTCTTCT<br>GTCAAAGAGAAAGACATGACCAAGGAATTCTTTGAAAACAAATCGGAAACATGGC<br>CAATCGGAGAGTCACCCAGGGGAGTGGAGGAAGGCTCTATTGGAAAAGTGTGC<br>AGAACCTTACTGGCAAAATCTGTGTTCAACAGTCTATATGCTTCTCCACAACTTG<br>AGGGGTTTTCGGCTGAATCAAGAAAATTGCTTCTCATTGTTCAGGCACTTAGGGA<br>CAACCTGGAACCTGGAACCTTTGATCTTGGGGGGCTATATGAAGCAATCGAGGA<br>GTGCTTGATTAATGATCCCTGGGTTTTGCTTAATGCATCTTGGTTCAACTCCTTCC<br>TCACACATGCACTGAAGTAGTTGTGGCAATGCTACTATTTGCTATCCATACTGTC<br>CAAAAAAGTACCTTGTTTCTACTAATACGAGACGATATCG
Victoria/H1N1-HA-5’	889 bp	CGTATTCGTCTCAGGGAGCAAAAGCAGGGGAAAACAAAAGCAACAAAAATGAAG<br>GCAATACTAGTAGTTATGCTGTATACATTTACAACCGCAAATGCAGACACATTAT<br>GCATAGGTTATCATGCGAACAATTCAACAGACACTGTGGACACAGTACTAGAAAA<br>GAATGTAACAGTAACACACTCTGTCAATCTTCTGGAAGACAAGCATAACGGAAAA<br>CTATGCAAACTAAGAGGGGTAGCCCCATTGCATTTGGGTAAATGTAACATTGCTG<br>GCTGGATCCTGGGAAATCCAGAGTGTGAATCACTCTCCACAGCAAGATCATGGT<br>CCTACATTGTGGAAACATCTAATTCAGACAATGGAACGTGTTACCCAGGAGATTT<br>CATCAATTATGAGGAGCTAAGAGAGCAATTGAGCTCAGTGTCATCATTTAAAAGG<br>TTTGAAATATTCCCCAAGACAAGTTCATGGCCTAATCATGACTCGGACAATGGTG<br>TAACGGCAGCATGTCCTCACGCTGGAGCAAAAAGCTTCTACAAAAACTTGATATG<br>GCTGGTTAAAAAAGGAAAATCATACCCAAAGATCAACCAAACCTACATTAATGAT<br>AAAGGGAAAGAAGTCCTCGTGCTGTGGGGCATTCACCATCCACCTACTATTGCT<br>GACCAACAAAGTCTCTATCAGAATGCAGATGCATATGTTTTTGTGGGGACATCAA<br>GATACAGCAAGATGTTCAAGCCGGAAATAGCAACAAGACCCAAAGTGAGGGATC<br>GAGAAGGGAGAATGAACTATTACTGGACACTAGTAGAACCGGGAGACAAAATAA<br>CATTCGAAGCAACTGGTAATCTAGTGGCACCGAGATATGCATTCACAATGGAAA<br>GAGATGCTGGAT

Victoria/H1N1-HA-3’	946 bp	CAATGGAAAGAGATGCTGGATCTGGTATTATCATTTCAGATACACCAGTCCACGA<br>TTGCAATACAACTTGTCAAACACCCGAGGGTGCTATAAACACCAGCCTCCCATTT<br>CAGAATGTACATCCGATCACAATTGGGAAATGTCCAAAGTATGTAAAAAGCACAA<br>AATTGAGACTGGCCACAGGATTGAGGAATGTCCCGTCTATTCAATCTAGAGGCC<br>TATTCGGGGCCATTGCTGGCTTCATCGAAGGGGGGTGGACAGGGATGGTAGAT<br>GGATGGTACGGTTATCACCATCAAAATGAGCAGGGGTCAGGATATGCAGCCGAT<br>CTGAAGAGCACACAAAATGCCATTGATAAGATTACTAACAAAGTAAATTCTGTTAT<br>TGAAAAGATGAATACACAGTTCACAGCAGTTGGTAAAGAGTTCAACCACCTTGAA<br>AAAAGAATAGAGAATCTAAATAAAAAGGTTGATGATGGTTTCCTGGACATTTGGA<br>CTTACAATGCCGAACTGTTGGTTCTACTGGAAAACGAAAGAACTTTGGACTATCA<br>CGATTCAAATGTGAAGAACTTGTATGAAAAAGTAAGAAACCAGTTAAAAAACAAT<br>GCCAAGGAAATTGGAAACGGCTGCTTTGAATTTTACCACAAATGCGACAACACAT<br>GCATGGAAAGTGTCAAGAATGGGACTTATGACTACCCAAAATACTCAGAGGAAG<br>CAAAATTAAACAGAGAAAAAATAGATGGAGTAAAGCTGGACTCAACAAGGATCTA<br>CCAGATTTTGGCGATCTATTCAACTGTTGCCAGTTCATTGGTACTGGTAGTCTCC<br>CTGGGGGCAATCAGCTTCTGGATGTGCTCTAATGGGTCTCTACAGTGTAGAATA<br>TGTATTTAACATTAGGATTTCAGAAATATGAGGAAAAACACCCTTGTTTCTACTAA<br>TACGAGACGATATCG
Victoria/H1N1-NP-5’	712 bp	CGTATTCGTCTCAGGGAGCAAAAGCAGGGTAGATAATCACTCAATGAGTGACAT<br>CGAAGCCATGGCGTCACAAGGCACCAAACGATCATATGAACAAATGGAGACTGG<br>TGGGGAGCGCCAGGATACCACAGAAATCAGAGCATCTGTTGGAAGAATGATTGG<br>TGGAATTGGGAGATTCTACATCCAAATGTGCACTGAACTAAAACTCAGTGATTAT<br>GATGGACGACTAATCCAGAACAGCATAACAATAGAGAGGATGGTGCTTTCTGCT<br>TTTGATGAGAGAAGAAATAAATACCTAGAAGAGCATCCAAGTGCTGGGAAGGAC<br>CCTAAGAAAACAGGAGGACCCATCTATAGAAGAATAGACGGAAAATGGACAAGA<br>GAACTCATCCTTTATGACAAAGAAGAAATAAGGAGAGTTTGGCGCCAAGCAAACA<br>ATGGCGAAGATGCAACAGCAGGTCTTACTCATATCATGATTTGGCATTCCAATCT<br>GAATGATGCCACATATCAGAGGACAAGAGCACTTGTTCGCACTGGAATGGATCC<br>CAGAATGTGCTCTCTAATGCAAGGTTCAACACTTCCCAGGAGGTCTGGTGCCGC<br>AGGTGCTGCAGTAAAAGGAGTTGGAACAATAGCTATGGAGTTAATCAGAATGATA<br>AAACGTGGAATCAATGACCGAAATTTCTGGAGGGGTGAAAATGGACGAAGGACA<br>AGAGTT

Victoria/H1N1-NP-3’	910 bp	GAAAATGGACGAAGGACAAGAGTTGCTTATGAAAGAATGTGCAATATCCTCAAAG<br>GAAAATTTCAAACAGCTGCCCAGAGGGCAATGATGGATCAAGTAAGGGAAAGTC<br>GAAACCCAGGAAACGCTGAGATTGAAGACCTCATTTTCCTGGCACGGTCAGCAC<br>TCATTCTGAGAGGATCAGTTGCACATAAATCCTGCCTGCCTGCTTGTGTGTATGG<br>GCTTGCAGTAGCAAGTGGCCATGACTTTGAAAGGGAAGGGTACTCATTGGTCGG<br>GATAGACCCATTCAAATTACTCCAAAACAGTCAAGTGGTCAGCCTGATGAGACCA<br>AATGAAAATCCAGCTCACAAGAGTCAATTGGTATGGATGGCATGCCACTCTGCT<br>GCATTTGAAGATTTAAGAGTATCAAGTTTCATAAGAGGAAAGAAAGTGATCCCAA<br>GAGGAAAGCTTTCCACAAGAGGGGTTCAGATTGCTTCAAATGAGAATGTAGAAG<br>CCATGGACTCCAATACCCTGGAACTAAGAAGCAGATACTGGGCCATAAGAACCA<br>GAAGTGGAGGAAATACCAATCAACAGAAGGCATCTGCAGGCCAGATCAGTGTGC<br>AGCCTACATTCTCAGTGCAGCGAAATCTCCCCTTTGAAAGAGCAACCATTATGGC<br>AGCATTCAGCGGGAACAATGAAGGACGGACATCCGACATGCGAACAGAAGTTAT<br>AAGAATGATGGAAAGTGCAAAGCCAGAGGATTTGTCCTTCCAGGGGCGGGGAG<br>TCTTCGAGCTCTCGGACGAAAAGGCAACGAACCCGATCGTGCCTTCCTTTGACA<br>TGAGTAATGAAGGGTCTTATTTCTTCGGAGACAATGCAGAGGAGTATGACAATTG<br>AAGAAAAATACCCTTGTTTCTACTAATACGAGACGATATCG
Victoria/H1N1-NA-5’	636 bp	CGTATTCGTCTCAGGGAGCGAAAGCAGGAGTTTAAAATGAATCCAAACCAAAAG<br>ATAATAACCATTGGTTCTATCTGTATGACAATTGGAACGGCTAACTTAATATTACA<br>AATTGGAAACATAATCTCAATATGGGTTAGCCACTCAATTCAAATTGGAAATCAAA<br>GCCAGATTGAAACATGCAATAAAAGCGTCATTACTTATGAAAACAACACTTGGGT<br>AAATCAGACATTTGTTAACATCAGCAACACTAACTCTGCTGCTAGACAGTCAGTG<br>GCTTCCGTGAAATTAGCGGGCAATTCCTCTCTCTGCCCTGTTAGTGGATGGGCT<br>ATATACAGTAAAGACAACAGTGTAAGAATCGGTTCCAAGGGGGATGTGTTTGTCA<br>TAAGGGAACCATTCATATCATGCTCTCCCTTGGAATGCAGAACCTTCTTCTTGAC<br>TCAAGGGGCTTTGCTGAATGACAAACATTCCAATGGAACCATTAAAGACAGAAGC<br>CCATATCGAACCCTAATGAGCTGTCCTATTGGTGAAGTTCCCTCTCCATACAACT<br>CAAGATTTGAGTCAGTCGCTTGGTCAGCAAGTGCTTGTCATGATGGCACCAATT<br>GGCTAACAATTGGAATTTCTGGCCCAGACAGT

Victoria/H1N1-NA-3’	880 bp	AATTGGAATTTCTGGCCCAGACAGTGGGGCAGTGGCTGTGTTAAAATACAATGG<br>CATAATAACAGACACTATCAAGAGTTGGAGGAACAAGATATTGAGAACACAAGAG<br>TCTGAATGTGCATGTGTAAATGGTTCTTGCTTTACCATAATGACCGATGGACCAA<br>GTGATGGACAGGCCTCATACAAAATCTTCAGAATAGAAAAGGGAAAGATAATCAA<br>ATCAGTCGAAATGAAAGCCCCTAATTATCACTATGAAGAATGCTCCTGTTACCCT<br>GATTCTAGTGAAATCACATGTGTGTGCAGGGATAACTGGCATGGCTCGAATCGA<br>CCGTGGGTGTCTTTCAACCAGAATCTGGAATATCAGATGGGATACATATGCAGT<br>GGGGTTTTCGGAGACAATCCACGCCCTAATGATAAGACAGGCAGTTGTGGTCCA<br>GTATCGTCTAATGGAGCAAATGGGGTAAAAGGATTTTCATTCAAATACGGCAATG<br>GTGTTTGGATAGGGAGAACTAAGAGCATTAGTTCAAGAAAAGGTTTTGAGATGAT<br>TTGGGATCCGAATGGATGGACTGGGACTGACAATAAATTCTCAAAAAAGCAAGAT<br>ATCGTAGGAATAAATGAGTGGTCAGGGTATAGCGGGAGTTTTGTTCAGCATCCA<br>GAACTAACAGGGCTGAATTGTATAAGACCTTGCTTCTGGGTTGAACTAATAAGAG<br>GACGACCCGAAGAGAACACAATCTGGACTAGCGGAAGCAGCATATCCTTTTGTG<br>GTGTAGACAGTGACATTGTGGGTTGGTCTTGGCCAGACGGTGCTGAGTTGCCAT<br>TTACCATTGACAAGTAATTTGTTCAAAAAACTCCTTGTTTCTACTAATACGAGACG<br>ATATCG
Victoria/H1N1-M-5’	446 bp	CGTATTCGTCTCAGGGAGCGAAAGCAGGTAGATGTTTAAAGATGAGTCTTCTAAC<br>CGAGGTCGAAACGTACGTTCTTTCTATCATCCCGTCAGGCCCCCTCAAAGCCGA<br>GATCGCACAGAGACTGGAAAGTGTCTTTGCAGGAAAGAACACAGATCTTGAGGC<br>TCTCATGGAATGGCTAAAGACAAGACCAATCTTGTCACCTCTGACTAAGGGAATT<br>TTAGGATTTGTGTTCACGCTCACCGTGCCCAGTGAGCGAGGACTGCAGCGTAGA<br>CGCTTTATCCAAAATGCCCTAAATGGAAATGGGGACCCGAACAACATGGATAGA<br>GCAGTTAAACTATACAAGAAACTCAAAAGAGAAATAACGTTCCATGGGGCCAAAG<br>AAGTGTCACTAAGCTATTCAACTGGTGCACTTGCAAGTTGCATGGGCCTCATATA<br>CAACAGGATG

Victoria/H1N1-M-3’	638 bp	ATGGGCCTCATATACAACAGGATGGGAACAGTGACCACAGAAGCTGCTTTCGGT<br>CTAGTTTGTGCCACTTGTGAACAGATTGCTGATTCACAGCATCGGTCTCACAGAC<br>AAATGGCTACTACCACAAATCCACTAATCAGGCATGAAAACAGAATGGTGCTGG<br>CTAGCACTACGGCAAAGGCTATGGAACAGGTGGCTGGATCGAGTGAACAGGCA<br>GCGGAGGCCATGGAGGTTGCTAATAAGACTAGGCAGATGGTACATGCAATGAGA<br>ACTATTGGAACTCATCCTAGCTCCAGTGCTGGTCTAAGAGATGACCTTCTTGAAA<br>ATTTACAGGCCTACCAGAAGCGAATGGGAGTGCAGATGCAGCGGTTCAAATGAT<br>CCTCTCGTCATTGCAGCAAACATCATTGGGATCTTGCACCTGATATTGTGGATTA<br>CTGATCGTCTTTTTTTCAAATGCATTTATCGTCGCTTTAAATACGGTTTGAAAAGA<br>GGGCCTTCTACGGAAGGAGTGCCTGAGTCCATGAGGGAAGAATATCAACAGGA<br>GCAGCAGAGTGCTGTGGATGTTGACGATGGTCATTTTGTCAACATAGAGCTAGA<br>GTAAAAAACTACCTTGTTTCTACTAATACGAGACGATATCG
Victoria/H1N1-NS-5’	455 bp	CGTATTCGTCTCAGGGAGCAAAAGCAGGGTGACAAAAACATAATGGAATCCAAC<br>ACCATGTCAAGCTTTCAGGTAGACTGTTTTCTTTGGCATATTCGCAAGCGATTTG<br>CAGACAATGGATTGGGTGATGCCCCATTCCTCGATCGGCTACGCCGAGATCAAA<br>AGTCCTTAAAAGGAAGAGGCAACACCCTTGGCCTCGACATCAAAACAGCCACTC<br>TTGTTGGGAAACTAATTGTGGAATGGATTTTGAAAGAGGAATCCAGCGAGACACT<br>TAGAATGGCAATTGCATCTGTACCTACTTCGCGTTACATTTCTGACATGACCCTC<br>GAGGAAATGTCACGAGACTGGTTCATGCTTATGCCTAGGCAAAAGATAATAGGC<br>CCTCTTTGCGTGCGATTGGACCAGGCGGTCATGGATAAGAACATAGTACTGGAA<br>GCAAACTTCAGTGTAATCTT
Victoria/H1N1-NS-3’	496 bp	TACTGGAAGCAAACTTCAGTGTAATCTTCAACCGATTAGAGACCTTGATACTACT<br>AAGGGCTTTCACTGAGGAGGGAACAATAGTTGGAGAAATTTCACCATTACCTTCT<br>CTTCCAGGACATACTTATGAGGATGTCAAAAATGCAGTTGGGGTCCTCATCGGA<br>GGACTTGAGTGGAATGGTAACACGGTTCGAGTCTCTGAAAATATACAGAGATTC<br>GCTTGGAGAAGCTGTGATGAGAATGGGAGACCTTCACTACCTCCAGAGCAGAAA<br>TGAGAAGTGGCGGGAACAATTGGGACAGAAATTTGAGGAAATAAGATGGTTAAT<br>TGAAGAAATACGACACAGATTGAAAGCGACAGAGAATAGTTTCGAACAAATAACA<br>TTTATGCAAGCCTTACAACTACTGCTTGAAGTAGAGCAAGAGATAAGAGCTTTCT<br>CGTTTCAGCTTATTTAATGATAAAAAACACCCTTGTTTCTACTAATACGAGACGAT<br>ATCG

Tasmania/H1N1	702 bp	CGTATTCGTCTCAGGGAGCGAAAGCAGGCAAACCATTTGAATGGATGTCAATCC
-PB1-5’		GACTCTATTGTTCTTAAAAGTTCCAGCGCAAAATGCCATAAGCACAACATTCCCT
		TATACTGGAGATCCTCCATACAGCCATGGAACAGGGACAGGGTACACTATGGAC
		ACAGTCAACAGAACACACCAATACTCAGAGAGGGGGAAGTGGACGACAAATACA
		GAAACTGGGGCTCCCCAGCTCAACCCAATTGAAGGACCACTACCTGAGGATAAT
		GAACCAAGTGGATATGCACAAACAGACTGTGTCCTGGAGGCTATGGCCTTCCTT
		GAAGAATCCCACCCAGGTATCTTTGAAAACTCCTGCCTTGAAACAATGGAAGCC
		GTTCAACAGACAAGGGTAGACAAACTAACTCAAGGTCGCCAGACTTATGATTGG
		ACATTAAACAGGAACCAACCGGCAGCAACTGCATTGGCCAACACCATAGAAGTC
		TTTAGATCGAACGGATTAACAGCTAATGAATCAGGAAGGCTAATAGATTTCCTCA
		AGGATGTGATGGAATCAATGGATAAGGAGGAAATGGAGATAACAACACACTTTC
		AAAGAAAAAGAAGAGTAAGGGACAACATGACCAAGAAAATGGTCACACAAAGAA
		CAATAGGGAAGAAAAAACAAAGAGTGAATAAGAGAGGCTACCTAATAAGAGC
Tasmania/H1N1	729 bp	AATAAGAGAGGCTACCTAATAAGAGCTCTGACATTGAACACGATGACCAAGGAT
-PB1-middle		GCAGAGAGAGGCAAATTAAAAAGAAGGGCTATTGCAACACCCGGGATGCAAATT
		AGAGGATTCGTGTATTTCGTTGAAACTTTAGCTAGAAGCATTTGCGAAAAACTTG
		AACAATCTGGACTTCCGGTTGGGGGTAATGAAAAGAAGGCCAAACTGGCAAATG
		TTGTGAGAAAAATGATGACTAATTCACAAGACACAGAGCTTTCTTTCACAATCACT
		GGAGACAACACTAAGTGGAATGAAAATCAAAACCCCCGAATGTTTTTGGCAATGA
		TTACATATATCACAAAGAACCAACCTGAATGGTTCAGAAACATCCTGAGCATCGC
		ACCAATAATGTTCTCAAACAAAATGGCAAGACTGGGAAAAGGATACATGTTCGAG
		AGTAAGAGAATGAAGCTCCGGACACAAATACCTGCAGAAATGCTAGCAAGCATT
		GACCTGAAGTATTTCAATGAATCAACAAGGAAGAAAATTGAGAAAATAAGGCCTC
		TTCTAATAGATGGAACAGCATCATTGAGCCCTGGAATGATGATGGGCATGTTCAA
		CATGCTAAGTACAGTTTTAGGAGTCTCGATACTGAATCTTGGACAAAAGAAATAC
		ACCAAGACAACGTACTGGTGGGATGGGCTCCAATCCTCAGACGATTTTGCCCTC
		ATAGTGAATGCACCAAAT

Tasmania/H1N1 -PB1-3’	994 bp	TGCCCTCATAGTGAATGCACCAAATCATGAGGGAATACAAGCAGGAGTGGATAG<br>ATTCTATAGGACCTGCAAGTTAGTGGGAATCAACATGAGCAAAAAGAAGTCCTAT<br>ATAAATAAAACAGGGACATTTGAATTCACTAGCTTCTTTTATCGATATGGATTTGT<br>GGCTAATTTTAGCATGGAGCTGCCAAGTTTTGGAGTGTCTGGAATAAACGAGTCA<br>GCTGACATGAGCATTGGAGTAACAGTGATAAAGAACAACATGATAAATAATGACC<br>TTGGACCAGCAACAGCCCAAATGGCTCTCCAATTGTTCATCAAAGATTACAGATA<br>CACGTATCGGTGCCATAGAGGAGACACACAAATCCAAACAAGAAGATCATTCGA<br>GATAAAGAAGCTGTGGGATCAAACCCAATCAAAGACAGGATTATTAGTATCAGAT<br>GGGGGACCAAACTTATACAATATCCGAAATCTTCACATCCCTGAAGTCTGCTTGA<br>AGTGGGAGCTGATGGATGATAATTATCGGGGAAGACTTTGTAATCCCCTGAATC<br>CCTTTGTCAGCCATAAAGAAATTGAATCTGTAAACAATGCTGTAGTAATGCCAGC<br>CCATGGTCCGGCCAAAAGTATGGAATATGATGCCGTTGCGACTACACACTCCTG<br>GATTCCCAAAAGGAACCGCTCTATTCTAAACACAAGCCAAAGGGGAATTCTTGAG<br>GATGAACAAATGTACCAGAAGTGCTGCAACTTGTTCGAGAAATTTTTCCCTAGTA<br>GTTCATATAGGAGACCGATTGGAATTTCTAGCATGGTGGAGGCCATGGTATCTA<br>GGGCCCGGATTGATGCCAGAATTGACTTCGAGTCTGGAAGGATTAAGAAGGAAG<br>AGTTCTCTGAGATCATGAAGATCTGTTCCACCATTGAAGAACTCAGACGGCAAAA<br>ATAATGAATTTAGCTTGTCCTTCATGAAAAAATGCCTTGTTTCTACTAATACGAGA<br>CGATATCG
Tasmania/H1N1 -PB2-5’	713 bp	CGTATTGGTCTCAGGGAGCGAAAGCAGGTCAATTATATTCAGCATGGAAAGAAT<br>AAAAGAACTACGGAATCTAATGTCGCAGTCTCGCACTCGCGAGATACTGACAAA<br>AACCACAGTGGACCATATGGCCATAATTAAGAAGTACACATCGGGGAGACAGGA<br>AAAGAACCCGTCACTTAGAATGAAATGGATGATGGCAATGAAATATCCAATTACT<br>GCTGACAAAAGGATAACAGAAATGGTTCCGGAGAGAAATGAACAAGGACAAACT<br>CTATGGAGTAAAATGAGTGATGCTGGATCAGACAGAGTGATGGTATCACCTTTG<br>GCTGTAACATGGTGGAATAGGAATGGACCCGTGACAGATACGGTCCATTACCCA<br>AAAGTGTACAAAACTTACTTCGACAAAGTCGAAAGGTTAAAACATGGAACCTTTG<br>GCCCTGTCCATTTTAGAAATCAAGTCAAAATACGAAGAAGAGTAGACATAAATCC<br>TGGTCATGCAGACCTCAGTGCCAAAGAGGCACAAGATGTAATTATGGAAGTTGT<br>TTTTCCCAATGAAGTGGGAGCCAGAATACTAACATCAGAATCACAGCTAACAATA<br>ACTAAAGAGAAAAAAGAAGAACTCCGAGATTGCAAAATTTCTCCCTTGATGGTCG<br>CATACATGCTAGAGAGAGAACTTGTGCGGAAAACAAGATTTCTCCCAGTTGCTG<br>GCGGAA

Tasmania/H1N1<br>-PB2-middle	729 bp	TTTCTCCCAGTTGCTGGCGGAACAAGCAGTATATACATTGAAGTTTTACATTTGA<br>CTCAAGGAACGTGTTGGGAACAAATGTACACTCCAGGTGGAGGAGTGAGGAATG<br>ACGATGTTGACCAAAGCCTAATTATTGCGGCCAGGAACATAGTAAGAAGAGCCG<br>CAGTGTCAGCGGATCCACTCGCATCTTTATTGGAGATGTGCCACAGCACGCAAA<br>TTGGCGGAACAAGGATGGTGGACATTCTTAGACAGAACCCGACTGAAGAACAAG<br>CTGTGGATATATGCAAAGCTGCAATGGGATTGAGAATCAGCTCATCTTTCAGCTT<br>TGGTGGCTTCACATTTAAAAGAACAAGCGGGTCGTCAGTCAAAAGAGAGGAAGA<br>GGTTCTTACAGGCAATCTCCAGACATTGAGAATAAGAGTACATGAGGGGTATGA<br>GGAGTTCACAATGGTGGGGAAAAGAGCAACAGCTATACTAAGAAAAGCAACCAG<br>AAGACTGGTTCAACTCATAGTGAGTGGAAGAGACGAACAGTCAATAGCTGAAGC<br>AATAATCGTGGCCATGGTGTTTTCACAAGAAGATTGCGTGATAAAAGCAGTTAGA<br>GGTGACCTGAATTTTGTCAACAGAGCAAATCAGCGGTTGAACCCCATGCATCAG<br>CTTTTAAGGCATTTTCAGAAAGATGCGAAAGTACTCTTTCAAAATTGGGGAGTTG<br>AACACATCGACAGTGTGATGGGA
Tasmania/H1N1<br>-PB2-3’	977 bp	AACACATCGACAGTGTGATGGGAATGGTTGGAGTATTACCAGATATGACTCCAA<br>GCACAGAGATGTCAATGAGAGGAATAAGAGTCAGCAAAATGGGTGTGGATGAAT<br>ACTCCAGTACAGAGAGGGTGGTGGTTAGCATTGATCGGTTTTTGAGAGTTCGTG<br>ACCAACGTGGGAATGTATTATTATCTCCTGAGGAGGTCAGTGAAACACAGGGAA<br>CTGAAAGACTGACCATAACTTATTCATCATCGATGATGTGGGAGATTAACGGCCC<br>TGAGTCGGTTTTGGTCAATACCTATCAATGGATCATCAGGAATTGGGAAGCTGTC<br>AAAATTCAATGGTCACAGAACCCTGCAATGTTGTACAACAAAATGGAATTTGAAC<br>CATTTCAATCTTTAGTCCCCAAGGCCACTAGAAGCCAATACAGTGGGTTTGTCAG<br>AACTCTATTCCAACAAATGAGAGACGTAATTGGGACATTTGACACTGCCCAGATA<br>ATAAAGCTTCTCCCTTTTGCAGCTGCTCCACCGAAGCAAAGCAGAATGCAGTTCT<br>CTTCACTGACTGTGAATGTGAGGGGATCAGGGATGAGAATACTTGTAAGGGGCA<br>ATTCTCCTGTATTCAACTACAACAAGACCACTAAAAGGCTAACAATTCTTGGAAAA<br>GATGCCGGCACTTTAATTGAAGACCCAGATGAAAGCACATCCGGAGTGGAGTCC<br>GCCGTCTTGAGAGGGTTCCTCATTATAGGTAAGGAAGACAGAAGATACGGACCA<br>GCATTAAGCATCAATGAACTGAGTAACCTTGCAAAAGGGGAAAAGGCTAATGTG<br>CTAATTGGGCAAGGAGACGTGGTGTTGGTAATGAAACGGAAACGGGACTCTAGT<br>ATACTTACTGACAGCCAGACAGCGACCAAAAGAATTCGGATGGCCATCAATTAAT<br>ACTGAATAGTTTAAAAACGACCTTGTTTCTACTAATACGAGACCATATCG

Tasmania/H1N1<br>-PA-5’	710 bp	CGTATTGGTCTCAGGGAGCGAAAGCAGGTACTGATTCAAAATGGAAGATTTTGT<br>GCGACAATGCTTCAACCCGATGATTGTCGAACTTGCAGAAAAAGCAATGAAAGA<br>GTATGGAGAGGATCTGAAAATTGAAACCAACAAATTTGCAGCAATATGCACTCAC<br>TTGGAGGTGTGTTTCATGTATTCAGATTTCCATTTCATCAATGAACAAGGCGAAT<br>CAATAGTAGTAGAACTTGACGATCCAAATGCACTGTTAAAACACAGATTTGAAAT<br>AATCGAGGGGAGAGACAGAACAATGGCCTGGACAGTAGTAAACAGTATCTGCAA<br>CACTACTGGAGCTGGAAAACCGAAGTTTTTACCGGATCTGTATGATTACAAAGAA<br>AACAGATTCATCGAAATTGGAGTGACAAGGAGAGAAGTCCACATATATTACCTTG<br>AAAAGGCCAATAAGATTAAATCAGAGAACACACACATTCACATTTTTTCATTCACT<br>GGGGAGGAAATGGCCACAAGGGCAGACTACACTCTCGACGAGGAAAGCAGGGC<br>TAGGATTAAAACCAGGCTATTTACCATAAGACAAGAAATGGCCAACAGAGGCCTC<br>TGGGATTCCTTTCGTCAGTCCGAAAGAGGCGAAGAAACAATTGAAGAAAAATTTG<br>AAATCACAGGAACTATGCGCAGGCTTGCCGACCAAAGTCTCCCACCGAACTTCT
Tasmania/H1N1<br>-PA-middle	736 bp	CAAAGTCTCCCACCGAACTTCTCCTGCCTTGAGAATTTTAGAGCCTATGTGGATG<br>GATTCGAACCGAACGGCTGCATTGAGGGCAAGCTTTCTCAAATGTCCAAAGAAG<br>TGAATGCCCAAATTGAACCTTTTCTGAAGACAACACCAAGACCAATCAAACTTCC<br>TAGTGGACCTCCTTGTTATCAGCGATCCAAATTCCTCCTGATGGATGCTTTGAAA<br>TTGAGCATTGAAGACCCAAGTCACGAAGGAGAAGGGATCCCATTATATGATGCA<br>ATCAAGTGCATAAAAACATTCTTTGGATGGAAAGAACCTTATATAGTCAAACCACA<br>CGAAAAGGGAATAAATTCAAATTACCTGCTGTCATGGAAGCAAGTACTGTCAGAA<br>TTGCAGGACATTGAAAATGAGGAGAAGATTCCAAGAACTAAAAACATGAAGAAAA<br>CGAGTCAACTGAAGTGGGCTCTTGGTGAAAACATGGCACCAGAGAAGGTAGACT<br>TTGAAAACTGCAGAGACATAAGCGATTTGAAGCAATATGACAGTGAAGAACCTGA<br>ATTAAGGTCACTTTCAAGCTGGATACAAAGTGAGTTCAACAAGGCCTGTGAGCTA<br>ACTGATTCAATCTGGATAGAGCTCGATGAAATTGGAGAGGACGTAGCCCCAATT<br>GAACACATTGCAAGCATGAGAAGGAATTATTTCACAGCAGAGGTGTCCCATTGTA<br>GAGCTACTGAATACATAATGAAAG

Tasmania/H1N1	872 bp	ATTGTAGAGCTACTGAATACATAATGAAAGGGGTATACATTAATACTGCCCTGCT
-PA-3’		CAATGCATCCTGTGCGGCAATGGACGATTTTCAACTAATTCCCATGATAAGCAAG
		TGCAGAACTAAAGAGGGAAGGCGAAAAACCAATTTATATGGATTCATCATAAAGG
		GAAGATCTCATCTGAGGAATGACACAGACGTGGTAAACTTTGTGAGCATGGAGT
		TTTCTCTCACAGATCCGAGACTTGAACCACATAAATGGGAGAAATATTGTGTCCT
		TGAGATAGGAGATATGTTACTAAGGAGTGCCATAGGCCAAATTTCAAGGCCGAT
		GTTCTTGTATGTGAGGACAAACGGAACATCAAAAGTCAAAATGAAATGGGGAATG
		GAGATGAGACGTTGCCTCCTTCAGTCACTCCAGCAGATCGAGAGCATGATTGAA
		GCCGAGTCCTCAGTTAAAGAGAAAGACATGACCAAAGAGTTTTTTGAGAATAAAT
		CAGAAGCATGGCCCATTGGGGAGTCCCCCAAAGGAGTGGAAGAAGGTTCCATT
		GGGAAAGTCTGTAGGACTCTATTGGCTAAGTCAGTATTCAATAGCCTGTATGCAT
		CACCACAATTGGAAGGATTTTCAGCAGAATCAAGAAAACTGCTCCTTATTGTTCA
		GGCTCTTAGGGACAAACTCGAACCTGGGACCTTTGATCTTGGGGGGCTATATGA
		AGCAATTGAGGAGTGCCTGATTAATGATCCCTGGGTTTTGCTCAATGCATCTTGG
		TTCAACTCCTTCCTGACACATGCATTAAAATAGTTATAGCAGTGCTACTATTTGTT
		ATCCATACTGTCCAAAAAAGTACCTTGTTTCTACTAATACGAGACCATATCG
Tasmania/H1N1	957 bp	CGTATTCGTCTCAGGGAGCAAAAGCAGGGGATAATTCTATTAACCATGAAGACTA
-HA-5’		TCATTGCTTTGAGCTACATTCTATGTCTTGTTTTCGCTCAAAAAATCCCTGGAAAT
		GACAATAGCACGGCAACGCTGTGCCTTGGGCACCATGCAGTACCAAACGGAAC
		GATAGTGAAAACAATCACAAATGACCGAATTGAAGTTACTAATGCTACTGAGTTG
		GTTCAGAATTCCTCAATAGGTGAAATATGCGACAGTCCTCATCAGATCCTTGATG
		GAGGGAACTGCACACTAATAGATGCTCTATTGGGGGACCCTCAGTGTGACGGCT
		TTCAAAATAAGGAATGGGACCTTTTTGTTGAACGAAGCAGAGCCAACAGCAACTG
		TTACCCTTATGATGTGCCGGATTATGCCTCCCTTAGGTCACTAGTTGCCTCATCC
		GGCACACTGGAGTTTAAAAATGAAAGCTTCAATTGGACTGGAGTCAAACAAAACG
		GAACAAGTTCTGCGTGCATAAGGGGATCTAGTAGTAGTTTTTTTAGTAGATTAAA
		TTGGTTGACCCACTTAAACTACAAATATCCAGCACTGAACGTGACTATGCCAAAC
		AAGGAACAATTTGACAAATTGTACATTTGGGGGGTTCACCACCCGAGGACGGAC
		AAGGACCAAATCTCCCTGTTTGCTCAACCATCAGGAAGAATCACAGTATCTACCA
		AAAGAAGCCAACAAGCTGTAATCCCAAATATCGGATCTAGACCCAGAATAAGGG
		ATATCCCTAGCAGAATAAGCATCTATTGGACAATAGTAAAACCGGGAGACATACT
		TTTGATTAACAGCACAGGGAATCTAATTGCTCCTAGGGGTTACTTCAAAATACGA

		AGTGGGAAAAGCTCAATAATGAGATCAGATGCACCCATTGGCAAATGCAAGTCT<br>GAATGCATCACTCCAAATGGAAGCATT
Tasmania/H1N1<br>-HA-3’	867 bp	AATGCATCACTCCAAATGGAAGCATTCCCAATGACAAACCGTTCCAAAATGTAAA<br>CAGGATCACATACGGGGCCTGTCCCAGATATGTCAAGCAAAGCACCCTGAAATT<br>GGCAACAGGAATGCGAAATGTACCAGAGAAACAAACCAGAGGCATATTTGGCGC<br>AATAGCGGGTTTCATAGAAAATGGATGGGAGGGAATGGTGGATGGTTGGTACGG<br>TTTCAGGCATCAAAATTCTGAGGGAAGAGGACAAGCAGCAGATCTCAAAAGCAC<br>TCAAGCAGCAATCGATCAAATCAATGGGAAGCTGAATCGATTGATCGGAAAAAC<br>CAACGAGAAATTCCATCAGATTGAAAAAGAATTCTCAGAAGTAGAAGGAAGAGTT<br>CAAGACCTTGAGAAATATGTTGAGGACACTAAAATAGATCTCTGGTCATACAACG<br>CTGAGCTTCTTGTTGCCCTGGAGAACCAACATACAATTGACCTAACTGACTCAGA<br>AATGAACAAACTGTTTGAAAAAACAAAGAAGCAACTGAGGGAAAATGCTGAGGAT<br>ATGGGAAATGGTTGTTTCAAAATATACCACAAATGTGACAATGCCTGCATAGGAT<br>CAATAAGAAATGAAACTTATGACCACAATGTGTACAGGGATGAAGCATTAAACAA<br>CCGGTTCCAGATCAAGGGAGTTGAGCTGAAGTCAGGGTACAAAGATTGGATCCT<br>ATGGATTTCCTTTGCCATGTCATGTTTTTTGCTTTGTATTGCTTTGTTGGGGTTCA<br>TCATGTGGGCCTGCCAAAAGGGCAACATTAGATGCAACATTTGCATTTGAGTGTA<br>TTAGTAATTAAAAACACCCTTGTTTCTACTAATACGAGACGATATCG
Tasmania/H1N1<br>-NP-5’	713 bp	CGTATTCGTCTCAGGGAGCAAAAGCAGGGTAGATAATCACTCACTGAGTGACAT<br>CAAAATCATGGCGTCCCAAGGCACCAAACGGTCTTATGAACAGATGGAAACTGA<br>TGGAGATCGCCAGAATGCAACTGAGATTAGGGCATCCGTCGGGAAGATGATTGA<br>TGGAATTGGGAGATTCTACATCCAAATGTGCACTGAACTTAAACTCAGTGATCAT<br>GAAGGACGGTTGATCCAAAATAGCTTGACAATAGAGAAAATGGTACTCTCTGCTT<br>TTGATGAAAGAAGGAATAAATACCTGGAAGAACACCCCAGCGCGGGGAAAGATC<br>CCAAGAAAACTGGGGGGCCCATATACAGAAGAGTCGATGGAAAATGGATGAGG<br>GAACTCGTCCTTTATGACAAAGAAGAAATAAGGCGAATCTGGCGCCAAGCCAAC<br>AATGGTGAGGATGCTACATCTGGTCTAACCCACATAATGATTTGGCATTCCAATT<br>TGAATGATGCGACATACCAGAGGACAAGAGCTCTTGTTCGGACTGGAATGGATC<br>CCAGAATGTGCTCTCTGATGCAGGGATCGACTCTCCCTAGAAGGTCCGGAGCTG<br>CAGGTGCTGCAGTCAAAGGAATCGGAACAATGGTGATGGAACTGATCAGAATGA<br>TCAAACGGGGGATCAACGATCGAAATTTTTGGAGAGGTGAGAATGGGCGGAAAA<br>CAAGAAGTG

Tasmania/H1N1<br>-NP-3’	909 bp	AGAATGGGCGGAAAACAAGAAGTGCTTATGAGAGAATGTGCAACATTCTTAAAG<br>GAAAATTTCAAACAGCTGCACAAAGAGCAATGGTGGATCAAGTCAGAGAAAGTC<br>GGAACCCAGGAAACGCTGAGATCGAAGATCTCATATTTTTAGCAAGATCTGCACT<br>GATATTGAGAGGATCAGTTGCTCACAAATCTTGCCTACCTGCCTGTGCATATGGA<br>CCTGCAGTATCCAGTGGTTACGACTTTGAAAAAGAGGGATATTCCTTGGTGGGA<br>ATAGACCCTTTCAAACTACTTCAAAATAGCCAAATATACAGCTTAATCAGACCTAA<br>TGAGAATCCAGCACACAAGAGTCAGCTGGTGTGGATGGCATGCCATTCTGCTGC<br>ATTTGAAGATTTAAGATTGTTAAGCTTCATCAGAGGGACAAAAGTATCTCCTCGG<br>GGGAAACTGTCAACTAGAGGAGTACAAATTGCTTCAAATGAGAACATGGATAATA<br>TGGGATCAAGCACTCTTGAACTGAGAAGCGGGTACTGGGCCATAAGGACCAGG<br>AGTGGAGGAAACACTAATCAACAGAGGGCCTCCGCAGGCCAAACCAGTGTGCA<br>ACCTACGTTTTCTGTACAAAGAAACATCCCATTTGAAAAGTCAACCATCATGGCA<br>GCATTCACTGGAAATACGGAGGGAAGAACTTCAGACATGAGGGCAGAAATCATA<br>AGGATGATGGAAGGTGCAAAACCAGAAGAAGTGTCATTCCGGGGGAGGGGAGT<br>TTTCGAGCTCTCAGACGAGAAGGCAGCGAACCCGATCGTGCCCTCTTTTGATAT<br>GAGTAACGAAGGATCTTATTTCTTCGGAGACAATGCAGAAGAGTACGACAATTAA<br>AGAAAAATACCCTTGTTTCTACTAATACGAGACGATATCG
Tasmania/H1N1<br>-NA-5’	641 bp	CGTATTGGTCTCAGGGAGCAAAAGCAGGAGTGAAAATGAATCCAAATCAAAAGA<br>TAATAACGATTGGCTCTGTTTCTCTCACAATTTCCACAATATGCTTCTTCATGCAA<br>ATTGCCATCCTGATAACTACTGTAACATTGCATTTCAAGCAATATGAATTCAACTC<br>CCCCCCAAATAACCAAGTGATGCTGTGTGAACCAACAATAATAGAAAGAAACATG<br>ACAGAGATAGTGTATTTGACCAACACCACCATAGAGAAGGAAATATGCCCCAAAC<br>CAGCAGAATACAGAAATTGGTCAAAACCGCAATGTGGCATTACAGGATTTGCAC<br>CTTTCTCTAAGGACAATTCGATTAGGCTTTCCGCTGGTGGGGACATCTGGGTGA<br>CAAGAGAACCTTATGTGTCATGCGATCTTGACAAGTGTTATCAATTTGCCCTTGG<br>ACAGGGAACAACACTAAACAACGTGCATTCAAATAACACAGTACGTGATAGAACC<br>CCTTATCGGACTCTATTGATGAATGAGTTGGGTGTTCCTTTCCATCTGGGGACCA<br>AGCAAGTGTGCATAGCATGGTCCAGCTCAAGTTGTCACGATGGAAAAGCATGGC<br>TGCATGTTTGTATAACGGGGGATGATAAAAATGCAACT

Tasmania/H1N1<br>-NA-3’	883 bp	AACGGGGGATGATAAAAATGCAACTGCTAGCTTCATTTACAATGGGAGGCTTGTA<br>GATAGTGTTGTTTCATGGTCCAACGATATTCTCAGAACCCAGGAGTCAGAATGCG<br>TTTGTATCAATGGAACTTGTACAGTAGTAATGACTGATGGAAATGCTACAGGAAA<br>AGCTGATACTAAAATACTATTCATTGAGGAGGGGAAAATCGTTCATACTAGCAAA<br>TTGTCAGGAAGTGCTCAGCATGTCGAAGAGTGCTCTTGCTATCCTCGATATCCTG<br>GTGTCAGATGTGTCTGCAGAGACAACTGGAAAGGATCCAACCGGCCCATCATAG<br>ATATAAACATAAAGGATCATAGCATTGTTTCCAGGTATGTGTGTTCTGGACTTGTT<br>GGAGACACACCCAGAAAAAGCGACAGCTCCAGCAGTAGCCATTGTTTGAACCCT<br>AACAATGAAAAAGGTGATCATGGAGTGAAAGGCTGGGCCTTTGATGATGGAAAT<br>GACGTGTGGATGGGGAGAACAATCAACGAGACGTCACGCTTAGGGTATGAAAC<br>CTTCAAAGTCGTTGAAGGCTGGTCCAACCCTAAGTCCAAATTGCAGATAAATAGG<br>CAAGTCATAGTTGACAGAGGTGATAGGTCCGGTTATTCTGGTATTTTCTCTGTTG<br>AAGGCAAAAGCTGCATCAATCGGTGCTTTTATGTGGAGTTGATTAGGGGAAGAA<br>AAGAGGAAACTGAAGTCTTGTGGACCTCAAACAGTATTGTTGTGTTTTGTGGCAC<br>CTCAGGTACATATGGAACAGGCTCATGGCCTGATGGGGCGAACCTCAGTCTCAT<br>GCATATATAAGCTTTCGCAATTTTAGAAAAAACTCCTTGTTTCTACTAATACGAGA<br>CCATATCG
Tasmania/H1N1<br>-M-5’	476 bp	CGTATTCGTCTCAGGGAGCAAAAGCAGGTAGATATTGAAAGATGAGCCTTCTTAC<br>CGAGGTCGAAACGTATGTTCTCTCTATCGTTCCATCAGGCCCCCTCAAAGCCGA<br>GATCGCGCAGAGACTTGAAGATGTCTTTGCTGGGAAAAACACAGATCTTGAGGC<br>TCTCATGGAATGGTTAAAGACAAGACCAATTCTGTCACCTTTGACTAAGGGGATT<br>TTAGGGTTTGTTTTCACGCTCACCGTGCCCAGTGAGCGAGGACTGCAGCGTAGA<br>CGCTTTGTCCAAAATGCCCTCAATGGGAATGGAGACCCAAATAACATGGACAAA<br>GCAGTTAAACTGTATAGGAAACTTAAGAGGGAGATAACGTTCCACGGGGCCAAA<br>GAAATAGCTCTCAGTTATTCTGCTGGTGCACTTGCCAGTTGCATGGGCCTCATAT<br>ACAATAGGATGGGGGCTGTAACCACTGAAGTGGCATTTGGC

Tasmania/H1N1<br>-M-3’	606 bp	AACCACTGAAGTGGCATTTGGCCTGGTGTGTGCAACATGTGAGCAGATTGCTGA<br>TTCCCAGCACAGGTCTCATAGGCAGATGGTGGCAACAACCAATCCATTAATAAAA<br>CATGAGAACAGAATGGTTTTGGCCAGCACTACAGCTAAGGCTATGGAGCAAATG<br>GCTGGATCAAGTGAGCAAGCAGCAGAGGCCATGGAGATTGCTAGTCAGGCCAG<br>GCAGATGGTGCAGGCAATGAGAGCCATTGGGACTCATCCTAGTTCCAGCACTGG<br>TCTAAGAGATGATCTTCTTGAAAATTTGCAGACCTATCAGAAACGAATGGGGGTG<br>CAGATGCAACGATTCAAGTGACCCGCTTGTTGTTGCCGCGAATATCATTGGGAT<br>CTTGCACTTGATATTGTGGATTCTTGATCGTCTTTTTTTCAAATGCGTCTATCGAC<br>TCTTCAAACACGGCCTTAAAAGAGGCCATTCTACGGAAGGTGTACCTGAGTCTAT<br>GAGGGAAGAATACCGAAAGGAACAGCAGAATGCTGTGGATGCTGACGACAGTC<br>ATTTTGTCAGCATAGAATTGGAGTAAAAAACTACCTTGTTTCTACTAATACGAGAC<br>GATATCG
Tasmania/H1N1<br>-NS-5’	474 bp	CGTATTCGTCTCAGGGAGCAAAAGCAGGGTGACAAAGACATAATGGATTCCAAC<br>ACTGTGTCAAGTTTCCAGGTAGATTGCTTTCTTTGGCATATCCGGAAACAAGTTG<br>TGGACCAAAAACTGAGTGATGCCCCATTCCTCGATCGGCTTCGCCGAGATCAGA<br>GGTCCCTAAGGGGAAGAGGCAATACTCTCGGTCTAGACATCAAATCAGCCACCC<br>ATGTTGGAAAGCAAATCGTAGAAAAGATTCTGAAAGGAGAATCTGATGAGGCACT<br>TAAAATGACCATGGTCTCAACACCTGCTTCGCGATACATAACTGACATGACTATT<br>GAGGAATTGTCAAGAAACTGGTTCATGCTAATGCCCAAGCAGAAGGTGGAAGGA<br>CCTCTTTGCATCAGAATGGACCAGGCAATCATGGAGAAAAACATCATGTTAAAAG<br>CGAATTTCAATGTGATTTTTGGCCGGCTAGAGACCATA
Tasmania/H1N1<br>-NS-3’	470 bp	TTTGGCCGGCTAGAGACCATAGTATTGCTAAGGGCTTTCACCGAAGAGGGAGCA<br>ATTGTTGGCGAAATCTCACCATTGCCTTCTTTTCCAGGACATACTATTGAGGATG<br>TCAAAAATGCAATTGGGGTCCTCATCGGAGGACTTGAATGGAATGATAACACAGT<br>TCGAGTCTCTAAAAATCTACAGAGATTCGCTTGGAGAAGCAGTCATGAGAATGG<br>GGGACCTCCACTTACTCCAAAACAGAAACGGGAAATGGCGAGAACAGCTAGGTC<br>AGAAGTTTGAAGAGATAAGATGGCTGATTGAAGAGGTGAGACACAGATTAAGAA<br>CAACTGAAAATAGCTTTGAACAAATAACATTCATGCAAGCATTACAACTACTGTTT<br>GAAGTGGAACAGGAGATAAGAACTTTCTCATTTCAGCTTATTTAATGATAAAAAC<br>ACCCTTGTTTCTACTAATACGAGACGATATCG

maH3N2-HA-1 872 bp

CGTATTGGTCTCAGGGAGCAAAAGCAGGGGATAATTCTATTAATCATGAAGACCA
TCATTGCTTTGAGCTACATTTTCTGTCTGGCTCTCGGCCAAGACCTTCCAGGAAA
TGACAACAGCACAGCAACGCTGTGCCTGGGACATCATGCGGTGCCAAACGGAA
CACTAGTGAAAACAATCACAGATGATCAGATTGAAGTGACTAATGCTACTGAGCT
AGTTCAGAGCTCCTCAACGGGGAAAATATGCAACAATCCTCATCGAATCCTTGAT
GGAATAGACTGCACACTGATAGATGCTCTATTGGGGGACCCTCATTGTGATGTTT
TTCAAAATGAGACATGGGACCTTTTCGTTGAACGCAGCAAAGCTTTCAGCAACTG
TTACCCTTATGATGTGCCAGATTATGCCTCCCTTAGGTCACTAGTTGCCTCGTCA
GGCACTCTGGAGTTTATCACTGAGGGTTTCACTTGGACTGGGGTCACTCAGAAT
GGGGGAAGCAATGCTTGCAAAAGGGGACCTGGTAGCGGTTTTTTCAGTAGACTG
AACTGGTTGACCAAATCAGGAAGCACATATCCAGTGCTGAACGTGACTATGCCA
AACAATGACAATTTTGACAAACTATACATTTGGGGGGTTCACCACCCGAGCACGA
ACCAAGAACAAACCAGCCTGTATGTTCAAGCATCAGGGAGAGTCACAGTCTCTA
CCAGAAGAAGCCAGCAAACTATAATCCCGAATATCTGGTCCAGACCCTGGGTAA
GGGGTCTGTCTAGTAGAATAAGCATCTATTGGACAATAGTTAAGCCGGGAGACG
TACTGGTAATTAATAGTAATGGGAACCTAATCGCTCCTCGGGGTTATTTCAAAAT

---

maH3N2-HA-2 953 bp

AATCGCTCCTCGGGGTTATTTCAAAATGCGCACTGGGAAAAGCTCAATAATGAG
GTCAGATGCACCTATTGATACCTGTATTTCTGAATGCATCACTCCAAATGGAAGC
ATTCCCAATGACAAGCCCTTTCAAAACGTAAACAAGATCACATATGGAGCATGCC
CCAAGTATGTTAAGCAAAACACCCTGAAGTTGGCAACAGGGATGCGGAATGTAC
CAGAGAAACAAACTAGAGGCCTATTCGGCGCAATAGCAGGTTTCATAGAAAATG
GTTGGGAGGGAATGATAGACGGTTGGTACGGTTTCAGGCATCAAAATTCTGAGG
GCACAGGACAAGCAGCAGATCTTAAAAGCACTCAAGCAGCCATCGACCAAATCA
ATGGGAAATTGAACAGGGTAATCGAGAAGACGAACGAGAAATTCCATCAAATCG
AAAAGGAATTCTCAGAAGTAGAAGGAAGAATTCAGGACCTCGAGAAATACGTTG
AAGACACTAAAATAGATCTCTGGTCTTACAATGCGGAGCTTCTTGTCGCTCTGGA
GAATCAACATACAATTGACCTGACTGACTCGGAAATGAACAAGCTGTTTGAAAAA
ACAAGGAGGCAACTGAGGGAAAATGCTGAAGACATGGGCAATGGTTGCTTCAAA
ATATACCACAAATGTGACAACGCTTGCATAGAGTCAATCAGAAATGGGAATTATG
ACCATGATGTATACAGAGACGAAGCATTAAACAACCGGTTTCAGATCAAAGGTGT
TGAACTGAAGTCTGGATACAAAGACTGGATCCTGTGGATTTCCTTTGCCATATCA
TGCTTTTTGCTTTGTGTTGTTTTGCTGGGGTTCATCATGTGGGCCTGCCAGAGAG

		GCAACATTAGGTGCAACATTTGCATTTGAGTGTATTAGTAATTAAAAACACCCTTG<br>TTTCTACTAATACGAGACCATATCG
maH3N2-NA	1,500<br>bp	CGTATTCGTCTCAGGGAGCAAAAGCAGGAGTGAAAATGAATCCAAATCAAAAGA<br>TAATAACAATTGGCTCTGTCTCTCTCACCATTGCAACAGTATGCTTCCTCATGCA<br>GATTGCCATCCTGGTAACTACTGTAACATTGCATTTTAAGCAATATGAGTGCGAC<br>TCCCCCGCGAGCAACCAAGTAATGCCGTGTGAACCAATAATAATAGAAAGGAAC<br>ATAACAGAGATAGTGTATTTGAATAACACCACCATAGAGAAAGAGATATGCCCCA<br>AAGTAGTGGAATACAGAAATTGGTCAAAGCCGCAATGTCAAATTACAGGATTTGC<br>ACCTTTTTCTAAGGACAATTCAATCCGGCTTTCTGCTGGTGGGGACATTTGGGTG<br>ACGAGAGAACCTTATGTGTCATGCGATCATGGCAAGTGTTATCAATTTGCACTCG<br>GGCAGGGGACCACACTAGACAACAAACATTCAAATGACACAATACATGATAGAAT<br>CCCTCATCGAACCCTATTAATGAATGAGTTGGGTGTTCCATTTCATTTAGGAACC<br>AGGCAAGTGTGTATAGCATGGTCCAGCTCAAGTTGTCACGATGGAAAAGCATGG<br>CTGCATGTTTGTATCACTGGGGATGACAAAAATGCAACTGCTAGCTTCATTTATG<br>ACGGGAGGCTTGTGGACAGTATTGGTTCATGGTCTCAAAATATCCTCAGAACCC<br>AGGAGTCGGAATGCGTTTGTATCAATGGGACTTGCACAGTAGTAATGACTGATG<br>GAAGTGCTTCAGGAAGAGCCGATACTAGAATACTATTCATTGAAGAGGGGAAAA<br>TTGTCCATATTAGCCCATTGTCAGGAAGTGCTCAGCATGTAGAAGAGTGTTCCTG<br>TTATCCTAGATATCCTGGCGTCAGATGTATCTGCAGAGACAACTGGAAAGGCTCT<br>AATAGGCCCGTCGTAGACATAAATATGGAAGATTATAGCATTGATTCCAGTTATG<br>TGTGCTCAGGGCTTGTTGGCGACACACCTAGAAACGACGACAGATCTAGCAATA<br>GCAATTGCAGGAATCCTAACAATGAGAGAGGGAATCAAGGAGTGAAAGGCTGG<br>GCCTTTGACAATGGAGATGACGTGTGGATGGGAAGAACGATCAGCAAGGATTTA<br>CGCTCAGGTTATGAAACTTTCAAAGTCATTGGTGGTTGGTCCACACCTAATTCCA<br>AATCGCAGATCAATAGACAAGTCATAGTTGACAGCGATAATCGGTCAGGTTACTC<br>TGGTATTTTCTCTGTTGAGGGCAAAAGCTGCATCAATAGGTGCTTTTATGTGGAG<br>TTGATAAGGGGAAGGAAACAGGAGACTAGAGTGTGGTGGACCTCAAACAGTATT<br>GTTGTGTTTTGTGGCACTTCAGGTACCTATGGAACAGGCTCATGGCCTGATGGG<br>GCGAACATCAATTTCATGCATATATAAGCTTTCGCAATTTTAGAAAAAAACTCCTT<br>GTTTCTACTAATACGAGACGATATCG

Supplementary references

1. Zhou B, Meliopoulos VA, Wang W, Lin XD, Stucker KM, Halpin RA, Stockwell TB, Schultz-Cherry S, Wentworth DE. 2016. Reversion of Cold-Adapted Live Attenuated Influenza Vaccine into a Pathogenic Virus. Journal of Virology 90:8454-8463.
2. Naito T, Mori K, Ushirogawa H, Takizawa N, Nobusawa E, Odagiri T, Tashiro M, Ohniwa RL, Nagata K, Saito M. 2017. Generation of a Genetically Stable High-Fidelity Influenza Vaccine Strain. J Virol 91:e01073-16.
3. Ainai A, Hasegawa H, Obuchi M, Odagiri T, Ujike M, Shirakura M, Nobusawa E, Tashiro M, Asanuma H. 2015. Host Adaptation and the Alteration of Viral Properties of the First Influenza A/H1N1pdm09 Virus Isolated in Japan. PLoS One 10:e0130208.
4. Robertson JS, Nicolson C, Harvey R, Johnson R, Major D, Guilfoyle K, Roseby S, Newman R, Collin R, Wallis C, Engelhardt OG, Wood JM, Le J, Manojkumar R, Pokorny BA, Silverman J, Devis R, Bucher D, Verity E, Agius C, Camuglia S, Ong C, Rockman S, Curtis A, Schoofs P, Zoueva O, Xie H, Li X, Lin Z, Ye Z, Chen LM, O'Neill E, Balish A, Lipatov AS, Guo Z, Isakova I, Davis CT, Rivailler P, Gustin KM, Belser JA, Maines TR, Tumpey TM, Xu X, Katz JM, Klimov A, Cox NJ, Donis RO. 2011. The development of vaccine viruses against pandemic A(H1N1) influenza. Vaccine 29:1836-43.
